# Forelimb musculature and osteological correlates in Sauropodomorpha (Dinosauria, Saurischia)

**DOI:** 10.1371/journal.pone.0198988

**Published:** 2018-07-05

**Authors:** Alejandro Otero

**Affiliations:** CONICET - División Paleontología de Vertebrados, Museo de La Plata, La Plata, Argentina; University of Chicago, UNITED STATES

## Abstract

This contribution presents the forelimb muscular arrangement of sauropodomorph dinosaurs as inferred by comparisons with living archosaurs (crocodiles and birds) following the Extant Phylogenetic Bracket approach. Forty-one muscles were reconstructed, including lower limb and manus musculature, which prior information available was scarce for sauropodomorphs. A strong emphasis was placed on osteological correlates (such as tubercles, ridges and striae) and comparisons with primitive archosauromorphs are included in order to track these correlates throughout the clade. This should help to elucidate how widespread among other archosaurian groups are these osteological correlates identified in Sauropodomorpha. The ultimate goal of this contribution was to provide an exhaustive guide to muscular identification in fossil archosaurs and to offer solid anatomical bases for future studies based on osteology, myology, functional morphology and systematics.

## Introduction

**Fig 1:**
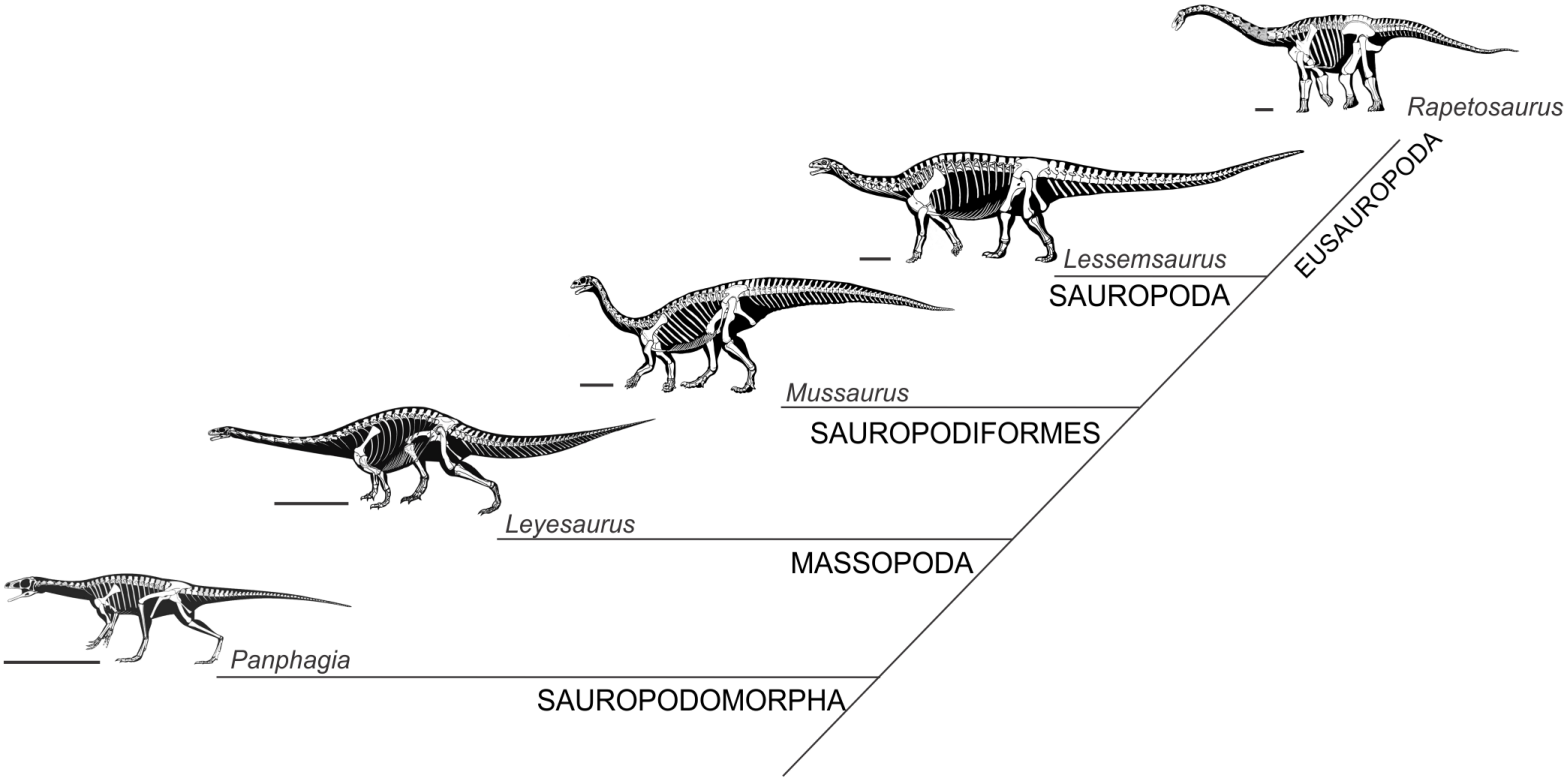
Body plans among Sauropodomorpha. Phylogenetic relationships based on Otero *et al*. [48]. *Panphagia* based on Martínez and Alcober (Fig 2 in [129]); *Leyesaurus* based on Apaldetti *et al*. (Fig 2 in [130]); *Mussaurus* and *Lessemsaurus* reconstructed by Tec. Jorge González; *Rapetosaurus* based on Wilson *et al*. (Fig 1 in [131]). Scale bar: 50 cm.

Sauropodomorpha is one of the most successful dinosaurian groups, both in taxonomic diversity and geographical distribution, with almost 200 valid species spread across all continental landmasses [1–3] and ranging from the Late Triassic (Carnian, ca. 225 mya) to Late Cretaceous (Maastrichthian, 64 mya). Sauropodomorphs experienced two main peaks of diversity, i.e. in the Late Triassic and Late Jurassic [4]. These peaks correspond to the radiation of the two main groups constituting the clade: basal sauropodomorphs (so called ‘Prosauropoda’) and sauropods. Both groups are characterized by novel modifications in the appendicular skeleton which, in part, underlay their successful diversification [5–8]. The forelimbs of sauropodomorph dinosaurs are a particularly interesting matter of study because drastic modifications occurred in that region. In this sense, basal sauropodomorphs are regarded as (at least) facultatively bipedal with relatively short forelimbs and a highly specialized manus bearing a robust digit one, which would have been used for additional functions beyond locomotion and support [7, 9]. In spite of the size disparity between small primitive forms like the gracile *Saturnalia* (about 1.5 m) and the much more massive *Lessemsaurus* (about 8 m), the basic forelimb morphology was maintained throughout basal sauropodomorph evolution. On the other hand, sauropods evolved an obligatory quadrupedal locomotion and deep forelimb specializations, such as a ‘U’-shaped metacarpus, progressive reduction of manual phalanges and primitive loss of the olecranon process which allowed the evolution of graviportalism and extreme body sizes [10–13] (Fig 1).

Previous published studies on forelimb musculoskeletal morphology and arrangement in sauropodomorphs were focused on phylogenetic extremes among the clade, namely the basalmost sauropodomorph *Saturnalia* [14] and the derived titanosaur *Opisthocoelicaudia* [15]. However, published information from intermediate forms was mostly lacking (although forelimb muscles were recently inferred in *Mussaurus patagonicus* [9] and some shoulder muscles were previously inferred in three neosauropods by Schwarz *et al*. [16]). This is surprising considering that sauropodomorph dinosaurs experienced drastic modifications in their appendicular skeleton throughout the evolution of the clade, particularly during the transition from basal sauropodomorphs to sauropods, in which quadrupedal graviportalism was achieved [7, 8]. In this regard two questions arise, i.e. what was the forelimb muscular arrangement of the ‘core’ non-sauropod sauropodomorphs, and how did the morphology of osteological correlates shift during sauropodomorph evolution?

In this contribution I present the inferred shoulder and forelimb musculature of sauropodomorph dinosaurs. The aims of this work are: 1) to provide a complete guide to muscular arrangement among Sauropodomorpha through phylogenetic inference on living representatives; 2) to describe, compare and figure the osteological correlates associated with the inferred musculature; 3) to explore how widespread among other archosaurian groups are the osteological correlates present in Sauropodomorpha; 4) to compare and discuss the muscular arrangement observed in sauropodomorphs with that previously described for other dinosaurs. The ultimate aim of this work is to offer solid anatomical bases for future studies based on osteology, myology, functional morphology and systematics.

## Materials and methods

### Materials

The anatomical framework was provided by living crocodiles and birds. The inference and reconstruction of musculature were carried out through dissections on fresh material and by direct observations of osteological data of *Caiman latirostris* and *Crocodylus niloticus* (Crocodylia) and *Gallus gallus* (Aves). Particular emphasis was placed on osteological correlates of dissected specimens; then patterns of muscle attachment and structures observed on fresh material were, if possible, interpreted on fossil taxa and contrasted with the literature on forelimb musculature of extant and extinct archosaurs.

Previous works on soft tissue anatomy of lepidosaurs [17, 18], crocodiles and birds were used for comparisons and homology hypotheses. Most previous contributions dealing with crocodilian forelimb myology were based on *Alligator mississippiensis* [19–23], but also *Caiman crocodilus* [24] and *Crocodylus porosus* [25]. In the case of birds, the Nomina Anatomica Avium was used as a reference [26] aided by comparisons with works of Remes [17], Jasinoski *et al*. [22], McKitrick [27] and Meyers [28]. Muscle homology among living reptiles are depicted in Table 1.

**Table 1:** Forelimb muscle homologies among reptiles.

Lepidosauria	Crocodilia	Aves
M. trapezius	M. trapezius	M. cucullaris
M. latissimus dorsi	M. latissimus dorsi	M. latissimus dorsi cranialis
		M. latissimus dorsi caudalis
M. levator scapulae	M. levator scapulae	Absent
Absent	M. rhomboideus	M. rhomboideus superficialis
		M. rhomboideus profundus
M. serratus superficialis	M. serratus superficialis	M. serratus superficialis
M. serratus profundus	M. serratus profundus	M. serratus profundus
M. pectoralis	M. pectoralis	M. pectoralis pars thoracicus
		M. pectoralis pars propatagialis
		M. pectoralis pars abdominalis
M. costocoracoideus	M. costocoracoideus profundus	absent
Absent	M. costocoracoideus superficiales	absent
M. sternocoracoideus	absent	M. sternocoracoideus
M. deltoideus scapularis	M. deltoideus scapularis	absent
M. deltoideus clavicularis	M. deltoideus clavicularis	M. deltoideus propatagialis M. deltoideus major M. deltoideus minor
Absent	M. teres major	absent
M. subscapularis	M. subscapularis	M. subscapularis
M. subcoracoideus	absent	M. subcoracoideus
M. scapulohumeralis cranialis	absent	M. scapulohumeralis cranialis
M. scapulohumeralis caudalis	M. scapulohumeralis	M. scapulohumeralis caudalis
M. supracoracoideus	M. supracoracoideus longus	M. supracoracoideus
	M. supracoracoideus intermedius	
	M. supracoracoideus brevis	
M. coracobrachialis brevis	M. coracobrachialis brevis dorsalis	M. coracobrachialis cranialis
	M. coracobrachialis brevis dorsalis	M. coracobrachialis caudalis
M. coracobrachialis longus	Absent	Absent
M triceps brachii caput scapulare	M triceps brachii caput scapulare	M. scapulotriceps
M triceps brachii caput coracoideum	M. triceps brachii caput scapulocoracoideus	M. coracotriceps
M triceps brachii capiti humerales	M triceps brachii capiti humerales	M. humerotriceps
M. biceps brachii	M. biceps brachii	M. biceps brachii
M. humeroradialis	M. humeroradialis	absent
M. brachialis	M. brachialis	M. brachialis
M. supinator	M. supinator	M. supinator
M. flexor ulnaris	M. flexor ulnaris	M. ectepicondylo ulnaris
M. abductor radialis	M. abductor radialis	absent
M. extensor carpi radialis superficialis	M. extensor carpi radialis	M. extensor carpi radialis
M. extensor carpi radialis intermedius		
M. extensor digitorum longus	M. extensor digitorum longus	M. extensor digitorum communis
Flexor carpi ulnaris	Flexor carpi ulnaris	Flexor carpi ulnaris
Extensor carpi ulnaris	absent	Extensor carpi ulnaris
M. pronator teres	M. pronator teres	M. pronator superficialis
		M. pronator profundus
M. pronator quadratus	M. pronator quadratus	M. ulnometacarpalis ventralis
Absent	Mm. extensores metacarpi	absent
M. abductor pollicis longus	M. abductor pollicis longus	M. extensor longus alulae
M. flexor digitorum longus	M. flexor digitorum longus	M. flexor digitorum superficialis
		M. flexor digitorum profundus
M. extensores digitorum superficiales	M. extensores digitorum superficiales	M. extensor longus digiti majoris
		M. ulnometacarpalis dorsalis
M. extensores digitorum profundus	M. extensores digitorum profundus	M. extensor longus digiti majoris pars distalis
		M. extensor brevis alulae
M. flexores digitorum superficiales	M. flexores digitorum superficiales	M. flexor alulae
M. flexores digitorum profundus	M. flexores digitorum profundus	M. abductor digiti majoris
		M. flexor digiti minoris M. adductor alulae

Lepidosauria based on Zaaf [17] and Remes [18] Crocodilia based on Meers [19] and Remes [18] Aves based on Vanden Berge and Zweers [26] and Remes [18].

An exhaustive review of sauropodomorph dinosaurs was carried out, including an extensive revision of material of basal archosauromophs, basal dinosauriforms, theropods and ornithischians in order to identify the primitive presence of osteological correlates and to track their changes along the clade. Most of the material was studied by a first hand revision, aided by bibliography when access to the material was not possible. The revised material included in this study (listed in Table 2) is deposited in an accessible, permanent repository and no permits were required for the described study, which complied with all relevant regulations.

**Table 2:** Source of comparative data used in this study.

Taxon	Source
**Non-dinosaurian Archosauromorpha**	
*Batrachotomus kupferzellensis*	Gower and Schoch [104]; Nesbitt [101]
*Caiman yacare*	La Plata University, #1
*Crocodylus sp*.	La Plata University, #2
*Crocodylus niloticus*	MLP-H-1
*Euparkeria capensis*	SAM-K13666
*Garjainia prima*	Ezcurra [48]
*Lewisuchus admixtus*	Bittencourt *et al*. [108]
*Notosuchus terrestris*	Pol [127]
*Pissarrachampsa sera*	Godoy *et al*. [116]
*Sacisaurus agudoensis*	Langer and Ferigolo [111]
*Silesaurus opolensis*	ZPAL Ab III-404/8
*Simosuchus clarki*	Sertich and Groenke [95]
*Trilophosaurus buettneri*	Leardi [114]
*Vancleavea campi*	Nesbitt *et al*. [107]
*Yacarerani boliviensis*	Leardi *et al*. [94]
**Ornithischia**	
*Eocursor parvus*	Butler [110]
*Heterodontosaurus tucki*	Santa Luca [88]
**Theropoda**	
*Herrerasaurus ischigualastensis*	PVSJ 373; Sereno [109]
*Megalosaurus bucklandii*	Benson [106]
*Majungasaurus crenatissimus*	Burch and Carrano [99]
*Sanjuansaurus gordilloi*	Alcober and Martínez [89]
*Segisaurus halli*	Carrano *et al*. [105]
*Tyrannosaurus rex*	Brochu [87]
**Basal Sauropodomorpha**	
*Aardonyx celestae*	Yates *et al*. [8] and specimens referred there
*Adeopapposaurus mognai*	PVSJ 610
*Anchisaurus polyzelus*	YPM 1883/ACM 41109
*Antetonitrus ingenipes*	BP/1/4952/4956/4957/5091/5339
*Coloradisaurus brevis*	Apaldetti *et al*. [83]
*Efraasia minor*	SMNS 12354/12667/12668/12684
*Eoraptor lunensis*	PVSJ 512; Sereno *et al*. [93]
*Euskelosaurus browni*	SAM-K386
*Gyposaurus sinensis*	IVPP-V26
*Leonerasaurus taquetrensis*	MPEF-PV 1663; Pol *et al*. [127]
*Lessemsaurus sauropoides*	PVL 4822
*Lufengosaurus huenei*	IVPP-V15
*Massospondylus carinatus*	SAM-K5135; Cooper [29]
*Melanorosaurus readi*	NMQR 3314/1551
*Mussaurus patagonicus*	MLP 68-II-27-1; Otero and Pol [75]
*Panphagia protos*	PVSJ 874
*Pantydraco caducus*	NHMUK RU P24
*Plateosauravus cullinworthy*	SAM-K3345
*Plateosaurus engelhardti*	MB.R. 4404/4430; GPIT1; Huene [70]
*Ruhelia bedheimensis*	MB.R. 4718
*Sarahsaurus aurifontanalis*	TMM 43646-2/43646-3
*Saturnalia tupiniquim*	Langer *et al*. [14]
*Sefapanosaurus zastronensis*	BP/1/7424/7432/7433/7435
*Seitaad ruessi*	UMNH-VP 18040
*Yunnanosaurus huangi*	NGMJ 004546
**Sauropoda**	
*Angolatitan adamastor*	Mateus *et al*. [78]
*Apatosaurus louisae*	Gilmore [86]
*Bonitasaura salgadoi*	Gallina and Apesteguía [60]
*Camarasaurus sp*.	AMNH 462/664/823/965; FMNH 25122; Osborn and Mook [73]
*Chubutisaurus insignis*	Carballido *et al*. [49]
*Daxiatitan blinglingi*	You *et al*. [79]
*Diamantinisaurus matildae*	Poropat *et al*. [77]
*Dicraeosaurus hansemanni*	MB.R. mounted skeleton
*Elaltitan lilloi*	PVL 4628; Mannion and Otero [74]
*Euhelopus zdanskyi*	Wilson and Upchurch [59]
*Europatitan eastwoodi*	Torcida *et al*. [132]
*Giraffatitan brancai*	HMN SII; MB.R. 2249/2728
*Janenschia robusta*	MB.R. 2093.5.1
*Ligabuesaurus leanzai*	Bonaparte *et al*. [80]
*Narambuenatitan palomoi*	MAU-Pv-N-425
*Neuquensaurus australis*	MLP-CS 1050/1052/1096/1099/1169
*Opisthocoelicaudia skarzynski*	ZPAL MgD-I/25c; Borsuk-Bialynicka [15]
*Phuwiangosaurus sirindhornae*	Martin *et al*. [81]
*Rapetosaurus krausei*	FMNH-PR 2209
*Saltasaurus loricatus*	PVL 4017-101/67
*Suuwassea emiliae*	Harris [82]
*Vouivria dampariensis*	Mannion et al. [133]
*Zby atlanticus*	Mateus *et al*. [61]
**Aves**	
*Ciconia maguari*	MLP-O 14352
*Sarcoramphus papa*	MLP-O 14362
*Struthio camelus*	MLP-O 14522

Taxa showing collection numbers were first-hand studied by the author. ACM, Beneski Museum of Natural History, Amherst, Massachusetts, U.S.A.; AMNH, American Museum of Natural History, New York, New York, U.S.A.; ANS: Academy of Natural Sciences, Philadelphia, U.S.A.; AODF, Australian Age of Dinosaurs Fossil, Australia; BP, Bernard Price Institute, Johannesburg, South Africa; FMNH, Field Museum of Natural History, Chicago, Illinois, U.S.A.; GPIT, Institut und Museum für Geologie und Paläontologie, Universitat Tübingen, Tübingen, Germany; IVPP, Institute of Vertebrate Paleontology and Paleoantropology, Beijing, Peolple’s Republic of China; MAU (MRS), Museo ‘Argentino Urquiza’, Rincón de Los Sauces, Neuquén, Argentina; MACN, Museo Argentino de Ciencias Naturales ‘Bernardino Rivadavia,’ Buenos Aires, Argentina; MB, Institut für Palaontologie, Museum fur Naturkunde, Humbolt-Universität, Berlin, Germany; MCP, Museu de Ciências e Tecnologia PUCS, Porto Alegre, Brazil; MLP, Museo de La Plata, La Plata, Argentina; MPCA, Muse Provincial ‘Carlos Ameghino’, Cipolletti, Río Negro, Argentina; MPEF, Museo Paleontológico ‘Egidio Feruglio,’ Trelew, Chubut, Argentna; MNA, Museum of Northern Arizona, Flagstaff, Arizona, U.S.A.;NGMJ, Nanjing Geological Museum, Nanjing, People’s Republic of China; NHMUK, The Natural History Museum, London, U.K.; NMQR, National Museum, Bloemfontein, South Africa; PEFO, Petrified Forest National Park, AZ, U.S.A.; PMU, Palaeontological Museum, Uppsala, Sweden; PVL, Instituto ‘Miguel Lillo,’ Tucumán, Argentina; PVSJ-UNSJ, Paleontología de Vertebrados–Museo de Ciencias Naturales, Universidad Nacional de San Juan, San Juan, Argentina; SAM, Iziko–South African Museum, Cape Town, South Africa; SMNS, Staatliches Museum für Naturkunde, Stuttgart, Germany; TMM; Texas Memorial Museum, Austin, Texas, U.S.A.; UA, Université d’Antananarivo, Antananarivo, Madagascar; UCMP, Museum of Paleontology, University of California, California, U.S.A.; UMNH, Utah Museum of Natural History, Salt Lake City, Utah, U.S.A.; USNM, National Museum of Natural History, Smithsonian Institution, Washington, D.C., U.S.A.; YPM, Yale Peabody Museum, New Haven, Connecticut, U.S.A; ZPAL, Instytut Paleobiologii PAN, Warszawa, Poland.

Comparisons were also made with previous works on forelimb reconstruction in sauropodomorphs [14–16, 18, 21, 29], theropods [18, 22, 23, 30, 31], and ornithischians [32, 33]. Comparisons on muscle nomenclature among those authors are depicted in Table 3.

**Table 3:** Forelimb muscle nomenclature used in previous contributions.

Borsuk-Bialynicka [15] (Sauropoda)	Coombs [32](Ankylosauria)	Cooper [29](Basal Sauropodomorpha)	Nicholls and Russell [30](Theropoda)	Wilhite [21] (Sauropoda)	Jasinoski *et al*. [22](Theropoda)	Remes [18](Saurischia)	Langer *et al*. [14](Basal Sauropodomorpha)	Maidment and Barrett [33](Basal Ornithischia)	Burch [23, 31](Theropoda)	This contribution
-	M. trapezius	-	-	-	M. trapezius	M. cucullaris	-	-	M. trapezius	M. trapezius
M. latissimus dorsi	M. latissimus dorsi	M. latissimus dorsi	-	M. latissimus dorsi	M. latissimus dorsi	M. latissimus dorsi	M. latissimus dorsi	M. latissimus dorsi	M. latissimus dorsi	M. latissimus dorsi
M. levator scapulae	M. levator scapulae	-	-	-	M. levator scapulae	M. levator scapulae	-	-	M. levator scapulae	M. levator scapulae
-	M. rhomboideus	-	-	-	M. rhomboideus	M. rhomboideus	-	-	M. rhomboideus	M. rhomboideus
M. serratus superficialis	M. serratus ventralis superficialis	-	-	-	M. serratus ventralis thoracis	M. serratus superficialis	-	-	M. serratus superficialis	M. serratus superficialis
-	M. serratus ventralis profundus	-	-	-	M. serratus ventralis cervicis	M. serratus profundus	-	-	M. serratus profundus	M. serratus profundus
M. pectoralis	-	M. pectoralis	-	M. pectoralis	M. pectoralis	M. pectoralis	M. pectoralis	M. pectoralis	M. pectoralis	M. pectoralis
Mm. Costocoracoideus	Mm. Costocoracoideus	-	-	-	Mm. Costocoracoideus	Mm. Costocoracoideus	M. sternocoracoideus	-	Mm. Costocoracoideus	Mm. Costocoracoideus
-	-	-	-	-	-	-	M. sternocoracoideus	-	-	M. sternocoracoideus
M. scapular deltoid	M. scapular deltoid	-	M. deltoides scapularis	M. dorsalis scapulae	M. deltoideus scapularis	M. deltoideus scapularis	M. deltoideus scapularis	M. deltoideus scapularis	M. deltoideus scapularis	M. deltoideus scapularis
M. scapulohumeralis anterior	M. scapulohumeralis anterior	-	M. deltoides clavicularis	M. deltoides scapularis	M. deltoideus clavicularis	M. deltoideus clavicularis	M. deltoideus scapularis inferior	M. deltoideus clavicularis	M. deltoideus clavicularis	M. deltoideus clavicularis
-	M. teres major	-	-	M. teres major	M. teres major	M. teres major	-	-	-	M. teres major
M. subcoracoscapularis	M. subcoracoscapularis		-	-	M. subscapularis	M. subscapularis	M. subscapularis	M. subscapularis	M. subscapularis	M. subscapularis
-	-	-	-	-	M. subcoracoideus	M. subcoracoideus	-	M. subcoracoideus	M. subcoracoideus	M. subcoracoideus
-	-	-	-	-	M. scapulohumeralis anterior and posterior	M. scapulohumeralis cranialis and caudalis	M. scapulohumeralis anterior and caudalis	M. scapulohumeralis posterior	M. scapulohumeralis anterior and posterior	M. scapulohumeralis anterior and posterior
M. supracoracoideus	M. supracoracoideus	M. supracoracoideus	M. supracoracoideus pars scapularis and coracoideus	M. supracoracoideus	M. supracoracoideus	M. supracoracoideus pars scapularis and coracoideus	M. supracoracoideus	M. supracoracoideus	M. supracoracoideus	M. supracoracoideus
M. coracobrachialis brevis and longus	M. coracobrachialis	M. coracobrachialis	M. coracobrachialis brevis and longus	M. coracobrachialis	M. coracobrachialis brevis ventralis	M. coracobrachialis	M. coracobrachialis brevis and longus	M. coracobrachialis brevis	M. coracobrachialis brevis and longus	M. coracobrachialis brevis
M. Triceps scapularis	M. Triceps caput scapulate laterale externum	-	M. scapulotriceps	M. anconeus	M. triceps longus caudalis	M. triceps caput scapulare	M. triceps scapularis	M. triceps longus	M. triceps caput scapulare	M. triceps caput scapulare
-	-	-	-	-	M. triceps caput coracoideum	M. triceps caput coracoideum	M. triceps coracoideum	-	M. triceps caput coracoideum	M. triceps caput coracoideum
M. triceps humeralis	M. triceps caput humerale laterale / mediale / posticum	M. deltoideus major / M. triceps	-	-	M. triceps brachii caput mediale and laterlae	M. triceps capiti humerales	M. triceps brevis caudalis	M. triceps brevis	M. triceps brachii caput mediale and laterlae	M. triceps capiti humerales
M. biceps brachii	M. biceps brachii	M. biceps	M. biceps brachii	-	M. biceps brachii	M. biceps brachii	M. biceps brachii	M. biceps brachii	M. biceps brachii	M. biceps brachii
M. brachialis inferior	-	M. humeroradialis	M. humeroradialis	M. humeroradialis	M. humeroradialis	M. humero-radialis	M. humeroradialis	-	M. humeroradialis	M. humeroradialis
-	M. brachialis	M. brachialis	-	-	M. brachialis inferior	M. brachialis	M. brachialis	M. brachialis	M. brachialis	M. brachialis
-	-	-	-	-	-	M. supinator	M. supinator	-	M. supinator	M. supinator
-	-	-	-	-	-	M. ectepicondylo-ulnaris	M. flexor ulnaris	-	M. anconeus	M. flexor ulnaris
-	-	-	-	-	-	M. abductor radialis	-	-	M. abductor radialis	M. abductor radialis
M. extensor carpi radialis	-	-	-	-	-	M. extensor carpi radialis	M. extensor carpi radialis	-	M. extensor carpi radialis	M. extensor carpi radialis
M. extensor digitorum communis	-	-	-	-	-	M. extensor digitorum communis	M. extensor digitorum communis	-	M. extensor digitorum longus	M. extensor digitorum longus
M. flexor carpi ulnaris	-	-	-	-	-	M. flexor carpi ulnaris	M. flexor carpi ulnaris	-	M. flexor carpi ulnaris	M. flexor carpi ulnaris
M. extensor carpi ulnaris		-	-	-	-	M. extensor carpi ulnaris	M. extensor carpi ulnaris	-	M. extensor carpi ulnaris	M. extensor carpi ulnaris
M. pronator teres	-	-	-	-	-	M. pronator teres	-	-	M. pronator teres	M. pronator teres
M. interosseous	-	-	-	-	-	M. pronator quadratus	M. pronator quadratus	-	M. pronator quadratus	M. pronator quadratus
-	-	-	-	-	M. extensores metacarpi	-	-	-	M. extensores metacarpi	-
-	-	-	-	-	-	M. supinator manus	-	-	M. abductor pollicis longus	M. abductor pollicis longus
M. flexor digitorum communis	-	-	-	-	-	M. flexor digitorum longus	M. flexor digitorum longus	-	M. flexor digitorum longus	M. flexor digitorum longus
-	-	-	-	-	Mm. extensores digitorum superficiales	Mm. extensores digitorum superficiales	-	-	M. extensor longus digiti majoris	Mm. extensores digitorum superficiales
-	-	-	-	-	Mm. extensores digitorum profundus	Mm. extensores digitorum profundi	-	-	M. extensores digitorum profundus	Mm. extensores digitorum profundi
-	-	-	-	-	Mm. flexores digitorum superficiales	Mm. flexores digitorum superficiales	-	-	M. flexores digitorum superficiales	Mm. flexores digitorum superficiales
-	-	-	-	-	Mm. flexores digitorum profundus	Mm. flexores digitorum profundi	-	-	M. flexores digitorum profundus	Mm. flexores digitorum profundi

“-” means that the muscle was not reconstructed by the author.

### Methods

#### Phylogenetic inference

Traditionally, muscle inferences made on dinosaurs were based on phylogenetic closeness, typically presuming that crocodylian anatomy was plesiomorphic and retained by saurischian dinosaurs (e.g. [21, 34]). The approach to reconstruct dinosaurian musculature and other soft tissues was later methodologically standardized through similar phylogenetic approaches, (i.e. [35, 36]), which contended that the assumption of crocodylian plesiomorphy was largely inappropriate and birds should also be considered as a potentially informative source for archosaurian anatomy, with lepidosaurs (and turtles) as outgroups that should still be considered where feasible and relevant. Hence, the knowledge of appendicular musculature in living crocodiles and birds constitutes a keystone to understand the evolution of locomotion in Archosauria because of the extreme phylogenetic positions that those groups have within the clade [37–40].

As the Extant Phylogenetic Bracket (EPB) was described in previous contributions (e.g. [41, 42]), I will not dwell at length on this topic here but only summarize the main steps: 1) Identification of closest living relatives to the fossil taxon; 2) Verification of muscular homologies in extant taxa; 3) Identification of osteological correlates for each muscle on the bone; 4) Identification of osteological correlates for each muscle in the fossil taxon; 5) Identification of attachment sites for which an osteological correlate is not evident on the bone, considering origin and insertions phylogenetically inferred as plesiomorphic and its relation to other muscles; 6) Usage of Levels of Inferences for each muscle—for both origin and insertion—to quantify the speculation inherent to each reconstruction.

Non-avian dinosaurs represent a particular challenge when reconstructing forelimb musculature based on a living phylogenetic framework because of the deep functional disparities related to the different modes of locomotion existing between the extinct and the living forms (e.g. sprawling vs parasagittal; biped vs quadruped; non-flying vs flying). In relation to birds, non-avian dinosaurs have many differences in the musculoskeletal system that suggest changes in limb orientation, posture, and function related to locomotor habits and their use of the substrate [43, 44]. Unlike theropods in the bird lineage, non-avian dinosaurs and birds have in common few derived morphological features in their appendicular skeleton (e.g. parasagittal limb posture and medially directed femoral head) [45, 46]. It is in this context that osteological correlates (as defined by Witmer [41]) become the keystone to create ‘morphological bridges’ between extant and extinct taxa and (in the case of non-avian dinosaurs) to disclose morphological similarities obscured by different bauplans. Although the inference and muscle reconstruction were based on the parsimony principle in this study, extrapolatory analysis (*sensu* [35]) was also used when an osteological correlate is sufficiently evident to infer the soft tissue attribute.

**Fig 2:**
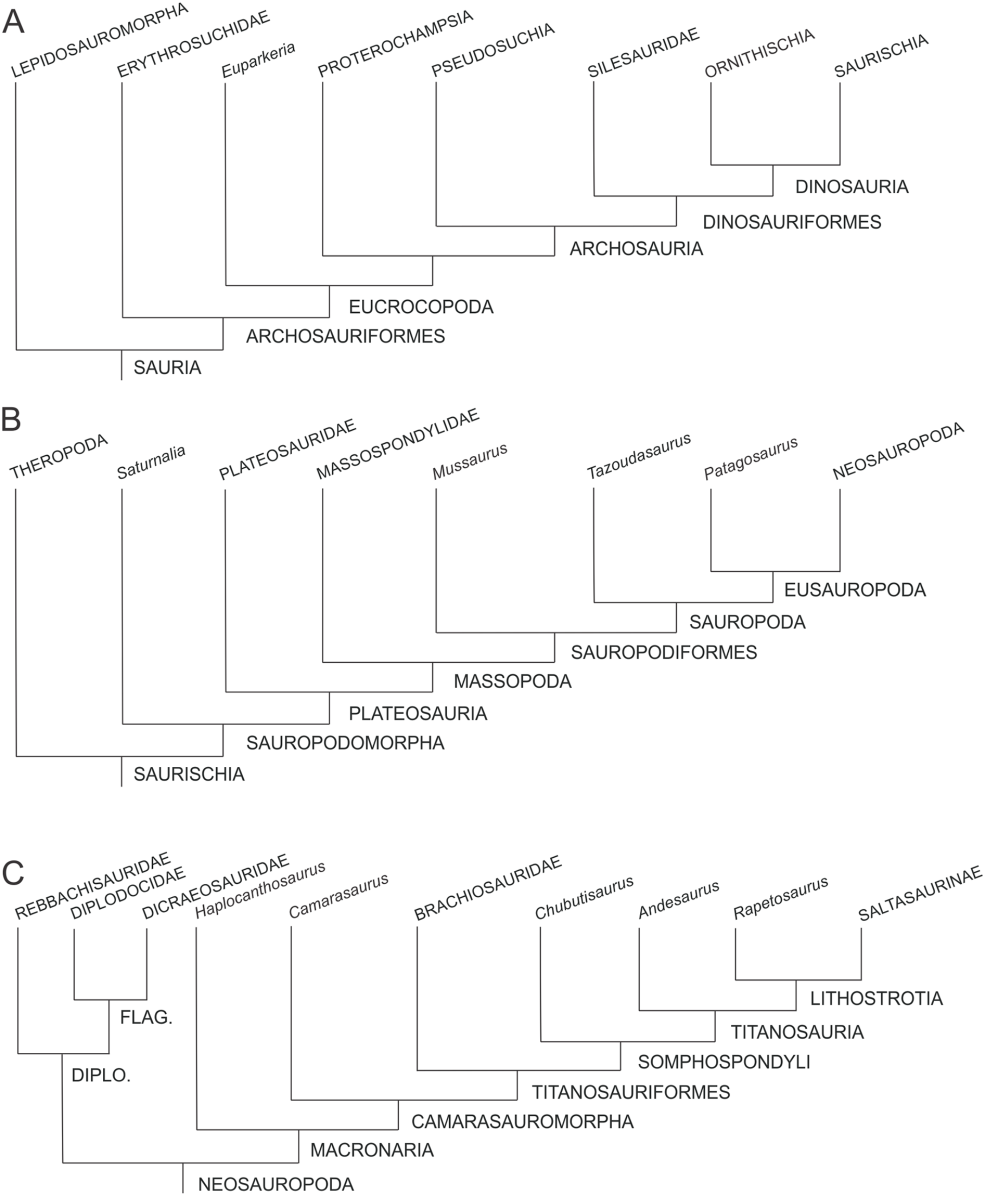
Simplified phylogenetic relationships of archosauriforms in the line to Sauropoda. Based on Ezcurra [47] (A), Otero *et al*. [48] (B) and Carballido *et al*. [49] (C).

The phylogenetic relationships of non-sauropodomorph archosauromorphs were based on Ezcurra [47], whereas the in-group relationships of Sauropodomorpha follow the schemes of Otero *et al*. [48] for non-eusauropod sauropodomorphs and Carballido *et al*. [49] for Neosauropoda (Fig 2).

#### Anatomical nomenclature, limb orientation and muscle action inferences

There is no general consensus for anatomical nomenclature as far as bone orientation is concerned. This is mainly because of the morphological disparity among tetrapods that renders standardization difficult [50, 51]. In this sense, anatomical terminology used here follows traditional or ‘Romerian’ directional terms (e.g. posterior, anterior) for skeletal structures [51].

**Fig 3:**
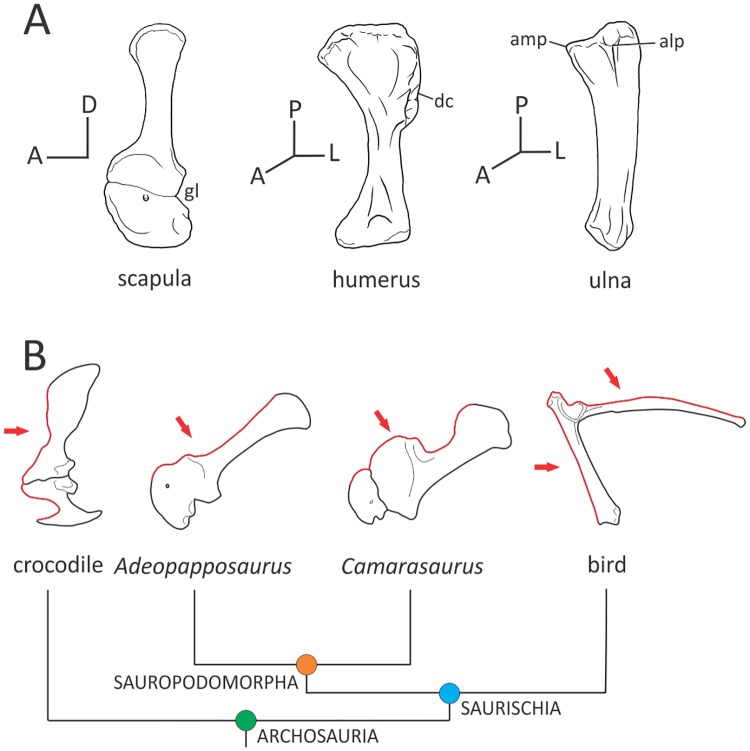
Terminology for forelimb orientation used in this study. Anatomical orientation of the forelimb of a basal sauropodomorph (A); comparative orientation of the scapulocoracoid in various archosaurs in the dinosaurian line (B). The red outline and arrow depicts the anterior surface of the bone according to the primitive vertical scapulocoracoid of living crocodiles (see the crocodile as an example). Abbreviations: A, anterior; D, dorsal; L, lateral; P, proximal. Bones in (A) based on *Plateosaurus engelhardti* (MB.R. 4404, skelett 25). In (B) crocodile silhouette taken from Meers [19], *Adeopapposaurus* based on PVSJ 610, *Camarasaurus* based on FMHN 25122, bird taken from Jenkins [128].

Regarding limb orientation, caution is needed when comparing tetrapods of sprawling gait (presumably plesiomorphic) and those of parasagittal gait (presumably derived), as each of them implies different orientations for the same bone. In this regard, primitive crocodilians developed more erect postures than living representatives [52] and living crocodilians are actually able to move through a continuum of postures which range from approximately transversal to approximately erect [53, 54]. For anatomical descriptions referred to archosaurs with non-parasagittal locomotion I followed the terminology of Reilly and Elias [55] applied to the resting pose of living crocodiles, in which the posture that best adjusts in a living crocodile is that of the transverse type. This way, the orientation of forelimb elements in living crocodiles represents the plesiomorphic configuration, whereas the condition reported in sauropods (and dinosaurs in general) represents the derived condition. Beyond the type of posture adopted by the organism (which ultimately depends on the phase of locomotion, speed, etc.) the most important thing is the relationship existing between the bones and how they are oriented in space. As a consequence, in crocodilians, the scapula adopts a vertical orientation and the humerus is laterally oriented (‘developmental orientation’ *sensu* Jasinoski *et al*. [22]; see also Baier and Gatesy [56]), whereas in sauropods the scapular blade is posterodorsally oriented and the humerus is ventrally directed [15]. In this contribution, all descriptions of the scapula (whether of a crocodile or a dinosaur) consider this bone in the primitive orientation with a vertical blade (i.e. anterior, posterior, dorsal, ventral) and disregarding the functional orientations of a posterodorsal blade (i.e. anteroventral, posteroventral, anterodorsal, posterodorsal) (Fig 3). For the humeral orientation, the anterior plane is that contained by both distal condyles. The radial surface of the ulna is considered here the anterior surface, whereas the ulnar surface of the radius is considered here the posterior surface of that bone. Regarding the autopodium, palmar surfaces are regarded as ventral. The rationale for choosing those anatomical orientations is consistency of language among different groups based on homologous bony surfaces.

Although assessing the muscle action is not the main focus of this study, I provided most probable action for each inferred muscle based on previous works by Otero *et al*. [9], Meers [19], Jasinoski *et al*. [22], Burch [23] and Allen *et al*. [57]. It is important to point out that muscles should not be considered as acting only in a single axis and are highly influenced by limb posture (e.g. [9, 58]). Hence, inferences provided here regarding muscle action should not be considered as conclusive, but only as one of the possible actions for a certain joint axis.

## Results (Figs 4–6, Table 4)

**Fig 4:**
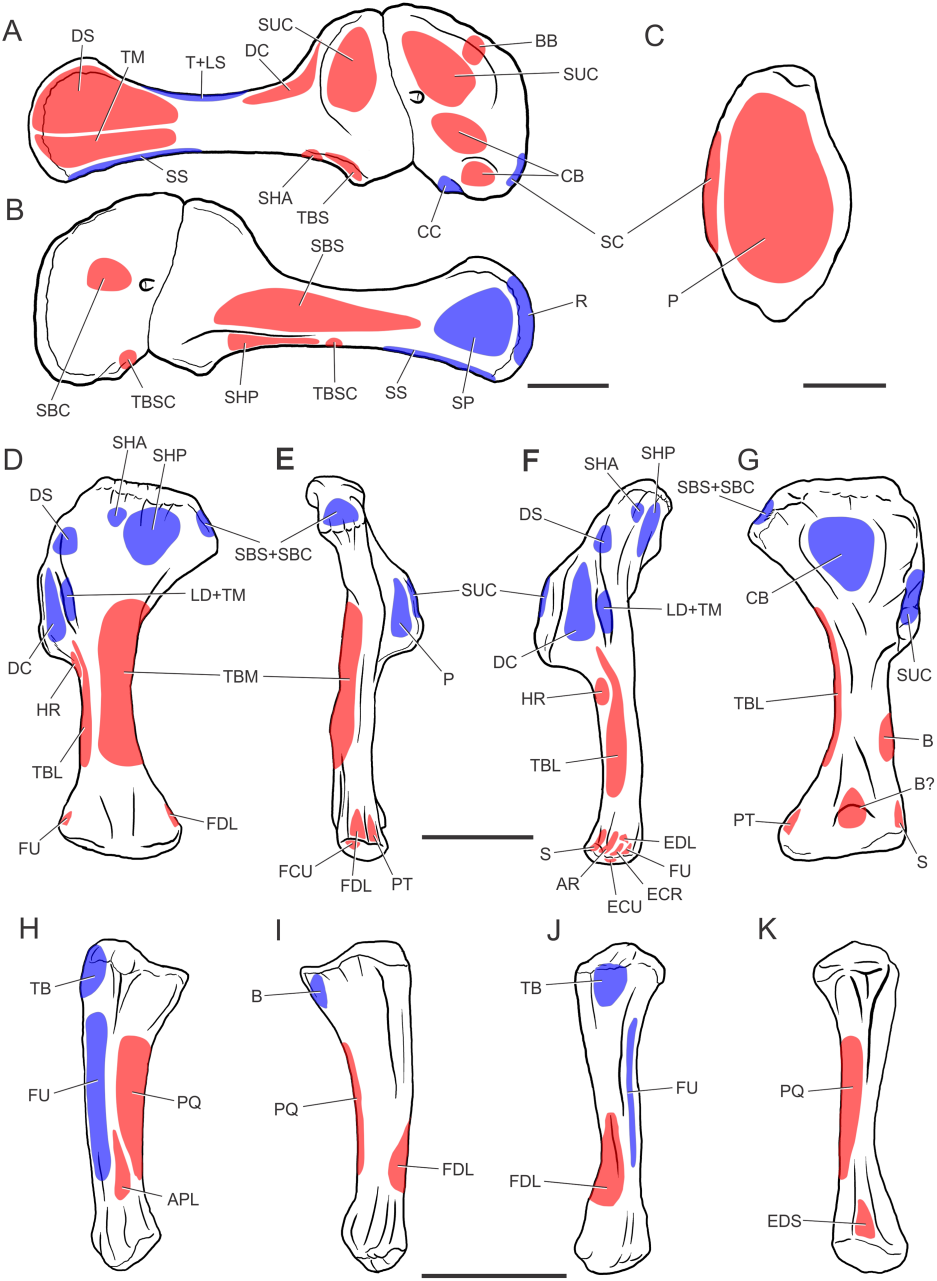
Forelimb muscles inferred for basal sauropodomorphs. Right scapula in lateral (A) and medial (B) views; right sternal plate in ventral (external) view (C); left humerus in posterior (D), medial (E), lateral (F) and anterior (G) views; right ulna in lateral (H), medial (I), posterior (J) and anterior (K) views. All bones based on *Plateosaurus engelhardti* (MB.R. 4404, skelett 25), except (C), which is based on *Adeopapposaurus mognai* (PVL 610). Abbreviations are in Table 4. Scale bar: 10 cm [except for (C) which is 3 cm].

**Fig 5:**
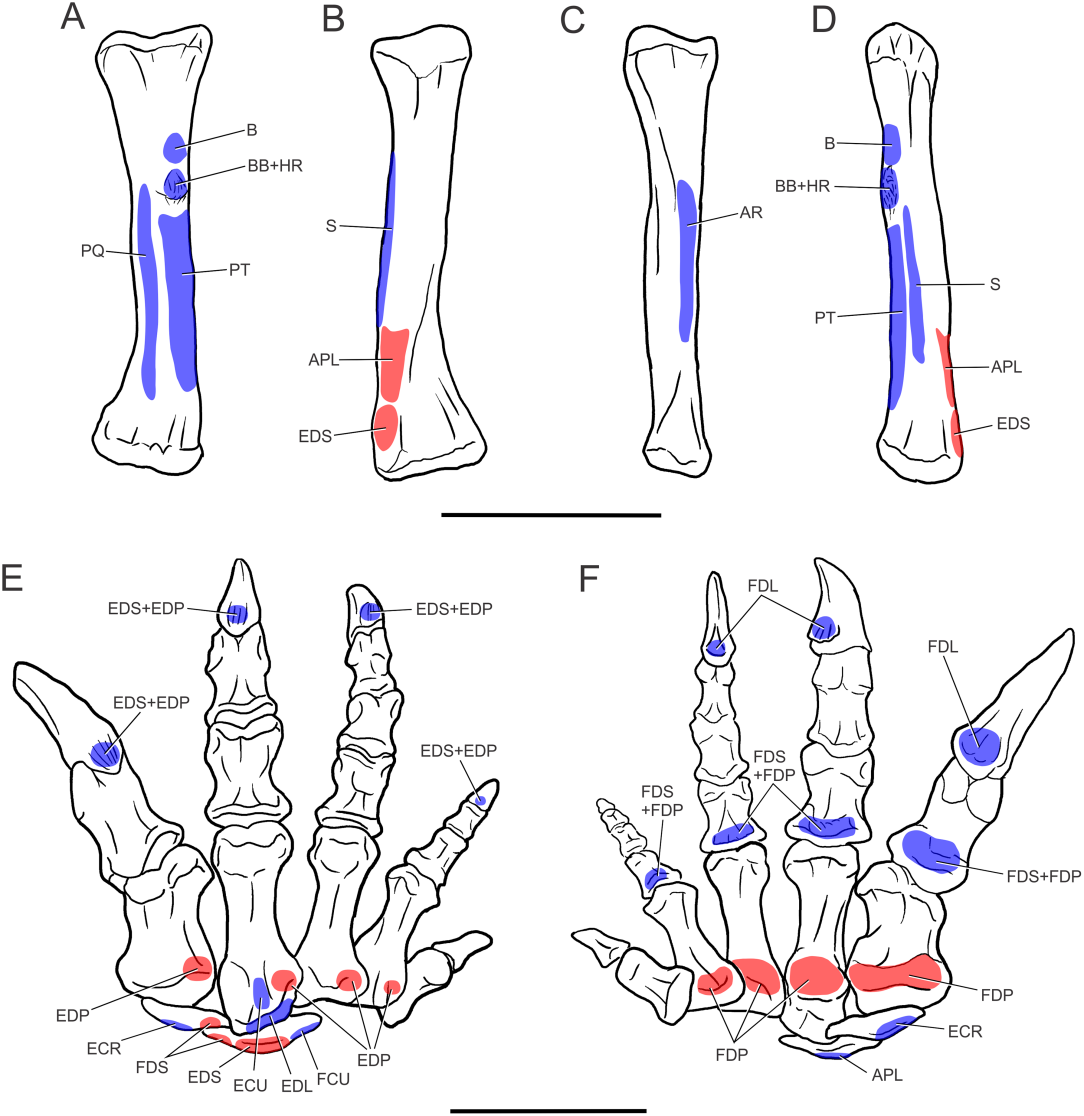
Forelimb muscles inferred for basal sauropodomorphs. Left radius in posterior (A), anterior (B), lateral (C) and medial (D) views; right manus in dorsal (E) and palmar (F) views. All bones based on *Plateosaurus engelhardti* (MB.R. 4404, skelett 25)(A)-(D) and P. *engelhardti* (MB.R. 4430, skelett C) (E), (F). Abbreviations are in Table 4. Scale bar: 10 cm.

**Fig 6:**
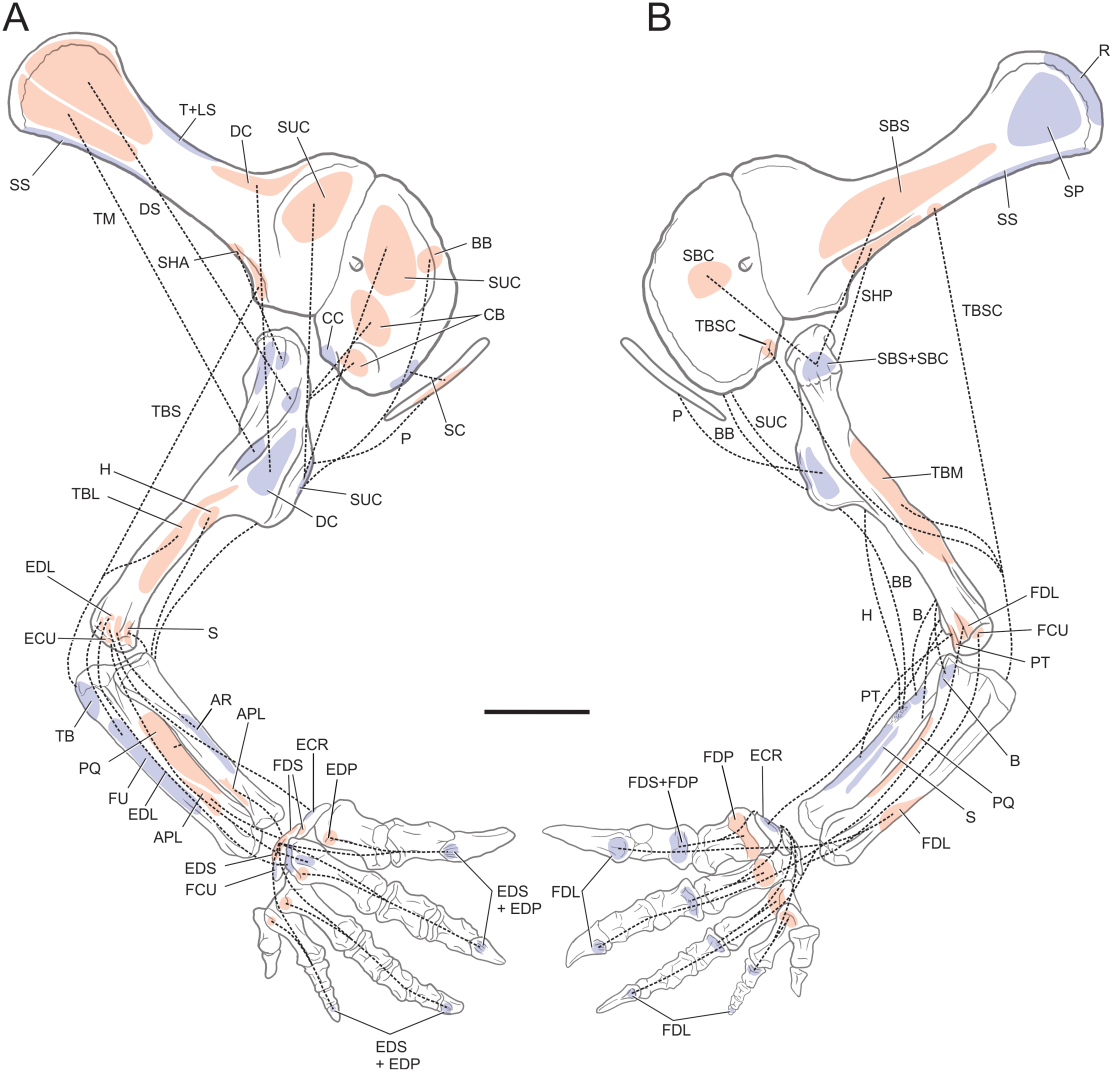
Reconstruction of the forelimb muscles for basal sauropodomorphs. Lines of action and mapped muscles on the forelimb in lateral (A) and medial (B) views. All bones based on *Plateosaurus engelhardti* (MB.R. 4404, skelett 25) and P. *engelhardti* (MB.R. 4430, skelett C), except for the sternal plate, which is based on *Adeopapposaurus mognai* (PVL 610). Scale bar: 10 cm (except for the sternal plate, which is not to scale).

**Table 4:** Shoulder and forelimb muscles inferred to be present in Sauropodomorpha, and their approximate locations.

Muscle	Abbreviation	Origin	Level of Inference	Insertion	Level of Inference
Trapezius	T	Occiput and thoracodorsal fascia (an origin from posterior cervical ribs would be Level II’).	I’	Dorsal area above the acromion process	I’
Latissimus dorsi	LD	Neural spines of posterior cervical and anterior dorsal vertebrae (a posterior head is a Level II’)	I	Ridge posteromedially placed relative to the deltopectoral crest	I
Levator scapulae	LS	Anteriormost cervical ribs	II’	Anterior margin of scapular blade	II’
Rhomboideus	R	Thoracodorsal fascia of posterior cervical neural spines (*pars caudalis* is a level II’)	I’	Dorsomedial surface of scapular blade (*pars caudalis* is a level II’)	I’
Serratus superficialis	SS	Anterior dorsal ribs	I’	Posteromedial margin of scapular blade	I’
Serratus profundus	SP	Posterior cervical vertebra and anterior dorsal ribs	I’	Medial surface of dorsomedial scapula	I’
Pectoralis	P	External surface of sternal plates	I’	Medial surface of deltopectoral crest	I’
Costocoracoideus	CC	Posterior cervical ribs	II’	Anterolateral coracoid	II’
Sternocoracoideus	SC	Sternal plates	I’	Posteroventral coracoid	I’
Deltoideus scapularis	DS	Lateral surface of the scapular blade	II’	posterior side of the humerus, close to the humeral head	II
Deltoideus clavicularis	DC	Acromial region along the anterodorsal surface of the scapula	I’	Posterior surface of the deltopectoral crest	I’
Teres major	TM	Posterolateral surface of the scapular blade, on the distal half of the blade	III’	Ridge posteromedially placed relative to the deltopectral crest	III
Subscapularis	SBS	Medial surface of the scapular blade, just above the ventromedial ridge	I’	Proximal end of the humerus, medial to the humeral head	I
Subcoracoideus	SBC	Medial side of the coracoid	I’	Proximal end of the humerus, medial to the humeral head	I
Scapulohumeralis anterior	SHA	Posterolateral margin of the scapular blade, above the scapular glenoid lip	I’	Proximoposterior surface of the humerus, below the humeral head	I’
Scapulohumeralis posterior	SHP	Posteromedial margin of the scapular blade, above the scapular glenoid lip	I’	Proximoposterior surface of the humerus, below the humeral head	I’
Supracoracoideus intermedius	SCI	Lateral scapulocoracoid boundary	I’	Distal surface of deltopectoral crest	I
Supracoracoideus brevis	SCB	Lateral coracoid	I’	Distal surface of deltopectoral crest	I
Coracobrachialis brevis ventralis	CBV	Lateral coracoid, on fossa	I’	Internal surface of the deltopectoral crest	I’
Triceps brachii caput scapulare	TBS	posterolateral surface of the glenoid rim, on scar	I	Ulnar olecranon process	I
Triceps brachii scapulocoracoideus	TBSC	Ramii on the posterior margin of scapula and coracoid	I’	Ulnar olecranon process	I
T. brachii caput lateralis	TBL	Posterolateral surface of humeral shaft	I’	Ulnar olecranon process	I
T. brachii caput medialis	TBM	Medial and distal portion of the humeral shaft	I’	Ulnar olecranon process	I
Biceps brachii	BB	Anterolateral surface of the coracoid (origin from the humerus is a Level II’)	I	Proximomedial surface of the radius (insertion on the ulna is a Level II’)	I
Humeroradialis	H	Anterolateral surface of humerus, posterior to the deltopectoral crest	II’	Tubercle of the proximal radius, on anteromedial side	II
Brachialis	B	Anteromedial surface of the humerus, distal to the deltopectoral crest, possibly on the cuboid fossa	I or I’	Proximomedial surface of the radius (insertion on the ulna is Level II’)	I’
Supinator	S	Ectepicondyle of the humerus	I	Anteromedial radial shaft	I’
Flexor ulnaris	FU	Ectepicondyle of the humerus	I	Anterolateral surface of ulna	I’
Abductor radialis	AR	Ectepicondyle of the humerus	II	Anterior surface of the radius	II’
Extensor carpi radialis	ECR	Ectepicondyle of the humerus	I	Dorsal surface of distal carpal I	I’
Extensor digitorum longus	EDL	Ectepicondyle of the humerus	I	Proximodorsal margin of metacarpal II	I’
Flexor carpi ulnaris	FCU	Entepicondyle of the humerus	I’	Distal carpus	I’
Extensor carpi ulnaris	ECU	Distal ectepicondyle	I’	Proximodorsal surface of metacarpal II	I’
Pronator teres	PT	Entepicondyle of the humerus (additional head is a Level II’)	I’	Anterior surface of the radial shaft (additional head is a Level II’)	I’
Pronator quadratus	PQ	Radial side of the ulna	I’	Ulnar side of the radius (or proximal end of metacarpal I is also a Level II’)	II’
Abductor pollicis longus	APL	Lateral shaft of the radius and ulna	I’	Proximomedial margin of metacarpal I or II	II’
Flexor digitorum longus	FDL	Entepicondyle of the humerus and posterior surface of the ulna (ulnar surface of distal carpals is a Level II’)	I’	Flexor surface of ungual of digit II (any additional digit is Level II)	I
Extensores digitorum superficialis	EDS	Distal and anterior surface of radius and ulna and probably distal carpal I	I’	Extensor process of ungual phalanx	I’
Extensores digitorum profundi	EDP	Proximal and dorsal surface of metacarpals	I’	Extensor process of ungual phalanx	I’
Flexores digitorum superficialis	FDS	Distal carpals	I	Flexor processes of proximal phalanges	
Flexores digitorum profundi	FDP	Proximoventral surface of metacarpus	I’	Flexor process of proximal phalanges	I’

Levels of inference correspond to those that are conservative in extant archosaurs (I) or varied and thus ambiguous for Archosauria (II); level III inferences (parsimoniously absent in ancestral Archosauria) were not used. Prime (I’, II’) annotations indicate attachments lacking clear osteological correlates, which can still be reconstructed but only have approximate, relative rather than more specific, direct locations (I,II).

### *M*. *trapezius* (T)

It is a broad, sheet-like muscle placed superficially and extended anteriorly over the vertebral column, involving occiput, cervical vertebrae and the scapular girdle. It can be a single muscle, as occurs in amphibians, slightly differentiated into two portions as in *Sphenodon*, or constituted by two heads, as in squamates and living archosaurs. In this case, both heads can also be named as *M*. *cucullaris* [18, 26], being *M*. *trapezius sensu stricto* the one associated with the scapular region. In crocodiles, the latter originates from the thoracodorsal fascia which covers the dorsal musculature of the cervical region, inserting fleshily (i.e. leaving no scars) dorsal to the acromion process [18, 19, 24]. Although *M*. *trapezius* was damaged after the skin was removed in the dissected specimen of *Caiman*, it still showed that the insertion of this muscle actually was shared with that of *M*. *levator scapulae* (see also Meers [19]; Suzuki and Hayashi [24]).

In birds, *M*. *trapezius* consists of three portions (i.e. *pars capitis*, *cervicis*, *clavicularis* [26]). By position, *M*. *trapezius pars cervicis* would correspond to that of other reptiles, originating from the occiput as well as from the lateral surface of posterior cervical ribs (*processus*. *costalis* [26]). The insertion of *M*. *trapezius* in birds varies, depending on the presence or loss of the furcula. If such structure is lost, as in ratites, its insertion is on the acromial area [18, 26].

Considering the ancestral presence of *M*. *trapezius* in reptiles, it is plausible to infer its presence in Sauropodomorpha, albeit with the uncertainty of its double origin because of the lack of osteological correlates. As in squamates and living archosaurs, it would have originated from the occiput and thoracodorsal fascia over the cervical vertebrae and inserted on the anterior surface of the scapular blade, just above the acromion, most probably around the same area of insertion of *M*. *levator scapulae*. An origin on posterior cervical ribs, as in birds, would imply more speculation (Level II’). Regarding its morphology, *M*. *trapezius* must have been notably elongated in basal sauropodomorphs because of the presence of ten cervical vertebrae, as was the general condition in this group [1]. In sauropods, this muscle would have been notably enlarged, considering the presence of at least 12 cervical vertebrae—and the extreme condition of 17 in *Euhelopus* [59]. Hence, the fan-shaped morphology described for living crocodiles is expected to be modified in Sauropodomorpha, consisting in an elongated cervical and sheet-like scapular portion, resembling the elongate morphology present in living birds. Both the fleshy origin and insertions of *M*. *trapezius* in living archosaurs preclude precise inference of attachment areas in sauropodomorphs.

In living crocodiles and most probably also in sauropodomorphs (see also Burch [23] for an interpretation on *Tawa*) the main action of *M*. *trapezius* is to pull dorsally and anteriorly the scapular blade, in which case protraction is aided [19, 22, 57].

### *M*. *latissimus dorsi* (LD)

**Fig 7:**
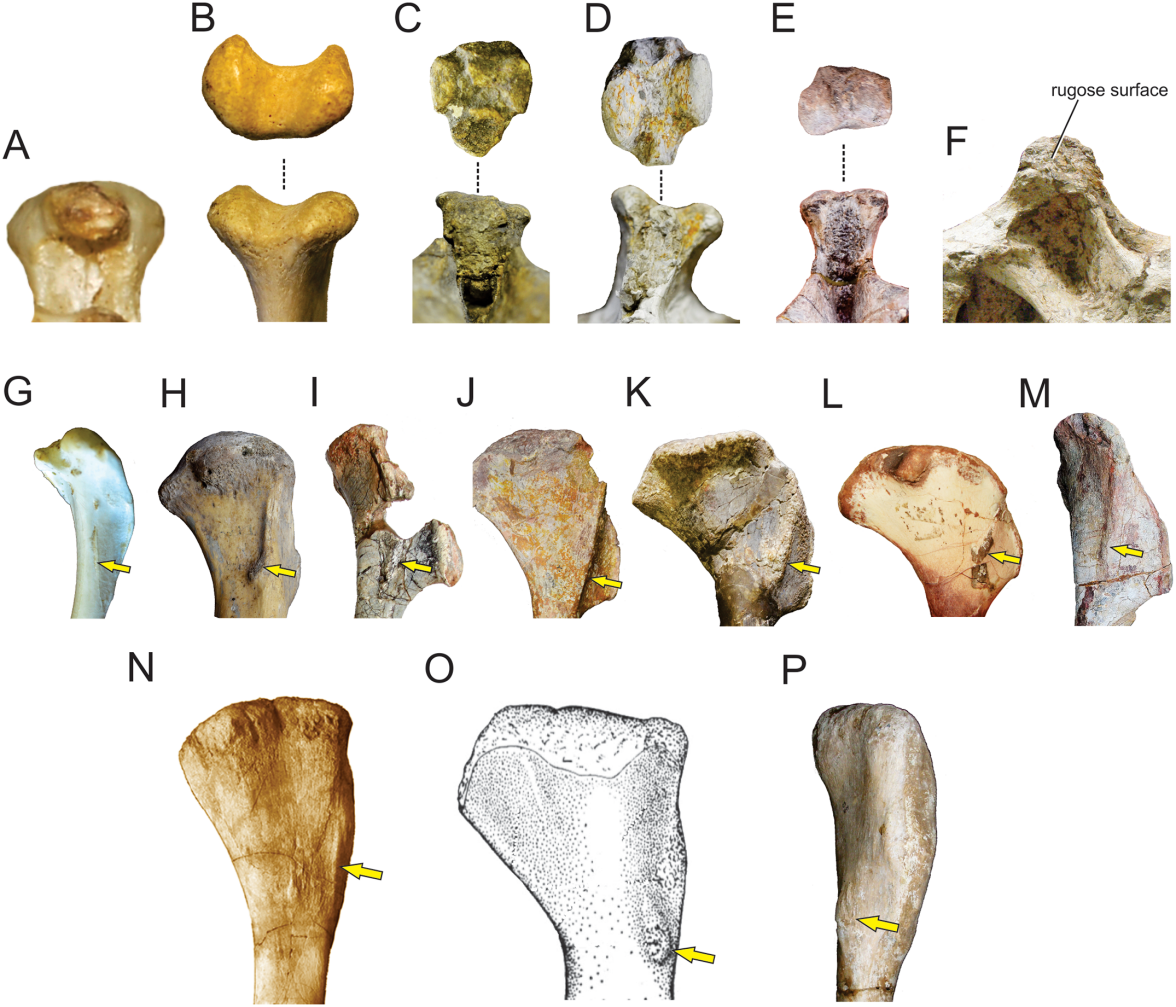
Osteological correlates of *Mm*. *latissimus dorsi* and *rhomboideus* in living archosaurs and Sauropodomorpha. Origin site of *Mm*. *latissimus dorsi* and *rhomboideus* on neural spines along the cervicodorsal transition in *Sarcoramphus papa* (MLP-O 14362) (A) in anterior view, *Caiman yacare* (B); *Plateosaurus engelhardti* (MB.R 4404–24)(C); *Ruehleia bedheimensis* (MB.R 4718–42)(D); *Plateosauravus cullinworthy* (SAM-K3345)(E) in anterior (bottom) and dorsal (top) views; and *Euhelopus zdanskyi* (PMU 233)(F) in lateral view. Insertion site of *M*. *latissimus dorsi* on the posterolateral surface of the humerus (denoted with an arrow) in *Sarcoramphus papa* (MLP-O 14362) (G), *Crocodylus niloticus* (H), *Saturnalia tupiniquim* (MCP 3845-PV)(I); *Efraasia minor* (SMNS 12354)(J); *Plateosaurus engelhardti* (MB.R 4404–44, reversed from left)(K); *Adeopapposaurus mognai* (PVSJ 610, reversed from left)(L); *Mussaurus patagonicus* (MLP 68-II-27-1)(M);; *Rapetosaurus krausei* (FMNH-PR 2209, reversed from left)(N); *Opisthocoelicaudia skarzinskii* (reprinted from Borsuk-Bialynicka, Fig 7D in [15], under a CC BY license, with permission from Instytut Paleobiologii PAN, original copyright 1977)(O); *Neuquensaurus australis* (MLP-CS 1099)(P) in posterolateral view. Not to scale.

*M*. *latissimus dorsi* is widely present among reptiles and shows varied degrees of development; it has a relatively uniform morphology in non-avian reptiles. This is a sheet-like, fan-shaped muscle covering the dorsal surface of neural spines of posterior cervicals and anterior-middle dorsal vertebrae. Posteriorly it becomes narrower and inserts on the humerus. *Sphenodon* presents the most developed configuration, which is subsequently reduced in squamates [17, 18]; in crocodiles it originates on the fascia of the neural spines of the last cervicals and first four dorsals. In this region, the tips of the neural spines are expanded. It inserts, together with *M*. *teres major* (when present) onto a rugosity placed on the proximolateral surface of the humerus behind the deltopectoral crest (Fig 7). In Neornithes, on the other hand, *M*. *latissimus dorsi* is divided into an anterior and a posterior portion, both clearly visible on the dorsal surface of the back, after removing the skin. *M*. *latissimus dorsi cranialis* originates from the neural spines of the last cervicals and the anterior dorsals, in which neural spines are mediolaterally thicker (as in crocodiles); it inserts onto the posterior surface of the humerus, on the proximal third, where it leaves a longitudinal scar (*Gallus*, *Sarcoramphus*) (Fig 7). *M*. *latissimus dorsi caudalis* in *Gallus gallus* is separated from the anterior head and originates in the last thoracic vertebrae and may include part of the synsacrum. Its insertion may be (depending on the group) both fleshily and adjacent to the anterior head or via aponeurosis together with the scapular head of *M*. *triceps* [51]. A third head (*pars metapatagialis*) is also reported [18, 30].

In crocodiles, *M*. *latissimus dorsi* acts to abduct and extend the humerus [19, 57]. In sauropodomorphs, a preponderant extensor action is expectable since the anatomical position of the scapular blade is inclined about 60° (and not vertical as in crocodiles), which produces a posteroventral orientation of the glenohumeral joint, making retraction much more plausible than abduction for this muscle, as also reported for other biped saurischians [22, 23].

In sauropodomorphs, *M*. *latissimus dorsi cranialis* is the portion that most likely was present, as it is reported in both bracket taxa. One possible osteological correlate for the origin of this muscle in basal sauropodomorphs is the transversely expanded dorsal tip of neural spines among the cervicodorsal transition. This feature is present in examined specimens of crocodiles (*Caiman*, *Crocodylus*) and birds (*Struthio*, *Sarcoramphus*) and is also widely distributed among basal sauropodomorphs such as *Ruehleia bedheimensis* (MB.R 4718–42), *Plateosaurus engelhardti* (MB.R 4404–24), *Plateosauravus cullinworthy* (SAM-K3345), and *Melanorosaurus readi* (NMQR 3314), and also in the titanosaur sauropod *Bonitasaura salgadoi* (Fig 4 in [60]) (Fig 7). In other sauropods there is an increase in striation of the tips of neural spines through posterior cervical vertebrae (e.g. *Euhelopus zdanskyi*, Fig 11 in [59]; *Rapetosaurus krausei* FMNH-PR 2209). Apart from these particular features, a common trend in all sauropodomorphs is the anteroposterior shortening and dorsoventral expansion of neural spines through the cervicodorsal transition, which could be linked to the presence of *M*. *latissimus dorsi*. Its insertion most probably was onto a well-developed proximodistally oriented ridge placed just medial to the deltopectoral crest on the posterior surface of the humerus; thus, it corresponds to a Level I of Inference. This osteological correlate is also widely present among basal sauropodomorphs (e.g. *Saturnalia tupiniquim* [14]; *Efraasia minor* SMNS 12354; *Plateosaurus engelhardti* MB.R 4404–44; *Adeopapposaurus mognai* PVSJ 610; *Massospondylus carinatus* SAM-K5135; *Yunnanosaurus huangi* NGMJ 004546; *Mussaurus patagonicus* MLP 68-II-27-1) (Fig 7). Among sauropods, a similar ridge is present in the turiasaur *Zby atlanticus* (Fig 8F in [61], ‘posterolateral bulge’), whereas Borsuk-Bialynicka [15] placed the insertion of *M*. *latissimus dorsi* on a rounded, dome-like scar lateral to the deltopectoral crest, most probably sharing insertion with *M*. *teres major*. This scar is also present in the titanosaurs *Rapetosaurus* (FMNH-PR-2209) and *Neuquensaurus australis* (MLP-CS 1099). The presence of a posterior head of *M*. *latissimus dorsi* among sauropodomorphs is equivocal, as it is not present in crocodiles, corresponding to a Level II’ inference.

### *M*. *levator scapulae* (LS)

*M*. *levator scapulae* is a broad, sheet-like muscle running from the cervical vertebrae to the scapular blade. It is anteroposteriorly oriented and covers the lateral side of the neck, lying below *M*. *trapezius* and above the *longissimus* system. *M*. *levator scapulae* is present in most reptiles, except turtles and birds [18, 26]. In lepidosaurs it presents a dorsal and a ventral portion, whereas in crocodiles this condition is only reported in some forms [18, 62]. In *Caiman* and crocodiles in general it originates fleshily from the posterolateral surface of the anteriormost cervical ribs (probably the first), also taking part of the diapophyses, and inserts fleshily along the anterior margin of the scapular blade.

The absence of *M*. *levator scapulae* in turtles and birds could be the result of particular specializations in each group. In the case of turtles, the loss of *M*. *levator scapulae* may have been a consequence of the fixation of the scapula to the dorsal and dermal bones, rendering the elevation action of the muscle obsolete. In the case of birds, its loss must have been related to the reorientation of the scapula to the nearly horizontal plane.

**Fig 8:**
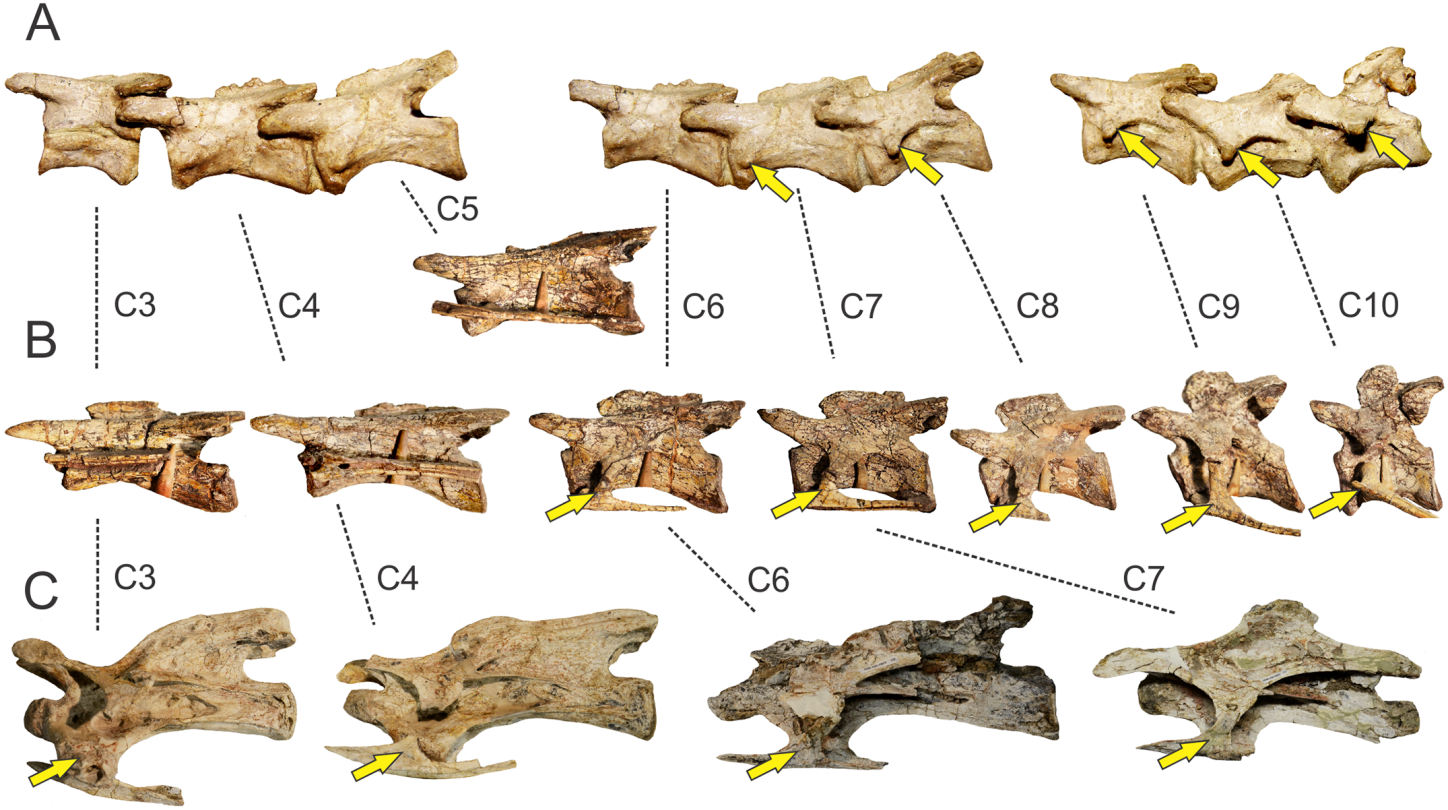
Osteological correlates of *Mm*. *levator scapulae* and *serratus profundus* in Sauropodomorpha. Cervical vertebrae showing putative origin site of *Mm*. *levator scapulae* (if present) and *serratus profundus* in *Pantydraco caducus* (NHMUK RU P24)(A); *Plateosaurus engelhardti* (GPIT1)(B); *Erketu ellisoni* (IGM 100/1803; C3 and C4 reversed from right)(C). The arrow denotes the lateral surface of the diapophysis-rib complex where *Mm*. *levator scapulae* and *serratus profundus* would have been attached. Not to scale.

Although the presence of *M*. *levator scapulae* in sauropodomorphs is equivocal, its primitive presence in lepidosaurs and crocodiles means that it can be reconstructed using a Level II’ inference. If present in sauropodomorphs, *M*. *levator scapulae* may have a different extent when compared to basal forms and sauropods. Basal sauropodomorphs present weak diapophyses and slender ribs on the anterior cervical vertebrae, precluding an origin of the muscle in the anteriormost portion of the neck. In contrast, sauropods display a well-developed diapophysis-rib complex along most of their neck (Fig 8), favouring a stout anchorage for this muscle, especially considering the extreme neck elongation present in this group, which most probably would have needed a more extensive origin site to cover the functional requirements of *M*. *levator scapulae* (see Discussion). As in lepidosaurs and crocodiles it would have been inserted on the anterior margin of the scapular blade, sharing its attachment with *M*. *trapezius*.

### *M*. *rhomboideus* (R)

With no homologue among non-archosaur reptiles, *M*. *rhomboideus* is a narrow muscle deep beneath *M*. *trapezius* and running from ‘thoracic’ vertebrae to the dorsalmost end of the scapula. It is composed by a single head in *Caiman*, originating on the fascia covering the *M*. *longissimus dorsi* (over the neural spines) and inserting on the suprascapular cartilage, although it may also cover some part of the osseous scapular blade, leaving no osteological correlates (see also Remes [18]; Suzuki and Hayashi [24]).

In Neornithes there are superficial and a deep heads, the former of which corresponds to the crocodilian *M*. *rhomboideus*. Both portions originate (sometimes aponeurotically, [18, 22]) from the neural spines of posterior cervical (Fig 7) and thoracic vertebrae, and also the anterior end of the pelvis, inserting fleshily on the dorsomedial portion of the scapula [27].

The presence of the anterior head of *M*. *rhomboideus* in sauropodomorphs can be inferred with some speculation of its fleshy attachments (Level I’). The presence of a posterior head is equivocal (Level II’), probably corresponding to a novelty of the bird lineage [18, 22] or even an early theropod novelty [23]; hence, it is not reconstructed as present in the group studied herein. As in living archosaurs, the origin sites of *M*. *rhomboideus* in sauropodomorphs are the posterior cervical neural spines. As described above, such structures increase surface and striations posteriorly on the cervical series, and such shifting is most probably related to the cervical musculature anchored in the pectoral girdle, like *Mm*. *latissimus dorsi* and *rhomboideus* (see also Schwarz *et al*. [16]). Although there is no evidence of a suprascapular cartilage among sauropodomorphs (but see Schwarz *et al*. [16]), the scapular blade of basal sauropodomorphs is long enough to include extensive attachment of *M*. *rhomboideus*.

The action of *M*. *rhomboideus* in crocodiles would be to adduct the forelimb [57]. The posterodorsal orientation of the scapular blade in sauropodomorphs would have shifted the function of *M*. *rhomboideus*, acting as a limb flexor (protractor; see also Jasinoski *et al*. [22]; Burch [23]).

### *M*. *serratus superficialis* (SS)

*M*. *serratus superficialis* is conservatively present in reptiles, running from the ribcage, in a deep position, to the scapular blade. In both lepidosaurs and crocodiles, this muscle has its fleshy origin on the posterior cervical ribs (or the last one, as in *Sphenodon*), and anteriormost sternal ribs [17, 18]. In a dissected specimen of *Caiman*, however, its origin is restricted only to posteriormost cervical ribs. Its insertion covers the anteromedial surface of the scapular blade, including part of the suprascapular cartilage.

In Neornithes, *M*. *serratus superficialis* consists of several fleshy bundles (*pars cranialis*, *caudalis* and *metapatagialis*, *sensu* Vanden Berge and Zweers [26] and McKitrick [27]), the origins of which involve more structures than in lepidosaurs and crocodiles and include part of the posterior cervical vertebrae and maybe also the thoracic ribs. It inserts on the medial surface of the scapula, deep to the insertion of *M*. *rhomboideus profundus*.

The presence of *M*. *serratus superficialis* in sauropodomorphs is unequivocal, although the precise origin and number of heads is somewhat speculative since neither scars nor ridges are reported for this muscle. No uncinate processes are present in this group; hence its origin may have been restricted to anteriormost dorsal ribs, maybe including the posteriormost cervical ribs, as in living archosaurs. In general, the medial surface of the scapular blade of sauropodomorphs does not presents any trace of scars for insertion of *M*. *serratus superficialis*, which resembles the condition present in living crocodilians. However, Borsuk-Bialynicka [15] reported a set of tuberosities on the posterior edge of the scapular blade, attributing them to the insertion of *M*. *serratus superficialis*.

The action of this muscle in sauropodomorphs could have been scapular extension and also the elevation of the neck when the forelimb was fixed.

### *M*. *serratus profundus* (SP)

Like its superficial homonym, *M*. *serratus profundus* covers the lateral sides of the ribcage, lying deep beneath *M*. *latissimus dorsi* and inserting onto the medial side of the scapular blade. In lepidosaurs it originates from several cervical ribs, whereas in crocodiles its origin only involves the posteriormost cervicals, but also anterior thoracic ribs [18]: it includes the transverse processes (*Caiman*) and leaves no scars. The condition in Neornithes is very similar, originating also on the uncinated process (*Gallus*). The fascicles of this muscle are clearly visible after removing *M*. *trapezius* and *M*. *latissimus dorsi*. In both cases it inserts fleshily on the medial surface of the suprascapular cartilage.

As with *M*. *serratus superficialis*, the presence of *M*. *serratus profundus* in sauropodomorphs and in dinosaurs in general is unequivocal [14–16, 22, 23, 33]. However, the lack of scars or striations precludes allocating it a specific origin area. As in crocodiles, *M*. *serratus profundus* in basal sauropodomorphs most probably originates on middle cervical vertebrae (fifth or sixth), in which the diapophyses become broader, increasing their surface to host the origin site of this muscle. This shifting in diapophysis size is trackable in complete cervical series of *Pantydraco caducus* (NHMUK RU P24), *Ruehleia bedheimensis* (MB.R. 47189), *Plateosaurus engelhardti* (MB.R. 4404 skelett 25), *Adeopapposaurus mognai* (PVSJ 610), *Sarahsaurus aurifontanalis* (TMM 43646–2) and *Melanorosaurus readi* (NMQR 3314). In sauropods, however, cervical diapophyses are well-developed from anteriormost elements [63], suggesting a more anterior and extensive origin site of *M*. *serratus profundus* (Fig 8). No osteological correlates are present in the dorsomedial side of the scapula among sauropodomorphs, suggesting a fleshy insertion as in living archosaurs.

*M*. *serratus profundus* would have flexed the scapula on the one hand, but its broad attachment to the ribcage would also have helped to stabilize the scapular girdle and it may have had some influence on respiration.

### *M*. *pectoralis* (P)

*M*. *pectoralis* is the largest and most massive muscle of the scapular girdle and forelimb, with a relatively constant morphology among reptiles and covering pectoral musculature. In both lepidosaurs and crocodiles the sheet-like *M*. *pectoralis* originates along the chest midline, including anteriormost sternal ribs, but also in the clavicles (*Sphenodon*, [18]), interclavicles and xiphisternum (squamates, [17]) via a fleshy origin [22]. In crocodiles it consists of two portions (anterior and posterior), both inserting via tendon onto the medial surface of the deltopectoral crest of the humerus, sometimes leaving scars ([22], own observations).

In Neornithes, *M*. *pectoralis* is highly modified, thicker than in crocodiles and is also the most important muscle during flight. It consists of three portions: *thoracic*, *propatagialis*, and *abdominalis*, the former of which corresponds, by position, with the posterior portion of crocodiles.

In sauropodomorphs the presence of *M*. *pectoralis* is unequivocal, although subdivision of this muscle requires some speculation since the elements from which it originated have not been preserved or were absent. Although it is not possible to identify discrete portions for *M*. *pectoralis* in non-avian dinosaurs it should be noted that sternal plates have been widely reported among Sauropodomorpha (e.g. [1, 15, 64, 65, 66–68]. These are the only bony elements of the sternal region preserved in sauropodomorphs and the possibility of associated cartilaginous elements should not be ruled out [15, 65, 69]. Additionally, gastralia are also present among some basal sauropodomorphs (*Eoraptor lunensis* PVSJ 512; *Plateosaurus* [70]; *Seitaad ruesi* UMNH-VP 18040). This suggests that possibly a posterior subdivision of *M*. *pectoralis* may have been present in this group. If present, clavicles would have been an extra point of origin for *M*. *pectoralis*, at least among basal sauropodomorphs. These structures were previously reported in this group and were inferred to be placed anterior to the coracoids, in a topological place similar to that of crocodilian interclavicles [66, 71]. The medial surface of the deltopectoral crest has no signs of rugosities or striae among sauropodomorphs, corresponding to a fleshy attachment as in living archosaurs.

The action of *M*. *pectoralis* has remained constant among reptiles, with no expected changes in dinosaurs, being mainly an adductor with some humeral flexor component [19, 23, 57]. Additionally, some medial long axis rotation of the humerus is also expectable for this muscle.

### *M*. *costocoracoideus* (C) and *M*. *sternocoracoideus* (SC)

*Mm*. *costocoracoideus* and *sternocoracoideus* are small muscles running from the ventral thoracic region to the coracoid, beneath *M*. *pectoralis*. Both can be found in lepidosaurs (*contra* Jasinoski *et al*. [22]) but only one of them is present in crocodiles and birds; hence, they are described together. In spite of this, homology of these muscles among reptiles is controversial [17–19, 23, 27].

In lepidosaurs (including *Sphenodon*), *M*. *costocoracoideus* originates on the first sternal rib and inserts on the medial surface of the scapula close to the glenoid [18, 72]. In crocodiles, only *M*. *costocoracoideus* is present; it comprises two heads and has fleshy attachments. The most superficial one originates on the posterior cervical region (cervical ribs), whereas the deepest one does so from the anterior margin of the first sternal rib [18] or even the anteriormost free ribs [19]. Other authors, however, consider a common origin for both heads on the anteriormost sternal and gastral ribs, only being differentiated at their insertion [22]. Both portions insert directly on the posterior margin of the coracoid.

In Neornithes the muscle that occupies an equivalent position to the crocodilian *M*. *costocoracoideus* is the *M*. *sternocoracoideus*, although there is no clear homology between them (but see Jasinoski *et al*. [22]). It is usually divided into a superficial and a deep portion both originating from the sternum and/or sternal ribs and inserting on the lateral process of the coracoid (superficial portion) or medial side of the same bone (deep portion).

The presence of *Mm*. *costocoracoideus* in sauropodomorphs is equivocal, corresponding to a Level II’ of Inference and no subdivision can be recognized. Nonetheless, as *M*. *costocoracoideus* is primitively present in lepidosaurs and crocodiles, it may have been retained in non-avian dinosaurs. It would have originated on the posterior cervical ribs, as there is no record of sternal ribs among Sauropodomorpha, whereas it would have inserted on the surface between the glenoid and the ventral margin of the coracoid which, in basal sauropodomorphs, corresponds to the concave and wide surface dorsal to the coracoid tubercle. The presence of *M*. *sternocoracoideus*, on the contrary, is considered here as a Level I’ of Inference, considering its primitive presence in lepidosaurs and assuming an independent loss in the crocodilian lineage, optimizing at the base of dinosaurian node as a decisive positive assessment. *M*. *sternocoracoideus* would have originated on the sternal plates and inserted on the posteroventral coracoid.

Most authors agree on a shoulder extension action for both *Mm*. *costocoracoideus* and *sternocoracoideus* [19, 57].

### *M*. *deltoideus scapularis* (DS)

This is a quite conservative muscle among tetrapods (although with some subdivisions in birds), being one of the thickest muscles of the shoulder; it originates on the scapular blade and inserts on the proximolateral surface of the humerus. In lepidosaurs it can also include part of the clavicles and the suprascapula [17]. In *Caiman*, the fleshy origin of *M*. *deltoideus scapularis* occupies the anterior portion of the scapular blade, including part of the suprascapula. In other crocodiles the origin may be also more laterally placed on the scapular blade [19]. In the analysed specimens, such area is bounded by a faint ridge running along the median line of the blade, defining an anterior region and a posterior one. Then it becomes thinner, forming a tendon inserting on the proximolateral surface of the humerus, dorsolateral to the deltopectoral crest and below the humeral head—in an extensive rugose area onto which *M*. *coracobrachialis brevis dorsalis* is also inserted—and dorsally to *M*. *deltoideus clavicularis*.

The ‘deltoid’ muscle in birds has three main subdivisions which will be described in the context of the *M*. *deltoideus clavicularis*.

Sauropodomorphs show no sign of ridge or striae on the lateral surface of the scapular blade, precluding the recognition of discrete origin site for *M*. *deltoideus scapularis*—assuming a fleshy origin for it as in crocodiles. In spite of this, this muscle would have originated following a pattern similar to that of living crocodiles, but the possibility of an extensive origin in sauropodomorphs inferred from the relatively long scapular blade is not discarded. Regarding the insertion, it is possible that *M*. *deltoideus scapularis* may have inserted on the proximalmost end of the thick ridge running proximodistally and lateral to the deltopectoral crest, ridge that also serves as the anchorage for *M*. *latissimus dorsi*.

The action of *M*. *deltoideus scapularis* would have been—as in crocodiles—abductor and also including some humeral supination and flexion [9].

### *M*. *deltoideus clavicularis* (DC)

*M*. *deltoideus clavicularis* is another muscle constituting the fleshy shoulder; it also runs from the scapula to the proximal humerus, lying anterior to *M*. *deltoideus scapularis*. It is present in all reptiles. In lepidosaurs it originates on the clavicles and sometimes the interclavicles as well [17], whereas in crocodiles (where clavicles are lost) its fleshy origin is on the acromial surface and also part of the anterolateral margin of the blade. In all cases, it inserts also fleshily onto the lateral surface of the deltopectoral crest, just below the *M*. *deltoideus scapularis* and occupying a much broader surface than the latter.

In *Gallus*, *M*. *deltoideus major*, *minor*, and *propatagialis* originate in the clavicle, furcula, and the area surrounding the acromion and coracoid contact, respectively, also with fleshy origin and insertions [18, 22]. The insertion of the *M*. *propatagialis* is on the carpus, although it can also originate directly on the deltopectoral crest [23]. The insertion of *M*. *deltoideus major* aponeurotically covers the shaft of the humerus, whereas *M*. *deltoideus minor* inserts on the posterolateral face of the deltopectoral crest of the humerus, close to the proximal end. Of the three avian subdivisions of *Mm*. *deltoideus*, *pars propatagialis* is regarded as corresponding to *M*. *deltoideus clavicularis* of other reptiles [22, 23]. Nonetheless, homology between any of the avian portions and the single head of reptiles is considered as inconclusive in this contribution.

**Fig 9:**
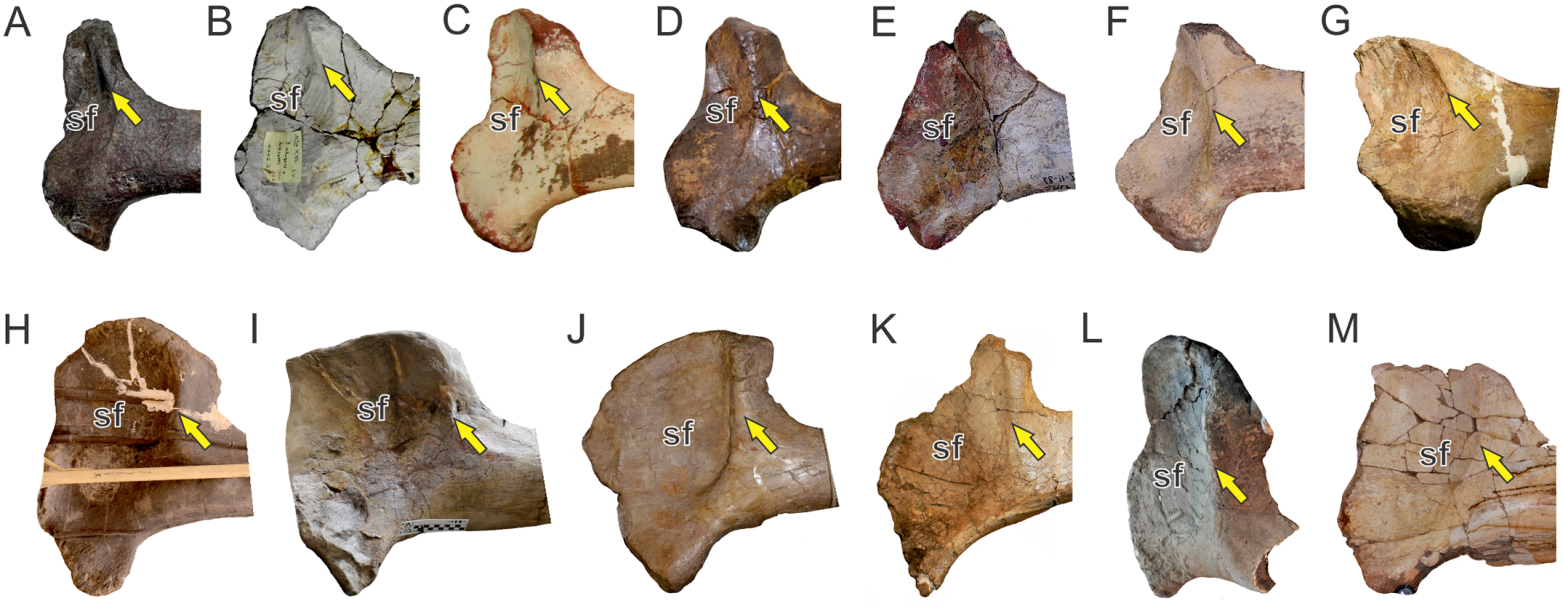
Osteological correlates on the proximal scapula in Sauropodomorpha. Proximal scapula in lateral view showing the scapular fossa (sf) and the acromial ridge (arrow) in *Panphagia protos* (PVSJ 874)(A); *Ruehleia bedheimensis* (MB.R 4718–100)(B); *Adeopapposaurus mognai* (PVSJ 610)(C); *Lufengosaurus huenei* (IVPP-V15)(D); *Mussaurus patagonicus* (MLP 68-II-27-1)(E); *Melanorosaurus readi* (NMQR 1551)(F); *Antetonitrus ingenipes* (BP/1/4952)(G); *Camarasaurus* sp. (FMNH 25122)(H); *Dicraeosaurus hansemanni* (MB.R. mounted skeleton)(I); *Giraffatitan brancai* (HMN SII mounted skeleton)(J); *Rapetosaurus krausei* (FMNH-PR 2209)(K); *Elaltitan lilloi* (PVL 4628)(L); *Opisthocoelicaudia skarzynskii* (ZPAL MgD/I-48)(M). (B), (E), (F), (I), (K) and (M) reversed from right. Not to scale.

There is no clear osteological correlate for the origin of *M*. *deltoideus clavicularis* among Sauropodomorpha, as occurs in living archosaurs. At least unequivocal is the partial origin on the dorsal surface of the acromion process, following the pattern present in living archosaurs. This surface is ventrally framed by the acromial ridge, which runs from the tip of the acromion process to the proximal end of the scapular blade. Such ridge is almost non-existent in most basal sauropodomorphs, mostly reduced to fine striae (e.g. *Panphagia protos* PVSJ 874; *Efraasia* SMNS 12684; *Adeopapposaurus* PVSJ 610; *Mussaurus* MLP 68-II-27-1) but become notably thick in *Ruehleia* (MB.R. 4718), *Lufengosaurus* (IVPP-V15), *Antetonitrus* (BP/1/4952) and more derived sauropodomorphs (e.g. *Camarasaurus* sp. FMNH 25122, Fig 75 in [73]; *Rapetosaurus* FMNH-PR 2209; *Elaltitan lilloi* PVL 4628, Fig 6A, B in [74]) (Fig 9). Although an origin on the clavicle was reported in lepidosaurs [72] and birds [23], the presence of clavicles in basal sauropodomorphs was only reported in *Massospondylus*, *Plateosaurus* [71] and *Adeopapposaurus* [66] and few reports of them exist among sauropods [65]. The unequivocal insertion of this muscle is on the lateral surface of the deltopectoral crest, on its posterior aspect and lateral to the insertion of *M*. *latissimus dorsi*, but this insertion lacks osteological correlates.

The anterior placement of this muscle on the shoulder supposes a flexor action, as in living crocodiles [9, 19, 57]. Additionally, the lateral attachment on the girdle and humerus would have favoured some abduction as well [23] and also supination [9].

### *M*. *teres major* (TM)

*M*. *teres major* is scantily distributed among tetrapods, being absent in amphibians, most lepidosaurs and birds. As other muscles of the shoulder, it runs from the lateral scapula to the proximal humerus. In crocodiles this muscle raises fleshily from the ventral end of the lateral surface of the scapular blade, just below the *M*. *deltoideus scapularis* [19, 18, 24], inserting laterally to the deltopectoral crest on the same longitudinal crest as *M*. *latissimus dorsi*.

Parsimony allows a decisive negative inference of *M*. *teres major* in sauropodomorphs since the sister group birds lack it and only one outgroup (Crocodilia) presents it. Hence, acquisition of *M*. *teres major* in the crocodilian line is the hypothesis implying less evolutionary transformations. Nonetheless, as the osteological correlate of the insertion on the humerus is quite developed not only in sauropodomorphs, but in archosaurs in general (see Discussion), I will assume, by means of extrapolation, the presence of *M*. *teres major* in the dinosaurian line as well, losing it in the bird line. Thus, as in crocodiles, its origin in sauropodomorphs would have been fleshy, whereas the insertion is on the longitudinal ridge present on the lateral aspect of the deltopectoral crest, together with *M*. *latissimus dorsi* (Fig 7). As in living crocodiles, *M*. *teres major* would have abducted the forelimb [19, 57], also including some extension (see also Wilhite [21]) and supination [9].

### *Mm*. *subcoracoscapulares* (SCS)

Plesiomorphically, this muscle has two heads. This is the condition in most diapsids, extending from the scapula and coracoid to the proximal humerus. In lepidosaurs, *Mm*. *subcoracoscapulares* originate on the medial side of the coracoid (*M*. *subcoracoideus*) and ventral part of the scapular blade (*M*. *subscapularis*) and usually part of the suprascapular cartilage. Both portions fuse to insert in the medial tuberosity of the humerus [18]. In crocodiles, which have only one head, it originates on the medial surface of the scapular blade (keeping the name of *M*. *subscapularis*) proximally to *M*. *serratus superficialis*, leaving no osteological correlates, and inserts on the medial tuberosity of the humerus.

As lepidosaurs, neornithes present a muscle complex, i.e. *Mm*. *subcoracoscapulares* (*sensu* Vanden Berge and Zweers [26]), divided into *Mm*. *subscapularis* and *subcoracoideus*. The former is also divided into two portions (*caput laterale* and *mediale*), which originate ventromedially and lateroventrally on the scapular balde and insert together on the medial tuberosity of the humerus [27, 28]. *M*. *subcoracoideus* is subdivided into two portions originating fleshily from the medial surface of the coracoid, usually sharing insertion with *M*. *subscapularis* [18, 22].

**Fig 10:**
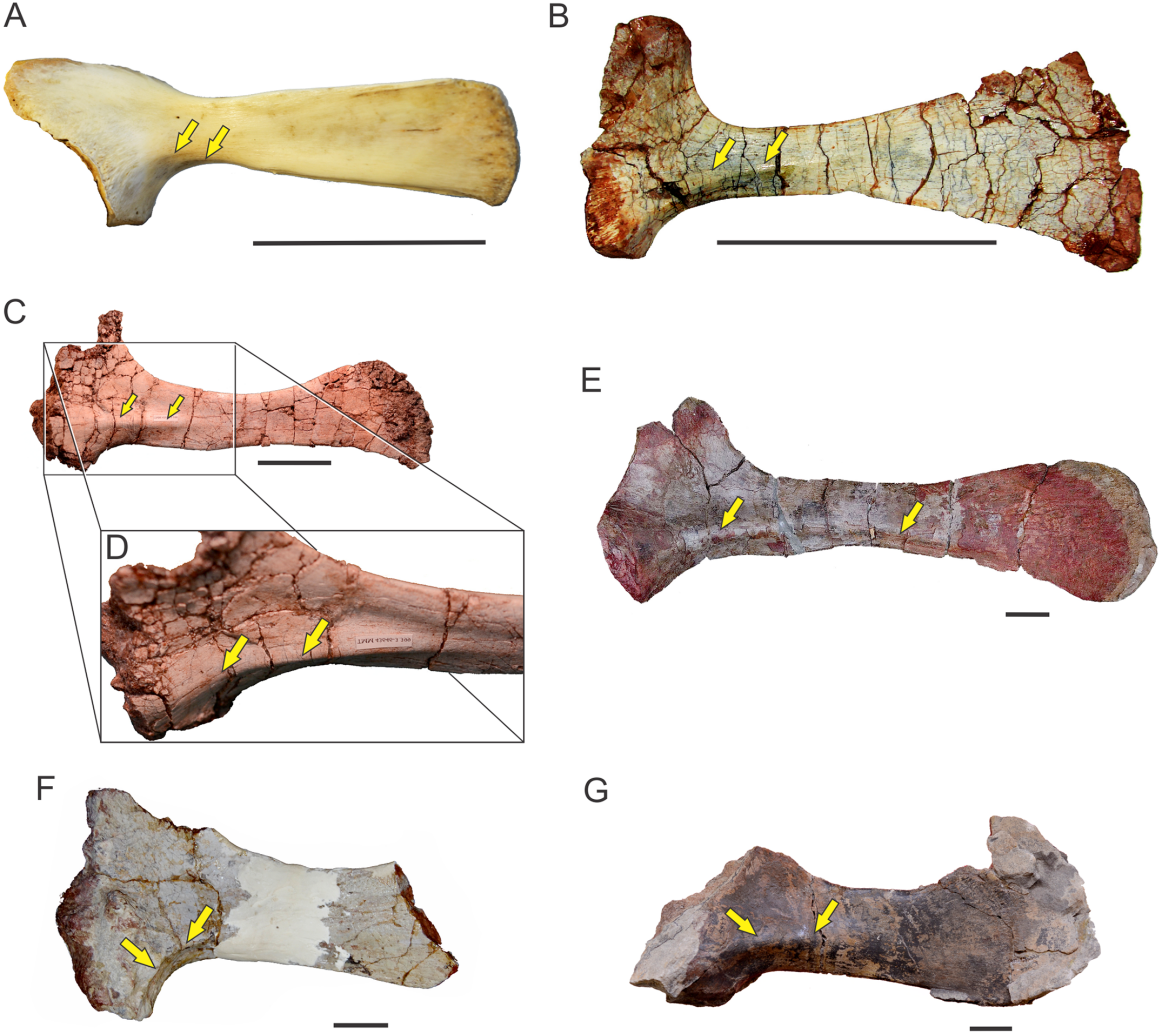
Osteological correlates on the medial side of the scapula. Ventromedial ridge (arrows) of the scapula in *Crocodylus* sp. (reversed from left) (A); *Saturnalia tupiniquim* (MPC 3844-PV) (B); S*arahsaurus aurifontanalis* (TMM 43646–2.56, reversed from right) (C), (D); *Mussaurus patagonicus* (MLP 68-II-27-1)(E); *Sefapanosaurus zastronensis* (BP/1/7433)(F); *Euskelosaurus brownii* (SAM-K386)(G). Scale bar: 5 cm.

The presence of *Mm*. *subcoracoscapulares* as a muscle complex in Sauropodomorpha is decisive positive, being the scapular head (*M*. *subscapularis*) the only present in all bracket taxa and *M*. *subcoracoideus* absent in crocodilians. Although there is not osteological correlate for the origin of this muscle (such as rugosities or striae) in basal sauropodomorphs, the medial surface of the scapular blade of several taxa carries a thick ridge running from the base and along the scapular blade, which would have served as the a structure separating *M*. *subscapularis* dorsally, and *M*. *scapulohumeralis caudalis* ventrally. This ridge (‘ventromedial ridge’, [75]) presents different degrees of development among taxa; it is present as a tenuous ridge in the analysed specimen of *Crocodylus* and well developed in various basal sauropodomorphs (e.g. *Saturnalia*, Fig 4C in [14]; *Sarahsaurus* TMM 43646–2.56; *Mussaurus* MLP 68-II-27-1; *Sefapanosaurus zastronensis* BP/1/7433; *Euskelosaurus* SAM-K386; *Leonerasaurus* MPEF-PV 1663) (Fig 10). In sauropods, however, the ventromedial ridge is not present and the medial surface of the scapular blade is rather flat. This does not necessarily mean that *M*. *subscapularis* was absent, but it was probably reduced in Sauropoda. As occurring in living archosaurs, it probably inserted on the medial tuberosity of the humerus. *M*. *subcoracoideus* would have originated from the medial coracoid leaving no osteological correlates, as in Neornithes.

This muscle possibly had more than one function in sauropodomorphs. Its medial origin on the scapula and its insertion on the proximomedial aspect of the humerus would favour adduction and some humeral pronation. As in crocodiles, it also may have aided in forelimb extension [9, 67].

### *Mm*. *scapulohumerales* (SH)

In reptiles, this muscle complex is primitively composed by two portions that cross the glenohumeral joint of the coracoid and the scapula and insert on the humerus. Lepidosaurs retain both portions. The anterior head originates from the dorsal surface of the coracoid and from the medial aspect of the scapula. The posterior portion originates along the posterior margin of the scapular blade and also partly on the suprascapula [17, 18]. Crocodilians retain the posterior portion of *Mm*. *scapulohumerales*, originating fleshily from the posterior margin of the scapular blade dorsal to the glenoid. In both lepidosaurs and crocodilians the fleshy insertion is on the proximal posterior surface of the humerus, lateral to the deltopectoral crest and dorsal to the insertion of *M*. *latissimus dorsi*.

Neornithes present both heads in this muscle. In *Gallus*, *M*. *scapulohumeralis cranialis* is much smaller than the posterior portion, the former originating fleshily from the lateral aspect of the scapular blade, dorsal to the glenoid area and inserting on the pneumatic fossa. The much larger posterior portion originates also fleshily on the lateral surface of the distal half of the scapular blade; then becomes a tendon that inserts medial to the pneumatic fossa, usually leaving no scars [22].

Phylogenetic inference allows the reconstruction of both heads of *M*. *scapulohumeralis* in Sauropodomorpha. The anterior portion would have originated on the dorsal surface of the glenoid lip of the scapula as in living lepidosaurs and birds, whereas the ventromedial ridge of the scapular blade of basal sauropodomorphs would have been the anterior boundary for the posterior head of this muscle. The ventromedial ridge presents different lengths, being long and running up to the distal third of the scapular blade in *Mussaurus* while it is shorter in other taxa (*Saturnalia*, *Plateosaurus*, *Adeopapposaurus*); it is possible that such greater development is related to the extent of the associated muscles. There are not scars for the insertion of this muscle complex but it most probably inserted on the posterior surface of the proximal humerus below the humeral head and medial to the insertion of *Mm*. *latissimus dorsi* and *levator scapulae*.

In crocodiles, *M*. *scapulohumeralis caudalis* abducts the humerus [9, 19, 57]. A similar action was probably effected in sauropodomorphs. Some extension is also expectable as the origin of this complex is located on the posterior margin of the scapular blade, thus pulling the humerus posteriorly (see also Otero *et al*. [9], Jasinoski *et al*. [22]; Burch [23]). Regarding long axis rotation, Otero *et al*. [9] identified *M*. *scapulohumeralis caudalis* as a humeral supinator in *Crocodylus* but a pronator in *Mussaurus*. The authors attributed this difference to the morphological disparity of the humerus between both taxa.

### *Mm*. *supracoracoideus* (SUC)

*M*. *supracoracoideus* is consistently present among tetrapods, increasing its complexity in the bird line. Lepidosaurs commonly present a single head, whereas crocodiles have two or three portions, and it shows a great development in birds as this muscle is deeply involved in flight [18].

In lepidosaurs (e. g., *Sphenodon*, *Gekko*) it originates along the anterior margin of the coracoid and inserts on the lateral tuberosity of the humerus proximal to the deltopectoral crest [17, 18]. *Mm*. *supracoracoideus* is composed by two [20, 21] or three [19] heads in *Alligator mississippiensis* (*pars longus*, *intermedius* and *brevis*, [19]). In *Caiman* it was possible to isolate the larger portion of *Mm*. *supracoracoideus*, although other heads could not be recognized (see also Jasinoski *et al*. [22]; Nicholls and Russell [30]). This portion originates at the anterior and medial boundary between scapula and coracoid whereas *M*. *supracoracoideus intermedius* and *brevis* originate in the same area as *longus* but on the lateral side of the scapulocoracoid [19]. Skeletonized specimens of *Caiman* and *Crocodylus* show no scars for the origin of this muscle. This complex inserts on the lateralmost margin of the deltopectoral crest of the humerus.

In Neornithes, *Mm*. *supracoracoideus* originates fleshily on several structures; in *Gallus* these include the keel, mesosternum and manubrium and also in proximal portions of clavicles and coracoid, all of them visible after removing *M*. *pectoralis* (see also Jasinoski *et al*. [22]; Vanden Berge and Zweers [26]. All portions converge to pass through the triosseous canal and insert tendinously on the lateral tuberosity of the humerus between the humeral head and the deltopectoral crest.

Phylogenetic inference allows reconstruction of a scapular and a coracoideal head for sauropodomorphs although not of additional portions. Moreover, the origin on the medial side of the coracoid or scapula seems unlikely because of the reorientation of the scapulocoracoid to a posterodorsal position in sauropodomorphs (in contrast to the vertical position in crocodiles) would have generated an unusual muscle path. The most parsimonious origin in sauropodomorphs is on the lateral surface of the coracoid and the lateral surface of proximal scapula. Although no osteological correlates exist for this muscle in living archosaurs, both the lateral coracoid and the lateral surface of the proximal scapular portion in sauropodomorphs (just posteroventrally to the acromion process) carry a depression variably developed according to the group involved. In several basal sauropodomorphs this proximal scapular depression is rather shallow, as in *Efraasia* (SMNS 12684), *Adeopapposaurus* (PVSJ 610) and *Massospondylus* (SAM-K5135), whereas in other it is more notorious (e.g. *Lufengosaurus* IVPP-V15, *Mussaurus* MLP 68-II-27-1, *Antetonitrus* BP/1/4952). In sauropods, however, the proximal scapular depression reaches its highest degree of development and it is also dorsally framed by the acromial ridge rising from the acromion process and posteriorly directed, as observed in *Camarasaurus* sp. (FMNH 25122), *Dicraeosaurus hansemanni* MB.R. mounted skeleton, *Rapetosaurus* (FMNH-PR 2209), *Giraffatitan* (HMN SII) and *Neuquensaurus australis* (MLP-CS 1096), among others. This ridge would have served as a dorsal boundary for *M*. *supracoracoideus* or either as an extra anchorage point too (Fig 9). The insertion is placed along the external margin of the deltopectoral crest as in living archosaurs, which present a rugose area for the attachment of the tendon. However, in some sauropodomorphs the margin of the crest is more thickened and this may be correlated to a stouter tendon of *M*. *supracoracoideus* (e.g. *Yunnanosaurus huangi* NGMJ 004546; *Opisthocoelicaudia skarzynski*, [15] Pl. 9-3B; *Neuquensaurus australis* MLP CS-1050).

Although this muscle has been regarded as an abductor [19, 57], a recent computational 3D moment arm analysis revealed that this complex actually has a main adductor component in both crocodiles and basal sauropodomorphs [9]. The orientation of the scapulocoracoid in crocodiles and sauropodomorphs also allows flexion and supination of the humerus [9].

### *M*. *coracobrachialis* (CB)

Plesiomorphically, *Mm*. *coracobrachiales* presents two portions. In lepidosaurs, the smaller portion (*M*. *coracobrachialis brevis*) originates on the ventrolateral surface of the coracoid and inserts between the deltopectoral crest and the medial tuberosity. The larger portion (*M*. *coracobrachialis longus*) originates on the posterolateral surface of the coracoid and then runs down to the proximal aspect of the entepicondyle [17, 18].

Crocodiles preserve the smallest portion (*M*. *coracobrachialis brevis*), which originates fleshily from most of the lateral side of the coracoid and the lateral proximal portion of the scapula below the acromion process and *Mm*. *supracoracoideus*. In one of the skeletonized specimens of *Caiman*, however, it was possible to differentiate shallow striae between the scar of *M*. *biceps* and the coracoid foramen which, by position, would correspond to the site of origin of *M*. *coracobrachialis brevis ventralis*. Both portions are regarded respectively as *M*. *coracobrachialis brevis ventralis* and *dorsalis* by Meers [19]. They insert independently on the anterior surface of the proximal humerus, medial and dorsal to the deltopectoral crest, leaving no scars.

Neornithes present two heads, *M*. *coracobrachialis cranialis* and *M*. *coracobrachialis caudalis*, both originating on the lateral aspect of the coracoid, close to the anterior end (acrocoracoid process, [26]), and on the main body of that bone, respectively [26–28]. By position, *M*. *coracobrachialis cranialis* of birds should be equivalent to *M*. *coracobrachialis brevis ventralis* of crocodiles, inserting at the base of the deltopectoral crest of the humerus [26]. In *Gallus*, *M*. *coracobrachialis caudalis* inserts tendinously on the medial side of the humeral head dorsal to the pneumatic fossa.

**Fig 11:**
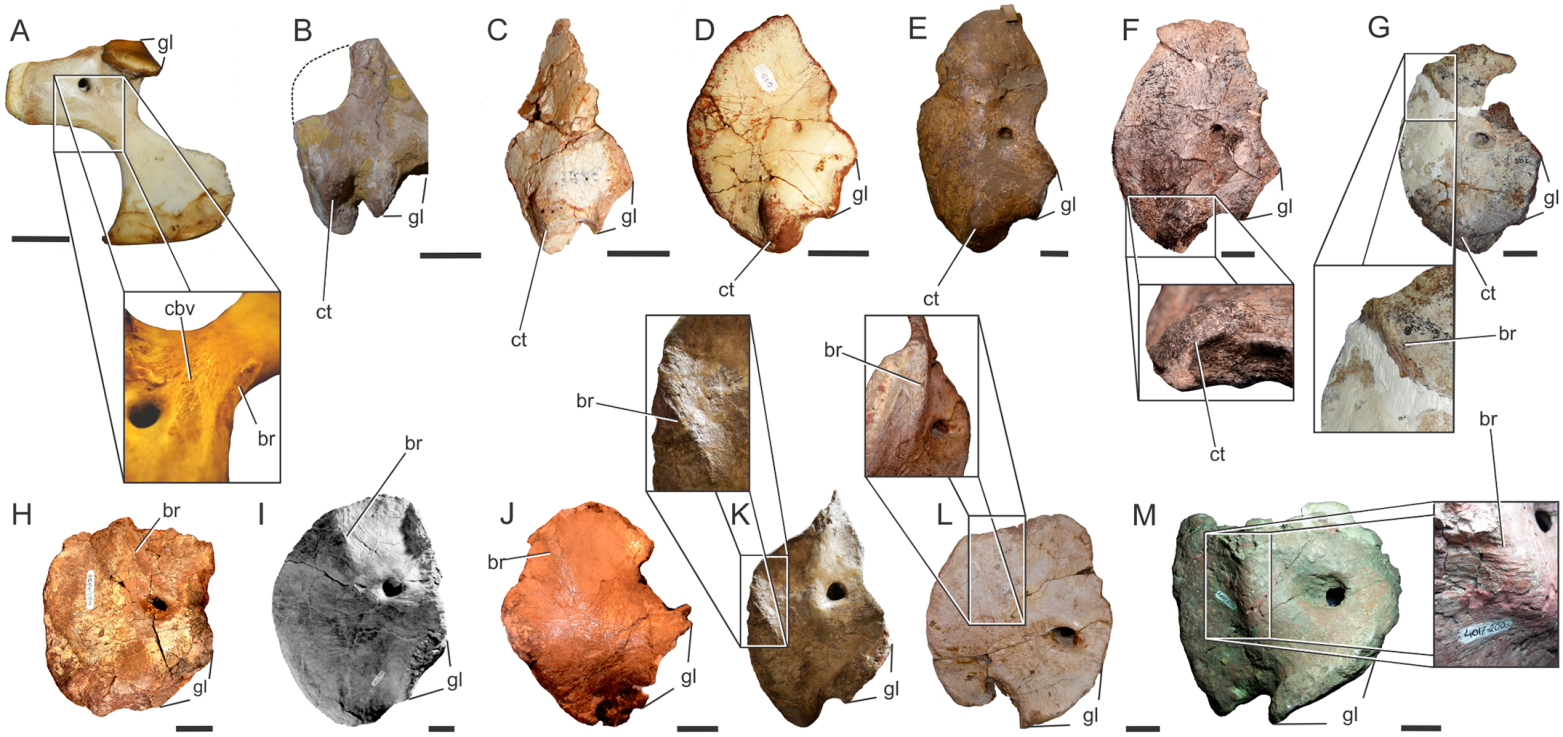
Osteological correlates on the lateral side of the coracoid among some archosaurs. *Caiman yacare* (A); *Silesaurus opolensis* (ZPAL abIII/363)(B); *Saturnalia tupiniquim* (MPC 3844-PV)(C); *Adeopapposaurus mognai* (PVSJ 610)(D); *Lufengosaurus huenei* (IVPP-V15)(E); *Sarahsaurus aurifontanalis* (TMM 43646–2.56)(F); *Sefapanosaurus zastronensis* (BP/1/7432)(G); *Antetonitrus ingenipes* (BP/1/4956)(H); *Suuwassea emiliae* (taken from Harris, Fig 1.2 in [79])(I); *Rapetosaurus krausei* (FMNH-PR 2209)(J); *Giraffatitan brancai* (HMN SII mounted skeleton)(K); *Opisthocoelicaudia skarzynskii* (ZPAL MgD-I/25c)(L); *Saltasaurus loricatus* (PVL 4017–101)(M). Abbreviations: br, *M*. *biceps brachii* ridge/scar; cbv, *M*. *coracobrachialis brevis ventralis* scar; ct, coracoid tubercle; gl, glenoid. (B), (G), (H), (I) and (K) reversed from right. Scale bars: 2 cm (A)-(G); 5 cm (H)-(M), except (K), which is not to scale.

**Fig 12:**
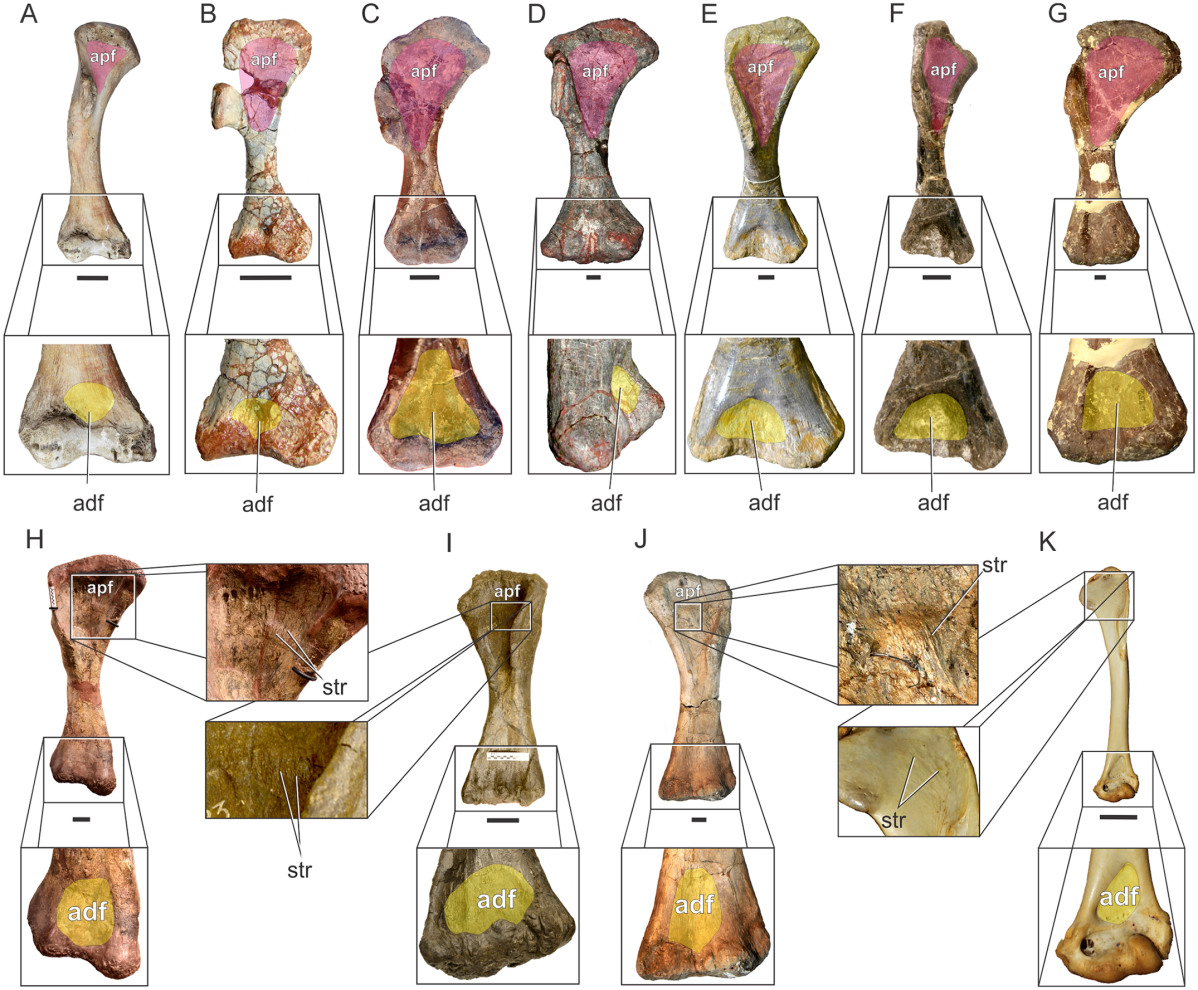
Osteological correlates on the anterior surface of the humerus in living archosaurs and Sauropodomorpha. Insertion site of *M*. *coracobrachialis brevis* on the anterior proximal fossa (apf) and origin site of *M*. *brachialis* on the anterior distal fossa (= cuboid fossa) in *Crocodylus niloticus* (A); *Saturnalia tupiniquim* (MPC 3845-PV)(B); *Massospondylus carinatus* (SAM-K5135)(C); *Coloradisaurus brevis* (PVL 5904)(D); *Plateosaurus engelhardti* (MB.R. 4404.43)(E); *Leonerasaurus taquetrensis* (MPEF-PV 1663)(F); *Antetonitrus ingenipes* (BP/1/4952)(G); *Alamosaurus sanjuanensis* (TMM 415411)(H); *Camarasaurus* sp. (AMNH 823)(I); *Elaltitan lilloi* (PVL 4628)(J); *Ciconia maguari* (MLP-O 14352)(K). Abbreviations: adf, anterior distal fossa; str, striae. Scale bar: 3 cm (A)-(G), (I); 10 cm (H)-(J).

The presence of *M*. *coracobrachialis* in Sauropodomorpha is unequivocal. However, an independent origin on the scapula brings about some speculation since this structure is not involved in the avian *M*. *coracobrachialis*. Hence, inference of the coracoid portion as a single head seems to be the least speculative in sauropodomorphs; it corresponds to the crocodilian *pars* ventralis and the avian *pars cranialis* (Level I’). Phylogenetic inference suggests an origin on the lateral surface of the sauropodomorph coracoid (and in dinosaurs in general) in which there is a fossa probably hosting the origin of *M*. *coracobrachialis*. In addition, the only putative osteological correlate associated to the origin of this muscle in living archosaurs is the acrocoracoid process of birds which, by position, corresponds to the coracoid tubercle of basal sauropodomorphs, a bump on the posterolateral surface of that bone, just below the glenoid. The coracoid tubercle is primitively present in dinosauriforms (e.g., *Silesaurus*, Fig 11B) and widely distributed among basal sauropodomorphs, such as *Eoraptor* (PVSJ 512), *Saturnalia* (Fig 4A in [14], ‘acrocoracoid tubercle’), *Adeopapposaurus* (PVSJ 610), *Lufengosaurus* (IVPP-V15), *Sarahsaurus* (TMM 43646–2.56), *Sefapanosaurus* (BP/1/7432), among others. In sauropods, conversely, the coracoid tubercle is poorly developed or non-existent at all (*contra* Otero *et al*. [48]), hence, it is possible that *M*. *coracobrachialis* originated on the deep fossa present on the proximal portion of the scapula, just posterior to the origin of *M*. *supracoracoideus* (Fig 11). The medial area on the anterior surface next to the deltopectoral crest would have been the insertion site for this muscle, although with no osteological correlate in basal sauropodomorphs. In sauropods, such surface actually becomes a deep fossa which is accentuated by the medial deflection of the deltopectoral crest in this group (see Borsuk-Bialynicka, Fig 7B in [15]; Carballido *et al*., Fig 10A in [49]; Otero, Fig 3A in [67]; Mannion and Otero, Fig 6C in [74]; Taylor, Fig 4H in [76]; Poropat *et al*., Fig 10A in[77]). Additionally, a rugose bump is present within that fossa in *Elaltitan* (PVL 4628) and *Diamantinisaurus matildae* (Fig 10A in [77]) (Fig 12).

Morphology and orientation of the coracoid and humerus produce different actions in both crocodiles and basal sauropodomorphs. In this sense, CBV acts as an extensor in the former but flexor in the later [9]. The lateral insertion on the humerus produces a pronator action in both groups also including adduction [9, 57].

### *Mm*. *triceps brachii* (TB)

The complex *Mm*. *triceps brachii* is an important muscle mass which plesiomorphically arises from the scapula, coracoid and humeral shaft although the number of heads varies among reptiles. In lepidosaurs, this muscle consists of four heads, originating from the posterior margin of the scapula, close to the glenoid area (*M*. *triceps brachii caput scapulare*), the posteromedial margin of the coracoid (*M*. *triceps brachii caput coracoideum*), and the dorsal and ventral surfaces of the humeral shaft (*M*. *triceps brachii capiti humerales*) [17, 18]. Crocodiles present five separate heads: a fully scapular, a scapulocoracoid, and three humeral, although not all of them are necessarily homologous to those found in lepidosaurs [18, 19]; hence names used herein are given only following position. *M*. *triceps brachii caput scapulare* originates via tendon from a rugose scar placed on the dorsal surface of the glenoid, visible in *Caiman and Crocodylus* but also reported in other taxa [18, 19]. *M*. *triceps brachii caput scapulocoracoideum* is composed by two portions arising from the posterior edge of the scapular blade and from the posteromedial margin of the coracoid, close to the glenoid lip. In one of the skeletonized specimens of *Caiman* and *Crocodylus* studied it was possible to recognize a scar for the origin of this portion placed on the posterior edge of the scapular blade, on the proximal third. *M*. *triceps brachii capiti humerales* cover most of the humeral shaft through a fleshy attachment and their boundaries are not recognizable.

In Neornithes, *Mm*. *triceps brachii* is reduced to three heads. The scapular head (*M*. *scapulotriceps*) originated from a similar topological surface as in crocodiles, on the dorsal surface of the glenoid area, on the lateral aspect, usually leaving a scar [18, 22] not recognizable in specimens studied. An additional origin from the humeral shaft is also reported [26, 27]. A coracoid head (*M*. *coracotriceps*), mostly tendinous, can also be found in non-ratites, associated tendinously to the scapulocoracoid contact and the sternum [18, 26]. Finally, the humeral head (*M*. *humerotriceps*) covers fleshily the posterior surface of the humeral shaft from the level of the pneumatic fossa to the distal condyles. In all cases *Mm*. *triceps brachii* insert via tendon on the olecranon process of the ulna.

**Fig 13:**
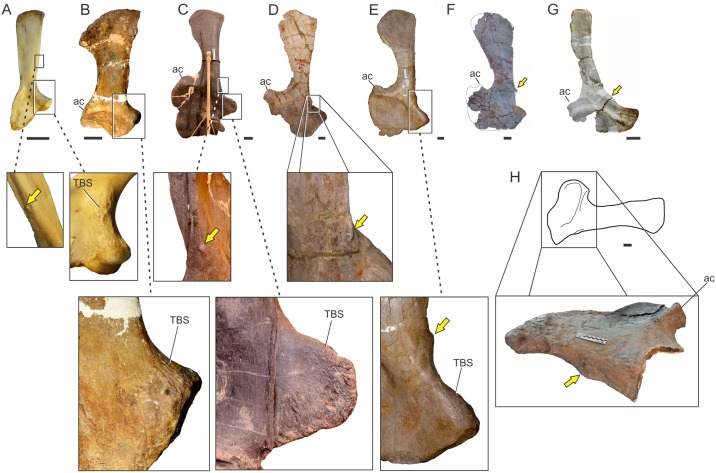
Osteological correlates on the scapula for *M*. *triceps brachii*. *Crocodylus sp*. (A); *Antetonitrus ingenipes* (BP/1/4952)(B); *Camarasaurus* sp. (FMNH 25122)(C); *Giraffatitan brancai* (MB.R. 2728)(D); *G*. *brancai* (HMN SII mounted skeleton)(E); *Europatitan eastwoodi* (taken from Torcida Fernández-Baldor *et al*., Fig 13A in [132])(F); *Vouivria dampariensis* (taken from Mannion *et al*., Fig 16A in [133])(G); *Elaltitan lilloi* (modified from Mannion and Otero, Fig 6A in [74])(H). Arrow depicts the scapular posterior tubercle. Abbreviations: ac, acromion process; TBS, *M*. *triceps brachii caput scapulare*. (F) and (H) reversed from right. Scale bars: 10 cm, except (A) which is 3 cm.

Although the presence of *Mm*. *triceps brachii* in Sauropodomorpha is unequivocal, the inference of more than two heads (apart from a scapular and a humeral one) remains controversial. A scapular head was probably present, as living reptiles retain that. In this regard, most basal sauropodomorphs show no scars or rugose surface on the dorsal side of the glenoid lip, assuming a fleshy origin for *M*. *triceps brachii caput scapulare*. Sauropods, conversely, usually present a well-developed glenoid lip of the scapula bearing a rugose dorsal surface, most probably for a tendinous origin of that muscle (e.g. *Camarasaurus* sp. FMNH 25122; *Giraffatitan* HMN-SII), such as observed in living archosaurs (Fig 13). The inference of a coracoid head is more speculative, since the homology of the avian *M*. *coracotriceps* with that of crocodilian *M*. *triceps brachii caput coracoideum* remains controversial (but see Jasinoski *et al*. [22]). The double (scapulocoracoid) origin for this head is also reconstructed here, as it is reported in *M*. *triceps brachii caput scapulocoracoideum* of crocodiles and the *M*. *coracotriceps* of some birds. In this regard, some sauropods present a rugose tubercle on the posterior margin of the scapular blade, topological equivalent to that one present in crocodiles and clearly visible in *Camarasaurus* sp. (FMNH 25122), *Angolatitan adamastor* (‘posteroventral eminence’, Fig 3A in [78]), *Daxiatitan blinglingi*, Fig 2A in [79]), *Giraffatitan* (MB.R. 2728 and HMN SII mounted skeleton), *Chubutisaurus insignis* (‘ventro medial process’, Fig 9A in [49]), *Ligabuesaurus leanzai*, Fig. 6B in [78]), *Elaltitan* (PVL 4628, ‘posteroventral process’, Fig 6A, B in [74]) and probably *Phuwiangosaurus sirindhornae*, Fig 10 in [81]). Such tubercle is placed in a similar topological position as that of *M*. *triceps brachii caput scapulocoracoideum* in crocodiles, thus probably corresponding to that portion (Fig 13) despite the fact that no osteological correlate exists for the latter group. Regarding the humeral head, phylogenetic inference allow the reconstruction of two portions in sauropodomorphs (one lateral and one medial) as in living archosaurs. In this regard, the humeral shaft of *Plateosaurus engelhardti* (MB.R. 4430.163) presents a conspicuous ridge running from the lateral side of the entepicondyle, extending proximally along the shaft which probably served as a divide for the origin site of the humeral heads. In the same way, Cooper [29] reports a similar ridge proximodistally oriented on the lateral side of the humeral shaft of *Massospondylus* which also may correspond to the *M*. *triceps caput humeralis* division, although those were not figured by that author. As in all living reptiles, the insertion of *Mm*. *triceps* in sauropodomorphs would have been via tendon onto the prominent olecranon process of the ulna in basal forms and on the same topological area in sauropods.

The posterior position of the line of action of *Mm*. *triceps brachii* relative to the elbow joint makes it the main elbow extensor, as in living reptiles. Additionally, the scapular head also assists in forelimb extension.

### *M*. *biceps brachii* (BB)

*M*. *biceps brachii* is present in all amniotes with a relatively conservative path, running on the anterior side of the forelimb, from the coracoid to the proximal antebrachium. In lepidosaurs it originates fleshily on the anterior or ventral surface of the coracoid—depending on the group—and inserts on the anteroproximal surface of the ulna and radius [17, 18]. In crocodiles, it originates via tendinous attachment on the anterior and lateral surface of the coracoid close to the scapulocoracoid contact; it usually leaves a visible longitudinal scar (Fig 11) [18, 19, 22]. In dissected specimens of *Caiman* such scar was not recognizable, most probably because specimens were non-adults; yet, it is clearly visible in skeletonized adult specimens. It inserts on the proximomedial surface of the radius, down to the insertion of *M*. *humeroradialis*, although an insertion on the proximal ulna is also reported [18].

In Neornithes, *M*. *biceps brachii* is a much more massive muscle than in lepidosaurs and crocodiles. Despite the fact that Vanden Berge and Zweers [26] reported no origin from the coracoid, this area is cited as an attachment site, more specifically as a tendinous origin of the acrocoracoid process of the coracoid [18, 27, 28]. The second origin site corresponds to the bicipital crest of the humerus, via aponeurosis clearly visible in dissected specimens of *Gallus*. Close to the elbow joint the tendon bifurcates, inserting on the proximal aspect of both radius and ulna.

**Fig 14:**
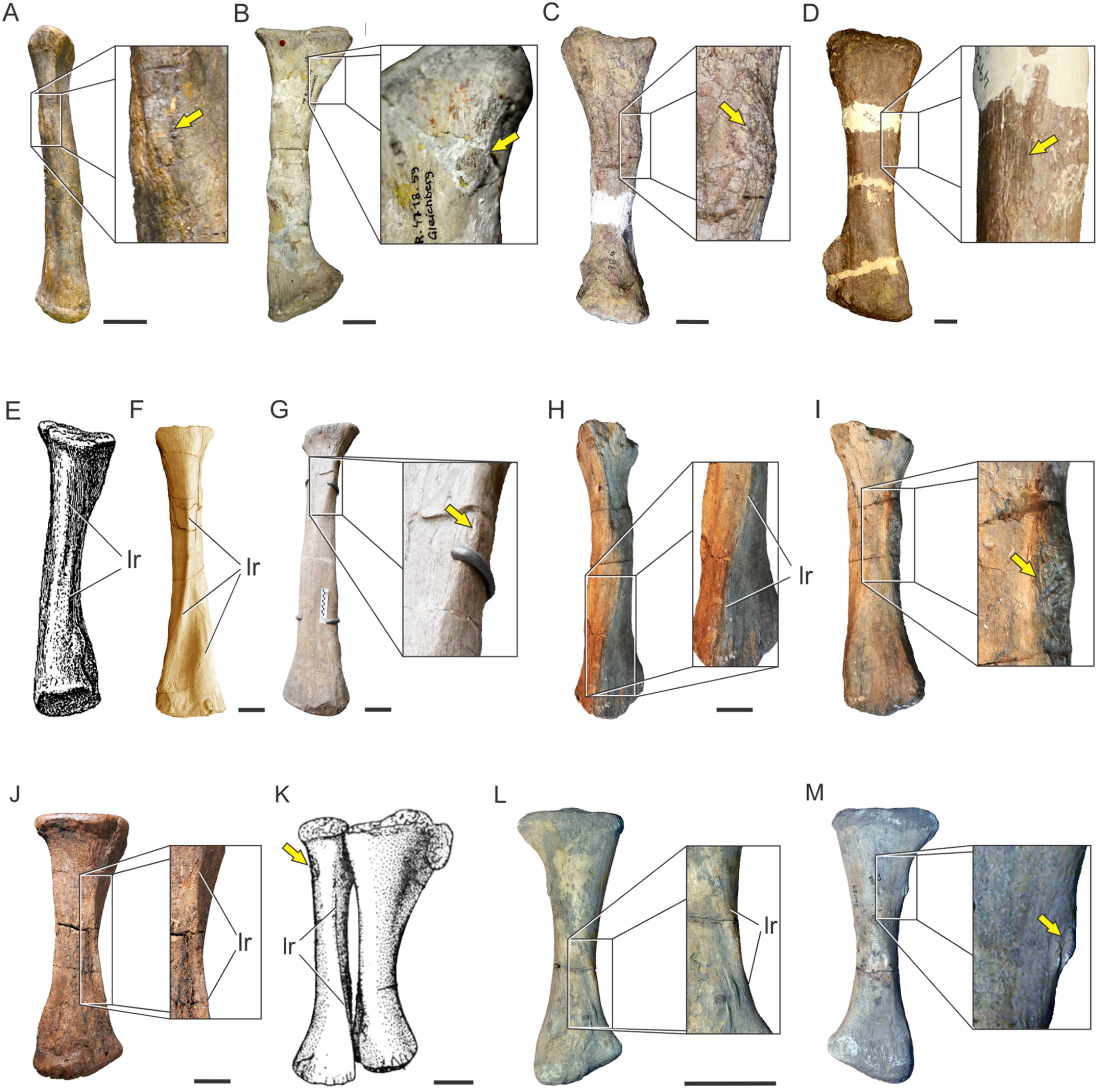
Osteological correlates on the radius in Sauropodomorpha. Sauropodomorph radii depicting the *M*. *biceps brachii* scar (arrow) and the longitudinal ridge (lr) in *Plateosaurus engelhardti* (MB.R. 4404.46 skelett 25) in medial view (A); *Ruehleia bedheimensis* (MB.R. 4718.59) in anterior view (B); *Sefapanosaurus zastronensis* (BP/1/7435) in anterior view (C); *Antetonitrus ingenipes* (BP/1/4952) in anterior view (D); *Apatosaurus louisae* (Reprinted from Gilmore, Fig 12A in [86] under a CC BY license, with permission from Carnegie Museum of Natural History, original copyright 1936) in posterior view (E); *Rapetosaurus krausei* (FMNH-PR 2209) in posterior view (F); *Giraffatitan brancai* (HMN SII mounted skeleton) in anteromedial view (G); *Elaltitan lilloi* (PVL 4628) in lateral (H) and anterior (I) views; *Diamantinisaurus matildae* (AODF 603) in posterior view (J); *Opisthocoelicaudia skarzynskii* (Reprinted from Borsuk-Bialynicka, Fig 8C in [15] under a CC BY license, with permission from Instytut Paleobiologii PAN, original copyright 1977) in medial view (K); *Neuquensaurus australis* (MLP-CS 1169) in anterior (L) and posterior (M) views. Scale bar: 3 cm (A)-(D), (F); 10 cm (G)-(M); not to scale (E).

Phylogenetic inference in sauropodomorphs allows an unambiguous reconstruction of the origin site on the anterior and lateral surfaces of the coracoid. Meers [19] reported a prominent longitudinal scar running parallel to the shaft of the coracoid in crocodiles as the osteological correlate for this muscle and present in adult *Caiman* specimens analysed. A similar scar is observed on the anterior side of the coracoid of some sauropodomorphs (e.g. *Sefapanosaurus* BP/1/7424, *Antetonitrus* BP/1/4956, *Suuwassea emiliae*, Fig 1.2 in [82]; *Giraffatitan* HMN SII mounted skeleton; *Rapetosaurus* FMNH-PR 2209 mounted skeleton, *Opisthocoelicaudia* ZPAL MgD-I/25c; *Saltasaurus loricatus* PVL 4017–101), suggesting a more anterior origin of *M*. *biceps brachii* than previously thought (Fig 11). The additional origin in the humerus requires more speculation (Level II’). The anteromedial side of the proximal radius of several sauropodomorphs presents a conspicuous scar that probably hosted both the insertions of *M*. *humeroradialis* and *M*. *biceps brachii*, allowing an unambiguous reconstruction of those muscles; the insertion on the proximal ulna remains equivocal. This scar is evident in *Plateosaurus engelhardti* (MB.R. 4404 skelett 25), *Sefapanosaurus* (BP/1/7435), *Antetonitrus* (BP/1/4952), *Giraffatitan* (HMN SII mounted skeleton), *Elaltitan* (PVL 4628), *Neuquensaurus* (MLP-CS 1169) and *Opisthocoelicaudia* (Fig 8B in [15]) (Fig 14). *Rueheleia* (MB.R. 4718) also shows a scar, albeit more posteriorly placed, that also could correspond to the insertion site of *M*. *biceps brachii*.

As in living archosaurs, the main action of *M*. *biceps brachii* in sauropodomorphs would have been flexion the antebrachium. Subordinate actions for this muscle include adduction, supination and even mixed roles [9].

### *M*. *humeroradialis* (H)

This muscle is scantily present in reptiles, only reported in *Sphenodon* and crocodilians. In *Sphenodon*, *M*. *humeroradialis* shows no contact with osseous structures [18]. In crocodiles it originates fleshily from the anterolateral surface of the humeral shaft, distal to the insertion site of *M*. *deltoideus clavicularis* and the deltopectoral crest, usually leaving a scar [18, 19] that is very tenuous or nonexistent in skeletonized specimens analysed. It inserts on the proximomedial surface of the radius, on a conspicuous scar widely present among crocodilian species [19].

In Neornithes, some authors suggest that *M*. *humeroradialis* is a derivative of the complex *Mm*. *deltoideus* [48], being *pars propatagialis* its avian homologue [18]. However, this interpretation is not well-supported.

Considering that among living archosaurs *M*. *humeroradialis* is only conclusively present in crocodilians, its inference in sauropodomorphs is equivocal. The scar pattern reported for crocodilians on the humeral shaft below the deltopectoral crest is not present in sauropodomorpha, although for the insertion there is a rugose area on the proximomedial surface of the radius; this area would have hosted the tendon of *Mm*. *humeroradialis* and *biceps* (see above).

Like *M*. *biceps brachii*, *M*. *humeroradialis* would have flexed the antebrachium.

### *M*. *brachialis* (B)

Together with *M*. *biceps brachii* and *M*. *humeroradialis*, *M*. *brachialis* is the third muscle running along the anterior surface of the forelimb. In lepidosaurs it originates fleshily on the humeral shaft distal to the deltopectoral crest, with variable origin sites depending on the group, and inserts via tendon on the proximal surface of ulna and radius [17, 18]. In crocodilians, the origin of *M*. *brachialis* is fleshy, running alongside *M*. *humeroradialis* along the ventral surface of the humeral shaft distal to the deltopectoral crest. It inserts on the proximal surface of the radius [19], although a shared tendon with *M*. *biceps brachii* is also reported inserting also on the proximal ulna [18].

In Neornithes *M*. *brachialis* is reduced and confined to the elbow joint area. It originates fleshily from the distal end of the humerus proximal to the entepicondyle, on the *fossa brachialis*; it inserts on the *depressio brachialis* proximal to the ventral aspect of the proximal ulna [27, 28], although an additional radial insertion is also reported [18].

The presence of *M*. *brachialis* in sauropodomorphs is unequivocal, but its origin and insertion is somewhat speculative. The origin of this muscle in crocodilians and birds is placed in different positions along the humeral shaft. This, together with the fact that no scars are present in the humerus of sauropodomorphs, suggests that the origin of *M*. *brachialis* in this group should have been on the anterior surface of the shaft, somewhere between the deltopectoral crest and the distal condyles. One possible site of origin of *M*. *brachialis* in sauropodomorph dinosaurs is the fossa placed between distal condyles (e.g. ‘fossa *M*. *brachialis*’, [14]; ‘cuboid fossa’, [75]). This fossa presents different degrees of development, being rather shallow in basal sauropodomorphs (e.g. *Saturnalia*, Fig 6C in [14],; *Plateosaurus engelhardti* MB.R. 4404.44; *Adeopapposaurus* PVSJ 610; *Coloradisaurus*, Fig 7A in [83]; *Leonerasaurus* MPEF-PV 1663; *Antetonitrus* BP/1/4952)—although in *Ruehleia* (MB.R. 4718.39) the fossa is well-developed—and almost inexistent and scarcely present in sauropods (e.g. *Elaltitian* PVL 4628; *Bonitasaura*, Fig 11B in [60]) (Fig 12). The radius and the ulna of sauropodomorphs carry no additional scars for the attachment of *M*. *brachialis*. Thus, the presence of this muscle with fleshy attachment on both antebrachial bones is not ruled out.

### *M*. *supinator* (S)

*M*. *supinator* is one of the various muscles originating in the distal condyles of the humerus and inserts on the antebrachium, with a relatively constant path across tetrapods. In both lepidosaurs and crocodiles it originates fleshily on the lateral side of the ectepicondyle, although leaving a scarred pattern in the latter (*Crocodylus niloticus*); it inserts also directly onto the medial side of the radial shaft ([18, 19], own observations), although a subdivision of this muscle is reported in the geckoniid *Eublepharis* [17]. In Neornithine birds this muscle has a tendinous origin, showing a pitted pattern for tendon attachment ([18, 27], own observations), whereas its insertion is fleshily distal to the biceps tubercle.

**Fig 15:**
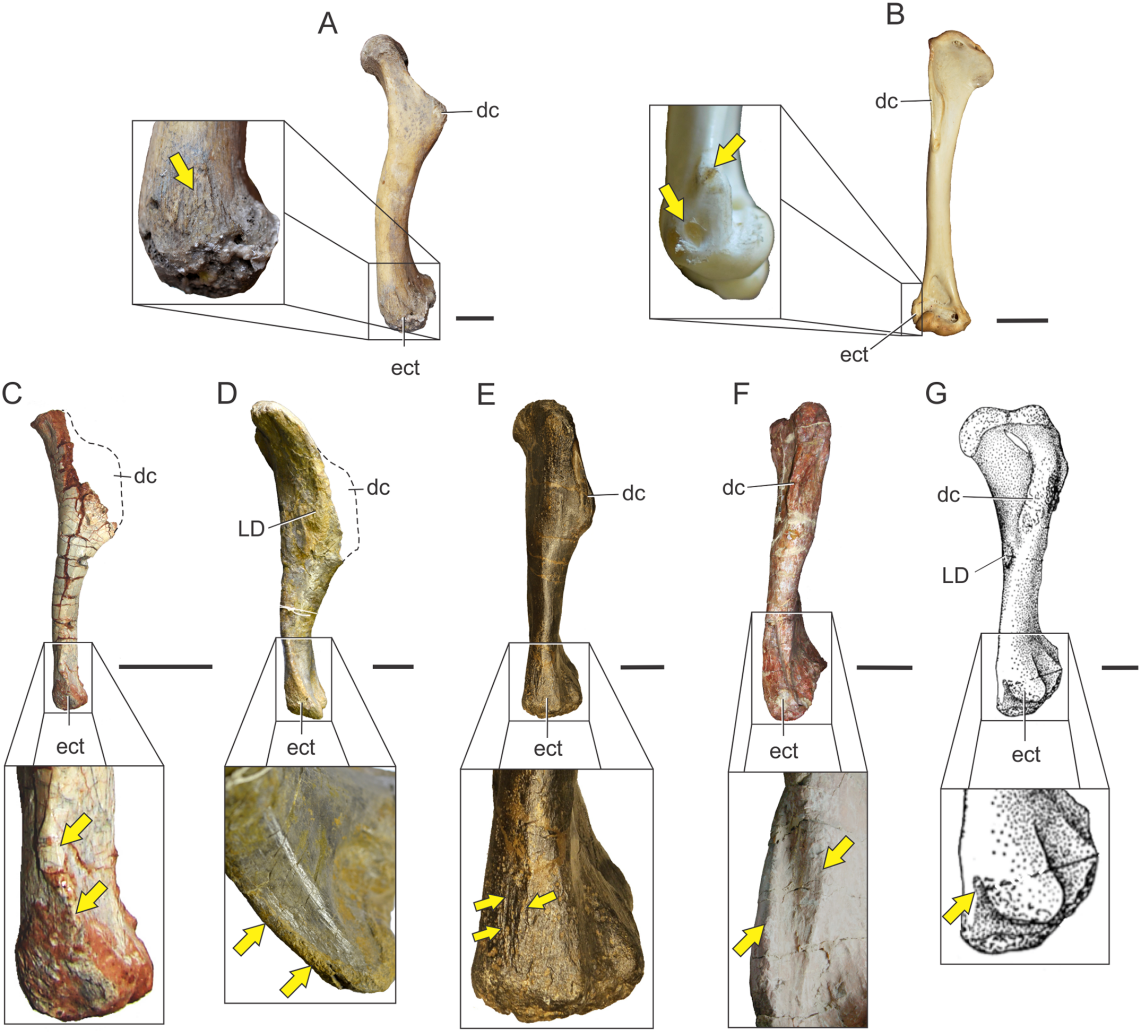
Osteological correlates on the distal humerus in living archosaurs and Sauropodomorpha. Origin site (arrow) of antebrachial muscles on the lateral surface of the ectepicondyle in *Crocodylus niloticus* (A); *Sarcoramphus papa* (MLP-O 14362) (B); *Saturnalia tupiniquim* (MPC 3844-PV)(C); *Plateosaurus engelhardti* (MB.R. 4404.43 skelett 25)(D); *Camarasaurus* sp. (AMNH 664)(E); *Saltasaurus loricatus* (PVL 4017–67)(F); *Opisthocoelicaudia skarzynskii* (Reprinted from Borsuk-Bialynicka, Fig 7C in [15] under a CC BY license, with permission from Instytut Paleobiologii PAN, original copyright)(G). Abbreviations: ect, ectepicondyle; dc, deltopectoral crest; LD, *M*. *latissimus dorsi* insertion site. Scale bar: 3 cm (A)–(D); 10 cm (E)-(G).

The presence of *M*. *supinator* in sauropodomorphs is unequivocal. It probably originated, as in living reptiles, on the lateral surface of the humeral ectepicondyle. As in the origin site of various antebrachial muscles such as *Mm*. *supinator*, *flexor ulnaris*, *abductor radialis*, *extensor carpi radialis* and *extensor digitorum longus*, it shows no scars or ridges as in most sauropodomorphs (e.g. *Adeopapposaurus* PVSJ 610, *Gyposaurus sinensis* IVPP-V26; *Lufengosaurus* IVPP-V15; *Yunnanosaurus* NGMJ 004546; *Antetonitrus* BP/1/4952). However, in other sauropodomorphs, the surface immediately above the lateral condyle carries a variety of structures that would have hosted ligaments for these muscles anchoring on the ectepicondyle area, as observed in living crocodiles (e.g. *C*. *niloticus*). For instance, *Saturnalia* carries a proximodistally elongated groove (‘ligament groove’ *sensu* Langer *et al*. [14] Fig 6C); conversely, *Plateosaurus engelhardti* (MB.R. 4404.43) shows a well-developed longitudinal ridge with a notably rugose area at its base, whereas *Ruehleia* (MB.R. 4718.104) exhibits a tenuous rugose area. Among sauropods, *Camarasaurus* sp. (AMNH 664), *Saltasaurus* (PVL 4017–67) and *Opisthocoelicaudia* (Fig 7C in [15]) also carry a pattern of crests and rugosities on the lateral condyle as the correlate of antebrachial muscles (Fig 15). The absence of scars on the anteromedial surface of the shaft of the radius suggests a direct insertion.

In crocodiles the main action of *M*. *supinator* is to supinate the antebrachium, with a secondary flexion action [19, 57]. As active pronation/supination was probably severely reduced or even absent in dinosaurs [7, 84], the sole action of this muscle may have been flexion assistance.

### *M*. *flexor ulnaris* (FU)

This muscle is primitively present in all tetrapods, but lost in squamates. As a general pattern in lepidosaurs, crocodiles and Neornithes ([18, 19], own observations), it runs tendinously from the ectepicondyle to the anterolateral ulnar shaft via a long, fleshy attachment; the same pattern was probably present in Sauropodomorpha.

### *M*. *abductor radialis* (AR)

This is another muscle originating tendinously from the ectepicondyle and inserting on the anterolateral surface of the radius in both lepidosaurs and crocodiles, but absent in birds; hence, inference in sauropodomorphs is equivocal. If present, *M*. *abductor radialis* would have the same path as in crocodiles, although the absence of osteological correlates in both living and extant reptiles precludes establishing boundaries for the attachment of this muscle. Although Meers [19] stated an abductor action for this muscle in crocodiles, recent work demonstrated an extension action for *Crocodylus johnstoni* [9] and a combined flexion/extension action in sauropodomorphs, as mediolateral movements of the antebrachium would have been severely reduced in dinosaurs.

### *M*. *extensor carpi radialis* (ECR)

This muscle, also known as *M*. *extensor carpi radialis longus* [19, 57] is plesiomorphically present in all tetrapods with a rather conservative pattern, arising from the ectepicondyle and inserting on the radiale but also on other structures surrounding the carpus. In lepidosaurs (*Sphenodon*) its origin is tendinous and is composed by a superficial portion, inserting on the dorsal surface of the radiale, and an intermedius head, inserting on the distal and anterior aspect of the radius [18]. The pattern in crocodilians and birds is similar, although both groups have lost the intermedius portion (but Allen *et al*. [57] reported two heads for this muscle). In crocodilians, the insertion is on the radiale, whereas in birds *M*. *extensor carpi radialis* inserts on the carpometacarpus on the side of metacarpal I ([18, 26], own observations).

The origin of *M*. *extensor carpi radialis* in sauropodomorphs is unequivocal. However, its insertion is ambiguous because sauropodomorphs retain only distal carpals. If this muscle was actually present in this group, its insertion probably was topologically more similar to that of birds, i.e. on or close to metacarpal I. In this sense, the most parsimonious hypothesis for the insertion of *M*. *extensor carpi radialis* in sauropodomorphs is on the distal carpal one, which is commonly the largest among sauropodomorphs and topological equivalent to the insertion in living archosaurs. The main action of this muscle would have been extension of the wrist, as in living crocodiles [19, 23], but a combined flexion/extension action for basal sauropodomorphs is also plausible [9].

### *M*. *extensor digitorum longus* (EDL)

This muscle (*M*. *extensor carpi ulnaris longus* sensu Meers [19]) is primitively present in all tetrapods, in all cases arising from the ectepicondyle and inserting via tendon—with different reductions—on the metacarpus. The primitive condition for lepidosaurs (*Sphenodon*) is an insertion on the proximolateral surface of metacarpals I to IV [17], although squamates show a reduction of this condition, avoiding the tendon of metacarpal I [17]. Crocodiles only retain insertion tendons on metacarpals II and III, whilst in birds the insertion is at the proximal end of first phalanx of digit I and II ([18, 19, 23], own observations).

*M*. *extensor digitorum longus* is unambiguously present in sauropodomorphs with an unequivocal origin on the humeral ectepicondyle. Its insertion, however, can only be confidently inferred on digit II, whereas remaining digits I and III are controversial (Level II’ inference). In this sense, the insertion on the proximodorsal surface of metacarpal II is unequivocally present in turtles, lepidosaurs, and crocodiles [17–19, 23]; it is maintained in sauropodomorphs, although with no osteological correlates. The precise insertion site, also speculative, is located in sauropodomorphs on the proximal end of second metacarpal, as in non-avian reptiles, considering that the insertion on phalanges is an avian specialization.

As *M*. *extensor digitorum longus* runs dorsally on the antebrachium and inserts also dorsally on the metacarpals crossing the wrist joint, its action would have been a wrist extensor, as in living archosaurs [19, 23], although a combined extensor/flexor action was reported for *Mussaurus* [9].

### *M*. *flexor carpi ulnaris* (FCU)

This antebrachial muscle has an origin consistently placed on the entepicondyle of the humerus among reptiles [17–19]. In crocodilians its origin is placed distal and posterior to other antebrachial muscles arising from the entepicondyle. In this group *M*. *flexor carpi ulnaris* inserts on the pisiform. In birds, its origin is also from the entepicondyle,—situated on the *processus flexorius*—and inserted on the *processus muscularis* of the ulnare [26].

The origin of *M*. *flexor carpi ulnaris* in sauropodomorphs is unequivocally placed on the entepicondyle of the humerus, although with no clear osteological correlate. Its insertion, however, requires more speculation since neither pisiform nor ulnare are reported in sauropodomorph dinosaurs. Therefore, the distal carpus should be considered as an alternative hypothesis for insertion of this muscle in Sauropodomorpha (but see Borsuk-Bialynicka [15] for an alternative insertion].

More than one action has been mentioned for this muscle [19], although flexion of the wrist was probably the most important one performed by *M*. *flexor carpi ulnaris*.

### *M*. *extensor carpi ulnaris* (ECU)

This is another muscle originating from the ectepicondyle of the humerus, inserting on the carpus area, commonly on the lateral aspect, either on pisiform, ulnare, or even on the proximal surface of metacarpal V [17, 18]. This muscle does not seem to correspond to *M*. *extensor carpi ulnaris longus* of Meers [19] and is regarded as absent in crocodilians [18, 23, 85]. In birds, the origin is the same, but inserting on the proximal surface of metacarpal II (*proc*. *intermetacarpalis sensu* Vanden Berge and Zweers [26].

*M*. *extensor carpi ulnaris* is reported in non-archosaur reptiles and birds as well; hence, the absence in living crocodilians is considered as a consequence of loss at the base of that clade. Thus, the inference of *M*. *extensor carpi ulnaris* in non-avian dinosaurs is most probably correct, optimizing at the base of Dinosauria as a decisive positive assessment. It possibly originated from the distal ectepicondyle, as in living reptiles, and inserted on the proximodorsal surface of metacarpal II, as in living birds, especially considering the absence of preserved elements of the proximal carpus and pisiforms in sauropodomorphs.

Considering the line of action running along the dorsal surface of the wrist joint and attaching dorsally on metacarpal II, *M*. *extensor carpi ulnaris* most probably acted as an extensor of the manus.

### *M*. *pronator teres* (PT)

Like the previous muscle, *M*. *pronator teres* is primitively present in all reptiles with some variations, but in this case always arising via tendon from the humeral entepicondyle and inserting directly on the radial shaft. In crocodiles, the origin of this and other muscles attaching onto the lateral surface of the entepicondyle are related to proximodistally oriented striae, although those are only present in adult specimens (own observations). The insertion on the medial radial shaft leaves no scars [18, 19].

Neornithes present a subdivision of *M*. *pronator*, namely *M*. *pronator superficialis* and *M*. *pronator profundus* [26, 27]. The former originates more proximally on the entepicondyle (i.e. *tuberculum supracondylare sensu* Vanden Berge and Zweers [26]) and covers the *profundus* division along all of its length; the latter originates from the *epicondylus ventralis* [26]. Both the *tuberculum supracondylare* and the *epicondylus ventralis* are present as rounded pits in analysed skeletonized specimens of *Ciconia maguari* and *Sarcoramphus papa*. In *Gallus*, both portions cover the anterior radial shaft until the last third of the bone, but in other forms these muscles cover only the proximal third [26]. Although the insertion of *M*. *pronator teres* is regarded as fleshily in most cases [18], McKitrick [27] reported a tendinous attachment in Gaviiformes. In this regard, the intermuscular lines present in the radius [26] could be the osteological correlate for this muscle.

*M*. *pronator teres* is unequivocally present in sauropodomorphs, although a secondary head remains equivocal. There are osteological correlates that might correspond to the origin of *M*. *pronator teres* among sauropodomorphs, such as the lateral surface of the entepicondyle of *Plateosaurus engelhardti* MB.R. 4430.163, which presents a thick ridge running from the entepicondyle to the shaft midlength. Among sauropods, *Camarasaurus* sp. AMNH 823 presents deep striation on the lateral surface of the entepicondyle, as that observed in living crocodiles, but differing from the pitted pattern observed in living birds. The insertion is probably correlates with a longitudinal ridge (or ridges) running parallel to the radial shaft, on the ulnar side, which actually would have separated the insertion site of *M*. *pronator teres* from the insertion site of *M*. *pronator quadratus* on the ulnar side of the radius. Such structures are reported in the basal sauropodomorph *Saturnalia* (Fig 9 in [14]) and a similar ridge is present in *Plateosaurus engelhardti* (MB.R. 4404.46). Among sauropods, they are present in *Apatosaurus louisae* (Fig 12B in [86]), *Rapetosaurus* (FMNH-PR 2209), *Elaltitan* (PVL 4628), *Opisthocoelicaudia* (Fig 8C in [15]), *Neuquensaurus* (MLP-CS 1169), and *Diamantinisaurus* (Fig 12E in [77]) among others. The longitudinal radial ridges are also reported in theropod dinosaurs (Fig 86E in [87]) and most probably correspond to the *linea intermuscularis* described for birds (Fig 4.13B, C in [26]; see also Langer *et al*. [14]) (Fig 14).

The pronator action reported for crocodilians [19, 57] would have been precluded and flexion seems to be preponderant [9].

### *M*. *pronator quadratus* (PQ)

It is a short but wide muscle running from the ulna to the radius (non-avian reptiles) or narrow and inserting onto the carpus in birds. Additionally, in squamates it can also have an accessory portion arising from the humeral entepicondyle [18, 23]. In crocodiles *M*. *pronator quadratus* runs from the radial side of the shaft of the ulna to the ulnar side of the shaft of the radius ([18, 19], own observations). In specimens analyzed no scars are present for this muscle. The avian *M*. *ulnometacarpalis ventralis* also originates on the ulna (distal end) but inserts tendinously at the proximal end of metacarpal I [18, 27].

**Fig 16:**
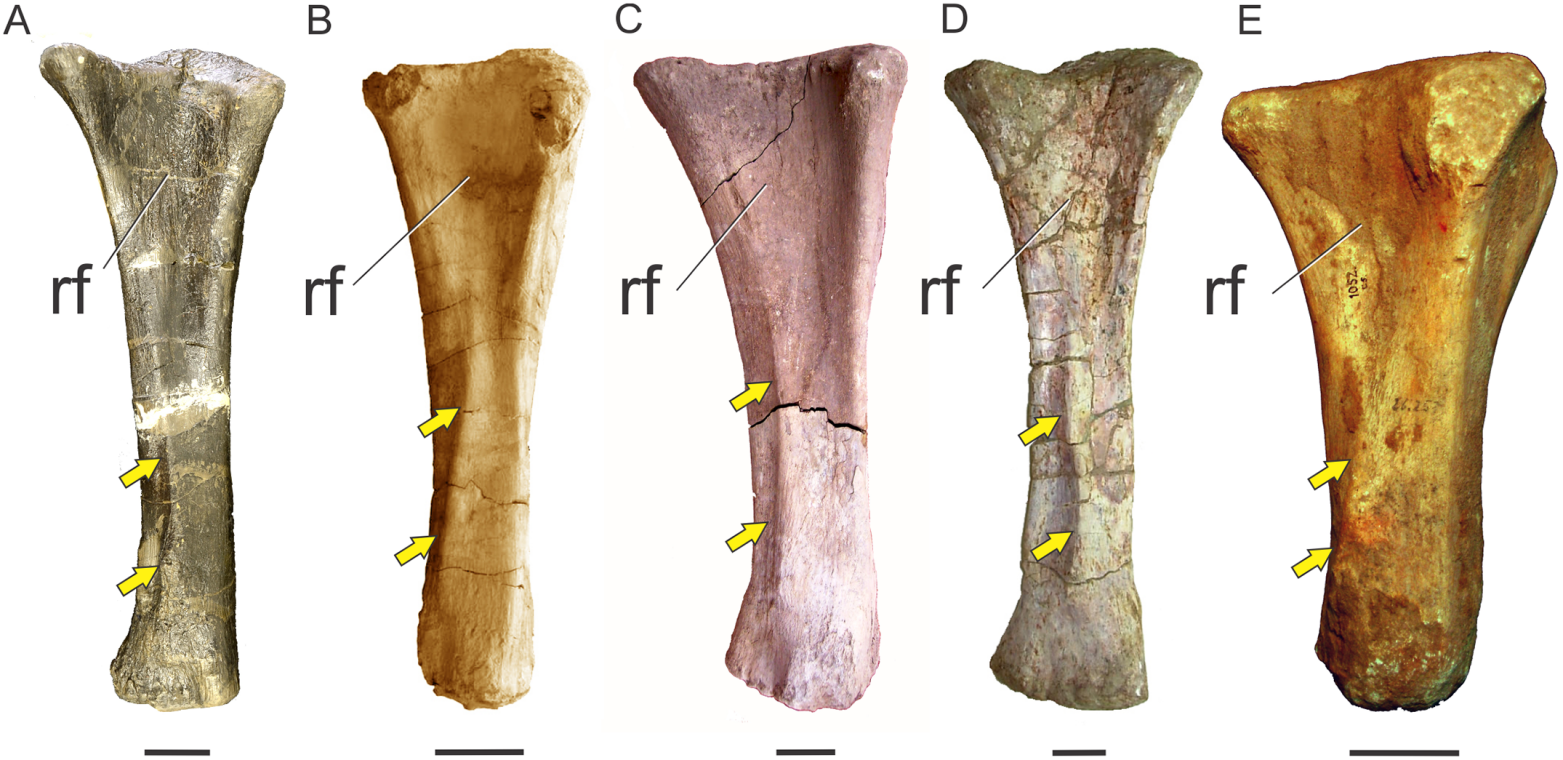
Osteological correlates of *M*. *pronator quadratus* in Sauropoda. Ulnae in radial view depicting the ulnar ridge in *Camarasaurus* sp. (AMNH 332)(A); *Rapetosaurus krausei* (FMNH-PR 2209)(B); *Bonitasaura salgadoi* (MPCA 467)(C); *Narambuenatitan palomoi* (MAU-Pv-N-425)(D); *Neuquensaurus australis* (MLP-CS 1052)(E). Abbreviation: rf, radial face. Scale bar: 5 cm.

The origin site of *M*. *pronator quadratus* is unequivocal in Sauropodomorpha, although with no scarring pattern as in living archosaurs. The insertion is equivocal due to the variation existing in living archosaurs. Considering the primitive radial insertion among non-avian reptiles, this muscle is herein reconstructed in this way for sauropodomorphs. The ulna of basal sauropodomorphs usually lacks any sign of ridges or notable muscle scars, thus precluding it as origin site of *M*. *pronator quadratus* (but see Langer *et al*., Fig 8C in [14], which reported an osteological correlate for the origin of this muscle proximally on the ulnar shaft, and a similar ridge is also reported in the basal ornithischian *Heterodontosaurus*, ‘ulnar ridge’ *sensu* Santa Luca [88]). Unlike the pattern observed in living crocodiles and birds, in several sauropods the ulna presents a longitudinal ridge on the radial side that may have been the osteological correlate of this muscle. Such a structure is usually placed in the distal half of the ulna and clearly evident in *Camarasaurus* sp. (AMNH 332), *Rapetosaurus* (FMNH-PR 2209), *Giraffatitan* (HMN SII mounted skeleton), *Bonitasaura* (Fig 12D in [59]), *Narambuenatitan palomoi* (MAU-Pv-N-425), *Opisthocoelicaudia* (Fig 8C in [15]), and *Neuquensaurus* (MLP-CS 1052). A similar ulnar ridge is also reported in the basal saurischian *Sanjuansaurus gordilloi* (‘anterior ridge’, Fig 6E in [89]) pointing towards a primitive presence within Dinosauria (Fig 16). The insertion of *M*. *pronator quadratus* is correlated to the radial longitudinal ridge or ridges mentioned in the previous muscle.

This muscle would have stabilized both bones of the antebrachium in sauropodomorphs, disregarding here the pronation action inferred for crocodiles [19, 57].

### *M*. *abductor pollicis longus* (APL)

*M*. *abductor pollicis longus* (*M*. *extensor carpi radialis brevis sensu* Meers [19]; Allen *et al*. [57]) is present in all reptiles. In lepidosaurs it originates from the distal end of the ulna and attaches to the proximomedial side of metacarpal I [18], whereas in crocodiles and birds, a radial head is added [18, 19, 23]. In crocodiles, the origin of *M*. *abductor pollicis longus* extends fleshily along the lateral shaft of radius and ulna, and both heads insert together via tendon on the dorsal surface of the radiale. In Neornithes, its homologue, *M*. *extensor longus alulae* display a similar pattern to that in crocodiles, although its insertion is on the proximal end of the extensor process of the carpometacarpus, [18, 26, 27].

This muscle is unequivocally present in sauropodomorphs, with two origin sites and leaving no osteological correlates, as in living archosaurs. The speculation arises when considering its insertion, since the lack of radiale in Sauropodomorpha hampers recognition of a precise attachment. In this way, *M*. *abductor pollicis longus* would have been inserted on an equivalent area, which could be either on distal carpals I or II, which are the most widely preserved among Sauropodomorpha [1, 65], or in the proximal surface of metacarpal I.

As in basal theropods, the action of this muscle in sauropodomorphs would have been extension of the wrist and abduction of digit I [23].

### *M*. *flexor digitorum longus* (FDL)

Like its antagonist described above, *M*. *flexor digitorum longus* is present in all reptiles, originating on the entepicondyle and the ulnar shaft and splitting at the level of the wrist and inserting on the manual digits. In lepidosaurs the proximal portion of this muscle can present different points of origin on the entepicondyle, the ulna and also the carpus [18], although the minimum configuration of humeral and ulnar origins may also exist [17]. The insertion site varies, attaching on the ventral and proximal side of terminal phalanges of all digits, as reported in *Sphenodon* [18], or on terminal phalanges of digits I and II [17].

Crocodilians retain the humeral and ulnar origin, but adding an origin from the carpus, specifically on the radiale and pisiform [18, 19]. After splitting, the insertion tendon inserts on the flexor surface of terminal phalanges of digits I-III, leaving faint ridges oriented parallel to the long axis of the phalanx; yet, Meers [19] reported an insertion on the penultimate phalanges instead of unguals. In Neornithes, the humeral head (*M*. *flexor digitorum superficialis*) inserts on the proximal phalanx of digit II, whereas the ulnar portion (*M*. *flexor digitorum profundus*) inserts on the terminal phalanx of the same digit ([18, 27], own observations).

**Fig 17:**
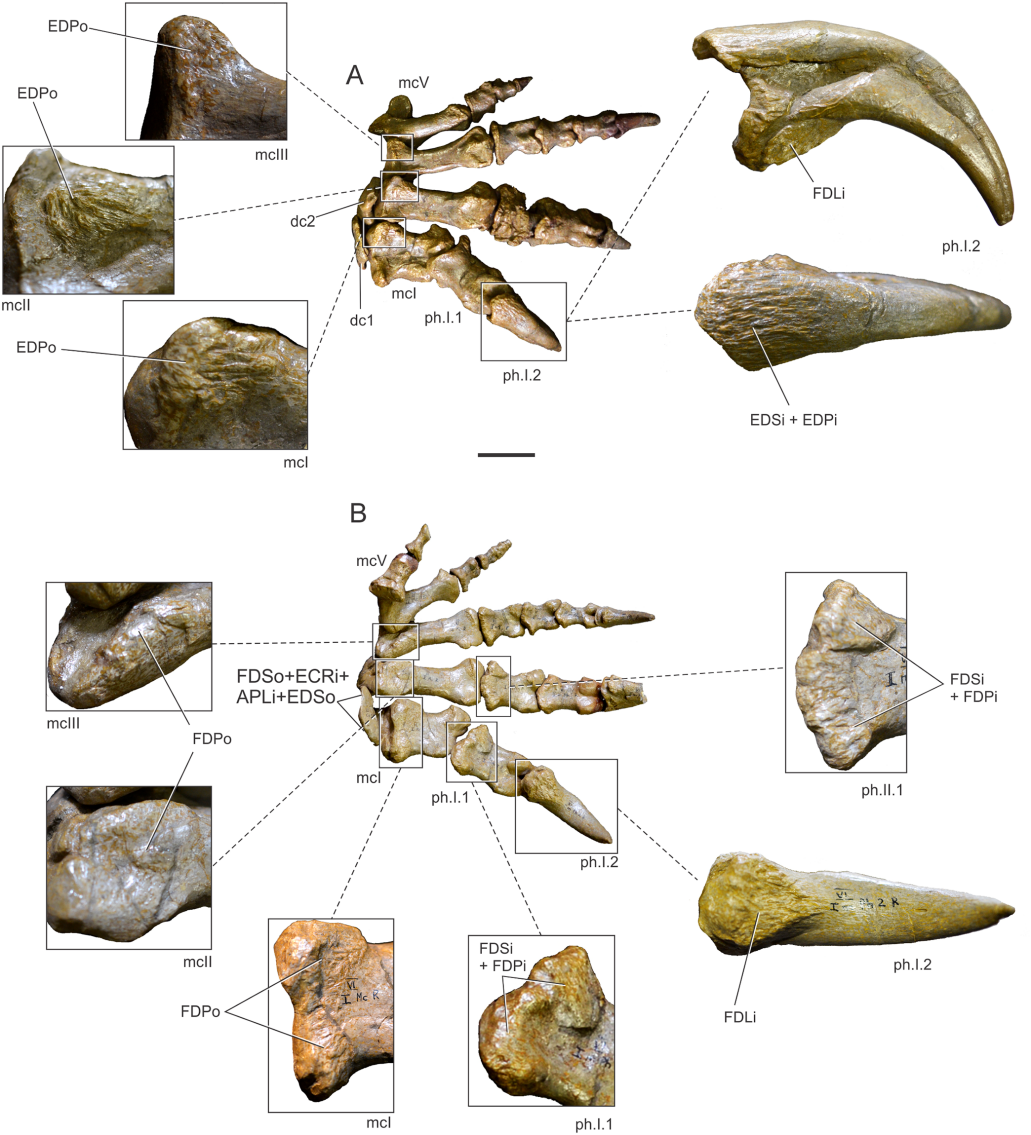
Osteological correlates of manual muscles in basal Sauropodomorpha. Left manus of *Plateosaurus engelhardti* (MB.R. 4430.173 skelett C) in dorsal (A) and palmar (B) views depicting osteological correlates of various manual muscles. Abbreviations: dc1, distal carpal one; dc2, distal carpal two; APLi, *M*. *abductor pollicis longus* insertion; ECRi, *M*. *extensor carpi radialis* insertion; EDPo, *M*. *extensor digitorum profundus* origin; EDPi, *M*. *extensor digitorum profundus* insertion; EDSi, *M*. *extensor digitorum superficialis* insertion; EDSo, *M*. *extensor digitorum superficialis* origin; FDLi, *M*. *flexor digitorum longus* insertion; FDPi, *M*. *flexor digitorum profundus* insertion; FDPo, *M*. *flexor digitorum profundus* origin; FDSi, *M*. *flexor digitorum superficialis* insertion; FDSo, *M*. *flexor digitorum superficialis* origin; mcI, metacarpal one; mcII, metacarpal two; mcIII, metacarpal three; mcV, metacarpal five; ph.I.1, first phalanx of digit one; ph.I.2, second phalanx of digit one; ph.II.1, first phalanx of digit two. Scale bar: 5 cm.

**Fig 18:**
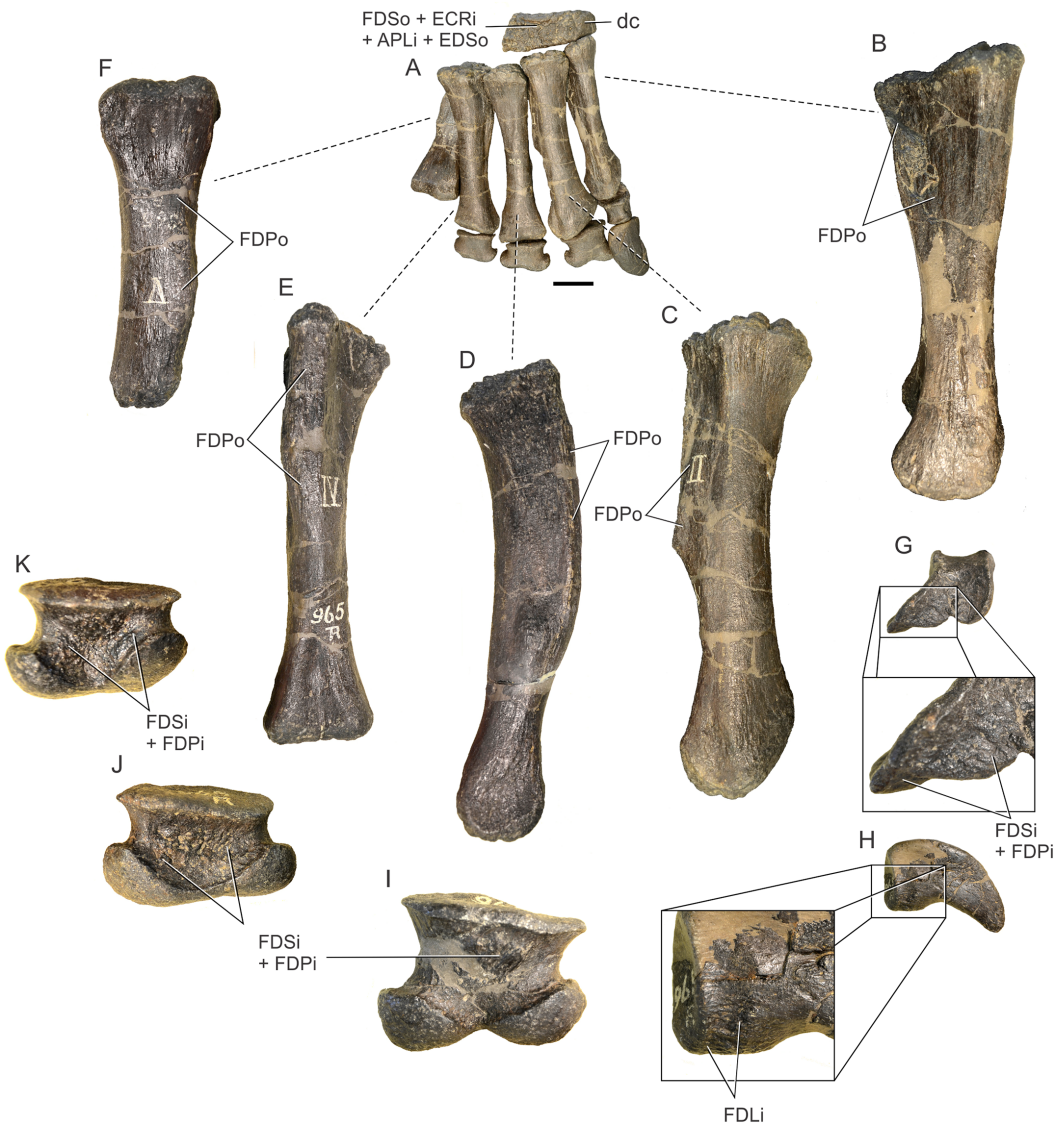
Osteological correlates of manual muscles in Sauropoda. Right manus of *Camarasaurus* sp. (AMNH 965) in dorsal view (A); metacarpal I in right lateral view (B); metacarpal II in right lateral view (C); metacarpal III in left lateral view (D); metacarpal IV in posterolateral view (E); metacarpal V in posterolateral view (F); first phalanx of digit one in right lateral view (G); second phalanx of digit one in right lateral view (H); first phalanx of digit two in palmar view (I); first phalanx of digit three in palmar view (J); first phalanx of digit four in palmar view (K). Abbreviations: dc, distal carpal; APLi, *M*. *abductor pollicis longus* insertion; ECRi, *M*. *extensor carpi radialis* insertion; EDSo, *M*. *extensor digitorum superficialis* origin; FDPi, *M*. *flexor digitorum profundus* insertion; FDPo, *M*. *flexor digitorum profundus* origin; FDSi, *M*. *flexor digitorum superficialis* insertion; FDSo, *M*. *flexor digitorum superficialis* origin; FDLi, *M*. *flexor digitorum longus* insertion. Scale bar: 5 cm.

Even though *M*. *flexor digitorum longus* is unequivocally present in Sauropodomorpha, the origin on the carpus (as in crocodiles) remains controversial since neither radiale nor pisiform is reported for this group. The ulnar origin would have been on the posterior surface, as in living archosaurs, whereas its unequivocal insertion would have been on the terminal phalanx of digit II. However, observing the pattern of insertion in both crocodilians and birds it can be noted that the anchorage of this muscle is given in the most developed digits, namely digits I-III in crocodiles, and digit II in birds. Considering that this muscle is one of the main wrist and digit flexors [19, 57], it is possible that its extent is intimately linked to digit development. It probably inserted on the first three digits in sauropodomorphs, as the pattern observed in crocodilians. In general, the proximopalmar surface of manual unguals in sauropodomorphs (at least in digits I-III) present a well-developed and striated bump, which most probably hosted the insertion tendon of *M*. *flexor digitorum longus* (*Plateosaurus engelhardti* MB.R. 4430.173.1, skelett C; *Camarasaurus* sp. AMNH 462, 965) (Figs 17 and 18).

### *Mm*. *extensores digitorum superficialis* (EDS)

This muscle complex is consistently present in all tetrapods, running along the dorsal surface of the manus, arising from different locations on the carpus or distalmost part of the antebrachium, and inserting on the ungual phalanges. In lepidosaurs, its origins can be traced either on the ulnare, intermedium, centrale or even on the distal surface of the ulna. In crocodilians the origin is on the radiale, ulnare and also the distal ulna. Meers [19] described a more complex pattern for this muscle, dividing it into five heads, one for each digit. In this sense, the portions affecting digits I-III originate from the radiale, the digit IV portion arises from both radiale and ulnare, whereas the digit V portion originates from the distal ulna and ulnare. All heads insert on the terminal phalanges, although insertions on the distal and dorsal surface of metacarpal III and IV are also reported [19].

In birds, *M*. *extensor longus digiti majoris* is regarded as the homologue of *Mm*. *extensores digitorum superficiales*. Its fleshy origin is on the posterior surface of the radius, on its distal end, and its insertion is on the dorsal and proximal surface of the second phalanx of the digit II [18, 27]. *M*. *ulnometacarpalis dorsalis* is also mentioned as part of the digit extensors [18, 23], originating tendinously from the distal end of the ulna and inserting fleshily on the dorsal surface of metacarpal III [18, 26, 27].

The presence of extensor musculature acting on the digits is clearly unequivocal in sauropodomorphs, although inferring the precise origin and insertion leads to some speculation because of the lack of proximal and intermediate elements of the wrist in this group, and also because comparisons with the modified avian morphology are difficult. In this sense, since the avian manus is extremely modified for flight, the most probable muscle morphology in sauropodomorphs should have been more similar to that of non-avian reptiles. Taking into account carpal reduction in Sauropodomorpha, the origin of *M*. *extensores digitorum superficialis* would have been from areas topologically equivalent to the ulnare and radiale, i.e. distal radius and ulna, and also the dorsal surface of distal carpal I or II. The insertion of those portions would have been on the proximodorsal surface of the distalmost phalanx on each digit, which commonly bears longitudinal striae, clearly visible in *Plateosaurus engelhardti* (MB.R. 4430.173 skelett C) (Fig 17), *Camarasaurus* sp. (AMNH 965) and *Janenschia robusta* (MB.R. 2093.5.1) (Fig 18).

### *Mm*. *extensores digitorum profundi* (EDP)

Unlike the previous muscle complex, *Mm*. *extensores digitorum profundi* originate more distally, linking the proximal metacarpus with distal phalanges along the dorsal surface of the autopodium to produce an extensor action. The proximal attachment is on both the radiale and the ulnare. This is the general scheme present in both lepidosaurs and crocodilians, although Meers [19] described some variation observed among Crocodilia.

The distal part of *M*. *extensor longus digiti majoris* and *M*. *extensor brevis alulae* are regarded as putative avian homologues of *Mm*. *extensores digitorum profundi*. The former originates on the dorsal surface of metacarpal II, inserting on the terminal phalanx of the same digit. The latter originates directly from the extensor process of the carpometacarpus and inserts on the anterodorsal surface of the alula [18, 23, 27].

In sauropodomorphs, *Mm*. *extensores digitorum profundi* most probably originated from the proximodorsal surface of all metacarpals, as observed in living archosaurs. In *Plateosaurus engelhardti* (MB.R. 4430.173 skelett C) such surface bears conspicuously striated bumps where this muscle would have originated. The insertion is on the rugose extensor surface of terminal unguals (Figs 17 and 18).

### *Mm*. *flexores digitorum superficiales* (FDS) and *profundi* (FDP)

This complex covers the ventral surface of the manus, the superficial portion originating from an aponeurosis located on the carpus (distal carpals in crocodiles) and inserting in different ways onto the distal surface of metacarpals and on proximal phalanges. The deep head originates on the distal carpals (lepidosaurs) or proximal metacarpus (crocodiles). In all cases it inserts on the base of the proximal phalanges, through a rather faint striae in analysed specimens.

There is not a consensus about the avian homologue of *Mm*. *flexores digitorum superficiales*. *M*. *flexor alulae* has recently been regarded as one of the *M*. *flexores digitorum superficiales* [23] (*contra* Remes [18]). It arises from the proximal end of the carpometacarpus, inserting on the proximal surface of the alula [18, 23, 27]. On the other hand, the muscles that fulfil the same actions that *Mm*. *flexores digitorum profundi* are *M*. *abductor digiti majoris* and *M*. *flexor digiti minoris*, which originate on each metacarpal and insert on the proximal end of the first phalanx of their corresponding digit. *M*. *adductor alulae* also has been regarded as homologue to *Mm*. *flexores digitorum profundi*, with a path similar to that of *M*. *flexor alulae* [23].

In sauropodomorphs, the unequivocal origin for *Mm*. *flexores digitorum superficiales* is on the distal carpals, most probably I and II, which are the largest. The deep head would have originated on the proximoventral surface of each metacarpal, as in living archosaurs. In this regard, the ventral surface of the proximal end in *Plateosaurus engelhardti* (MB.R. 4430.173 skelett C) presents a thickened striated surface which is the osteological correlate of the origin of this muscle. Similarly, the ventral surface of the proximal half of metacarpals in sauropods usually presents deep striae (*Camarasaurus* sp. AMNH 462, see also Tschopp *et al*. [90]; *Janenschia* MB.R. 2093.5.1; *Giraffatitan* MB.R. 2249), which probably were the osteological correlate for this muscle. Both portions would have inserted on the ventral surface of the proximal phalanges of each digit. In this sense, the proximoventral surface of proximal phalanges of *Plateosaurus engelhardti* (MB.R. 4430.173 skelett C) show marked striations, whereas in various sauropods (*Camarasaurus* sp. AMNH 462, 664, 965; *Janenschia* MB.R. 2083.3.2; *Giraffatitan* MB.R. 2728) the ventral surface of proximal phalanges is rather concave and notably striated, and would have been the insertion site for this muscle complex (Figs 17 and 18).

## Discussion

### Comparisons with previous reconstructions on dinosaur forelimb myology

Although contributions dealing with dinosaurian forelimb myology are not as abundant as ones focused on the hindlimb, there is a decent body of literature (increased during the last few years) dealing with all dinosaurian groups and thus favouring comparisons. I herein compare the musculoskeletal pattern reconstructed in this contribution with other published previous works on forelimb myology among Dinosauria, highlighting main differences and stating possible causes of those differences. For example, an interesting pattern observed in previous contributions is the scarcity of reconstructions of muscles that have an origin outside the pectoral girdle or forelimb, but an insertion on them, with the exception of *M*. *latissimus dorsi*. *Mm*. *trapezius*, *levator scapulae*, *rhomboideus* and *serratus* are reconstructed in half of the contributions listed in Table 3. A possible explanation of this pattern could be the fact that *M*. *Latissimus dorsi* is the only muscle with unambiguous osteological correlate, both visible in living archosaurs and easily trackable in dinosaurs (and in archosaurs in general).

*M*. *trapezius* is scarcely reconstructed among Dinosauria, being inferred in theropods [22, 23] and Ankylosauria [32], but absent in basal ornithischians [33]. Within Sauropodomorpha, *M*. *trapezius* is inferred for some basal forms [18] (*contra* Langer *et al*. [14]), but regarded as absent in sauropods ([15, 21]). Despite the inconsistency of the reconstruction of this muscle among dinosaurs and the lack of osteological correlates, it is reconstructed here for sauropodomorphs because its decisive positive optimization among bracket taxa. No previous reconstructions argue in favour of a secondary head inserting on the clavicles, most probably because of the lack of this bone in crocodiles and the uncertainty of its primitive presence in Dinosauria.

*Latissimus dorsi* is one of the most consistently reconstructed muscles by previous authors, not only because of its presence in both crocodilians and birds, but also as a result of the conservative osteological correlate for its insertion. Identification of this osteological correlate is rather consistent among previous interpretations, most authors agreeing on the presence of a ridge (Fig 6D in [14]; [21–23, 33]) or pit (Fig 7C, D in [15]; Fig 6A in [32]) on the posterior surface of the humerus, placed medially to the deltopectoral crest.

Although absent in birds, *M*. *levator scapulae* is reconstructed in most previous contributions on dinosaur forelimb musculature [15, 18, 22, 23, 32]. Borsuk-Bialynicka (Fig 6A, B in [15]) reported some striations on the distal anterior edge of the scapular blade as the osteological correlate of *M*. *levator scapulae* in the titanosaur *Opisthocoelicaudia* (also present in analyzed *Crocodylus* specimens), although such a correlate is not reported in any other sauropodomorph.

Although it is not possible to determine discrete portions for *M*. *pectoralis* in non-avian dinosaurs, it should be noted that sternal plates are widely reported among Sauropodomorpha, such as *Adeopapposaurus*, *Massospondylus*, *Opisthocoelicaudia*, *Bonitasaura* and *Neuquensaurus* (e.g. [1, 15, 60, 65–68, 91]), constituting the main site of anchorage of *M*. *pectoralis*. The medial surface of the deltopectoral crest in basal sauropodomorphs in general does not present any sign of striae or ridge, suggesting a fleshy insertion for this muscle, as was previously inferred for other basal sauropodomorphs [14, 18]. Cooper [30] and later Maidment and Barrett [31], however, placed the origin of *M*. *pectoralis* along the anterior rugose edge of the crest; this could also be possible considering that such a rugose and raised area is consistently present among Sauropodomorpha [62–67].

*M*. *costocoracoideus* and *M*. *sternocoracoideus* were not previously reconstructed together in a dinosaur, but instead only one of them is, mostly corresponding to *M*. *costocoracoideus* ([15, 16, 18, 32]; but see Jasinoski *et al*. [22]). Langer *et al*. [14] on the other hand, reconstructed the same muscle but opted for the avian name (i.e. *M*. *sternocoracoideus*). Insertion site of *M*. *costocoracoideus* is specified in the reconstructions of the titanosaurian sauropod *Opisthocoelicaudia* [15] and the ankylosaurian ornithischian *Euplocephalus* [30]. In the latter, the insertion is placed on the surface ventral to the glenoid, as in living crocodiles and also congruent with the interpretation proposed here for sauropodomorphs (*contra* Huene [92]). Even though the presence of a scar thought to represent an attachment for this muscle in extinct archosaurs should not be disregarded, the presence of an osteological correlate for *M*. *costocoracoideus* in dinosaurs implies an extra step of speculation since no osteological correlates are present in living archosaurs [18, 19].

Although *M*. *deltoideus scapularis* is absent in Neornithes [51], it is widely reconstructed among dinosaurs [14, 15, 18, 21–23, 30–33]. However, there is not consensus regarding the extent of its proximal attachment among dinosaurs. For example, Langer *et al*. [14] placed the origin of *M*. *deltoideus scapularis* on most of the lateral surface of the scapular blade in the basal sauropodomorph *Saturnalia*, and most previous authors also reconstructed a rather extensive origin for this muscle [23, 33]. Borsuk-Bialynicka (Fig 6C in [15]), conversely, identified a conspicuous pit on the lateral surface of the proximal scapular blade in the titanosaur sauropod *Opisthocoelicaudia*, although it is possible that this structure corresponds to a preservational or pathological artifact. Regarding the insertion, most authors favour an attachment on the proximalmost end of the thick ridge extending proximodistally and lateral to the deltopectoral crest, which also serves as the anchor for *M*. *latissimus dorsi* and corresponds to the inference in this contribution.

The origin and insertion of *M*. *deltoideus clavicularis* is rather congruent among different authors (although incongruence exist regarding the name, see Table 3), originating on the acromial area of the scapula and inserting on the lateral aspect of the deltopectoral crest of the humerus in both crocodilians and dinosaurs [18, 19, 22–24, 33]. Wilhite [21], conversely, placed the origin of this muscle on the whole lateral surface of the proximal scapula, although the pattern observed in living crocodiles and birds favour a more posterior origin on the area surrounding the acromion. Additionally, some authors proposed the presence of clavicles as the osteological correlate of *M*. *deltoideus clavicularis* [18, 23, 33]. Either way, the origin site and the line of action of this muscle would not be drastically affected by the presence of clavicles as reconstructed by Remes [18] and Yates and Vasconcelos [71]. Although the unequivocal insertion of *M*. *deltoideus clavicularis* is on the lateral surface of the deltopectoral crest [14, 23, 33], Borsuk-Bialynicka (Figs 6, 7 in[15]) and Coombs (Fig 4 in [32]) referred *M*. *deltoideus clavicularis* as *M*. *scapulohumeralis cranialis*. The latter author placed the origin site on the acromial area of the scapula, as in living archosaurs and dinosaur forelimb muscles discussed in previous contributions [14, 18, 22, 23, 33]. However, Coombs [32] placed the insertion of this muscle on a defined scar distal to the deltopectoral crest, which actually seems to be the osteological correlate of *M*. *latissimus dorsi*, not *M*. *deltoideus clavicularis*, which usually leaves no scars on living reptiles ([18, 19], own observations).

Concerning shoulder musculature, one important difference from previous contributions is the inference of *M*. *teres major*. This muscle was scarcely reconstructed within Dinosauria, with the exception of ankylosaur ornithischians [32], in which case the insertion is also shared with *M*. *latissimus dorsi*. Despite the fact that this muscle is absent in most birds and non-crocodilian reptiles, making a decisive negative assessment, the conspicuous scar present on the posterior surface of the humerus in archosaurs suggest that *M*. *teres major* could actually have been present ancestrally in archosauria.

Inferences of *Mm*. *subcoracoscapulares* among dinosaurs vary, as some authors reconstructed both scapular and coracoid heads [18, 23, 33], whereas others only inferred the scapular head [14]. The inference and reconstruction of the coracoid head (*M*. *subcoracoideus*) in this contribution responds to the fact that this muscle is primitively present in reptiles, including living birds, being the most parsimonious option the loss of this muscle in the crocodilian lineage. The muscle that Borsuk-Bialynicka (Figs 6 A and 7 A, D in [15]) figured as *M*. *subcoracoscapularis* (see also Coombs [32]), actually corresponds to *M*. *subscapularis*, as its origin is only on the medial scapula.

There is a wide consensus on reconstructing both heads of *M*. *scapulohumeralis* among Dinosauria (but see Maidment and Barrett [33]), despite the fact that crocodiles only present the posterior portion (*M*. *scapulohumeralis caudalis*, [18, 19, 57]). In spite of this consensus, the origin of *M*. *scapulohumeralis* is reconstructed from different areas, depending on author’s interpretation. Reconstructions of this complex among theropods agree in an origin on the ventral portion of the lateral surface of the scapular blade, similar to the condition reported for birds [22, 23]. Among basal sauropodomorphs, Langer *et al*. [14] placed the origin of *M*. *scapulohumeralis cranialis* together with *M*. *deltoideus clavicularis* (*M*. *deltoideus scapularis* inferior, *sensu* Langer *et al*. [14]), whereas the origin of *M*. *scapulohumeralis caudalis* is placed on the medial side of the scapular blade. In this sense, the medial side of the scapula of various basal sauropodomorphs presents a ventromedial ridge (*sensu* [75]), which most probably served as the anterior boundary of *M*. *scapulohumeralis caudalis*, as depicted by Langer *et al*. [14] and Burch [23]. These interpretations differ from those of Remes [18] for *Saturnalia*, *Efraasia* and *Antetonitrus*, in which both heads originate on the lateral side of the proximal portion of the scapula, just below the acromion. Considering the information drawn from extant taxa and information compiled by other authors, the origin of *M*. *scapulohumeralis caudalis* among dinosaurs is most probably placed on the posterior margin of the scapular blade, with the ventromedial ridge of the scapula (widely distributed among dinosaurs) acting as its medial boundary. Regarding the insertion of *Mm*. *scapulohumerales*, there is wide consensus placing it on the posterior surface of the proximal end of the humerus, via fleshy attachment, as in living archosaurs.

*Mm*. *supracoracoideus* is consistently reconstructed among dinosaurs as a single head originating from the lateral coracoid [15, 21, 22, 33] and also including part of the proximal scapula [14, 23]. As crocodiles commonly present three heads [19] and Neornithes a single one but with multiple origins, the inference of a precise origin site in extinct forms is controversial. In spite of this, the lateral coracoid is the unequivocal surface area originating *M*. *supracoracoideus*, as it occurs in both bracket taxa. However, the proximal scapular depression would have served also as an extra surface of anchorage [23] or host a completely different head [18]. A medial head seems unlikely because it occurs only in crocodiles (*M*. *supracoracoideus longus* [19]), being the lateral aspect consistently present among living reptiles (including birds). The insertion along the lateral margin of the deltopectoral crest is less speculative since this is the area reported for most living reptiles and the most cited for dinosaurs with the exception of *Massospondylus*. For this taxon Cooper [29] inferred an insertion of *M*. *supracoracoideus* on the medial aspect of the crest, on the posterior surface of the humerus, in the same topological area as the insertion of *M*. *deltoideus scapularis*.

*M*. *coracobrachialis* was reconstructed either with a single or double head among Dinosauria. Langer *et al*. [14], however, also included a third portion, *M*. *coracobrachialis longus*, which does not correspond to the homonymous muscle of lepidosaurs, as the authors placed its insertion on the posterior aspect of the proximal humerus, and not on the entepicondyle, as in lepidosaurs. Borsuk-Bialynicka (Fig 7B in [15]; see also Porpat *et al*. [76]) reconstructed two portions for this muscle, *M*. *coracobrachialis brevis* and *longus*, the latter inserting on the humeral entepicondyle as in lepidosaurs; hence, only *M*. *coracobrachialis brevis* is the one that corresponds to the archosaurian pattern, optimizing the *pars longus* as a decisive negative assessment. *M*. *coracobrachialis* was previously reconstructed in *Neuquensaurus* on a ridge rising from the anterior margin of the coracoid, as depicted by Otero ([67]; see also Huene, Pl. 9, 3a in [92]). However, this muscle presents a fleshy origin in living archosaurs [19, 27] and thus a tendinous attachment in sauropods requires more speculation. Additionally, the scar mentioned by Otero [67] is actually the origin site of *M*. *biceps brachii* (see below). Other contributions reconstructed a single head for *M*. *coracobrachialis*, although there is no consensus regarding its origin on the coracoid, which seems logical since the morphology of the dinosaurian coracoid drifted apart from that of living archosaurs. The surface between the glenoid and the ventral margin of the coracoid is the area of origin inferred for *M*. *coracobrachialis* in theropods [22, 23, 30], *Eoraptor* (Fig 64B in [93]), *Saturnalia* [14] and basal ornithischians [33]. This could also have been possible in basal sauropodomorphs, more specifically on the coracoid tubercle. However, a more lateral origin should not be ruled out because of the presence of a well-defined depression widely present among dinosaurs that would have served as attachment site for this muscle as well [18]. The broad fossa on the anterior and proximal surface of the humerus medial to the deltopectoral crest is widely accepted among authors as the insertion site of *M*. *coracobrachialis*. Mannion and Otero (Fig 6C in [74]), additionally reported a pit centrally located on such a fossa for the titanosaur *Elaltitan*. The anterior proximal fossa of the humerus is primitively well developed in crocodyliforms [94] and has also been cited as the osteological correlate for *M*. *coracobrachialis* in this group [95].

There is no consensus regarding the inference of the number of heads of *Mm*. *triceps* in previous reconstructions among Dinosauria, ranging from five to two heads. Two portions (a scapular and a humeral) were reconstructed in the titanosaur *Opisthocoelicaudia* (Fig 9A in [15]) and basal ornithischians. In the latter group, Maidment and Barrett [33] inferred a single scapular head (in the same topological position as in living crocodiles), and a humeral portion rising from the proximal and posterior surface, just lateral to the longitudinal proximal ridge. Interpretations of Maidment and Barrett [33] and also Langer *et al*. [14] reconstructed the humeral heads from small portions of the shaft. This seems unlikely, because it leaves most of the shaft with no muscle covering, a condition not observed in living archosaurs. On the other hand, five heads were inferred in *Euplocephalus*, corresponding to those described for living crocodiles [32]. Considering the phylogenetic inference and the available osteological correlates, the only unequivocal head of *Mm*. *triceps* which can be inferred in dinosaurs is the scapular one, originating along the posterior margin of the proximal portion of the scapula just above the glenoid lip, where a rugose scar were previously reported in ornithischians [32, 33], theropods [22], basal sauropodomorphs (‘supraglenoid pit’, [14]), and also in several sauropods (e.g. *Camarasaurus* sp. FMNH 25122; *Giraffatitan* HMN-SII). In spite of this, several sauropods consistently present a scapular tubercle on the posterior margin of the blade, close to the proximal end, which most probably corresponds to the scapular origin of *M*. *triceps brachii caput* scapulocoracoideum. Such tubercle is clearly observable in *Camarasaurus*, *Angolatitan*, *Daxiatitan*, *Chubutisaurus*, *Ligabuesaurus* and *Elaltitan*; hence, the presence of that portion of *Mm*. *triceps* in sauropodomorphs should not be discarded.

Most previous contributions on dinosaur forelimb myology placed the origin of *M*. *biceps brachii* just anterior to the glenoid lip of the coracoid, very close to *M*. *coracobrachialis* [14, 18, 22, 23, 30, 87, 96–99]. Nonetheless, phylogenetic inference allows reconstructing the origin of *M*. *biceps brachii* more anteriorly on the coracoid as both crocodilians and birds report an attachment site opposite to the glenoid [19, 22, 24, 28], as hypothesized also by other authors ([15, 19, 32, 33] and Fig 64C in [93]). Moreover, Meers [19] reported a prominent longitudinal scar running parallel to the shaft of the coracoid in crocodiles as the osteological correlate for this muscle. A similar scar has been demonstrated to occur among various sauropodomorphs (‘long ridge’, Fig 8G in [48]; see also Curry Rogers, Fig 33A in [62]). Despite the fact that a secondary attachment to the ulna is equivocal among dinosaurs, the presence of a scar on the proximal and anterior surface of the ulna has been reported in the basal sauropodomorph *Saturnalia* [14] and the basal theropod *Tawa* [23]. This scar has been deemed an extra insertion of *M*. *biceps brachii*, an interpretation that is not ruled out in this contribution.

The origin of *M*. *humeroradialis* is difficult to constrain in dinosaurs because of the lack of any reported scar or pit, unlike the condition in crocodiles. However, most authors did agree in an origin on the posterolateral surface of the humerus, distal to the deltopectoral crest [14, 23, 30], but Cooper (Fig 49 in [30]) placed its origin well lateral to the crest. On the other hand, Jasinoski *et al*. [22] reported low tuberosities distal to the insertion site of *M*. *deltoideus clavicularis* in some theropods. These tuberosities correspond to the origin of *M*. *humeroradialis*. Similarly, Borsuk-Bialynicka (Fig 7B in [15]) depicted a faint scar on a topological area similar to that of the crocodilian *M*. *humeroradialis*, but naming it as *M*. *brachialis inferior*. However, according to the muscle path figured by the latter author, it actually corresponds to *M*. *humeroradialis*, especially considering that the insertion site is on a proximal radial pit shared with the insertion of *M*. *biceps brachii*.

Reconstruction of the origin site for *M*. *brachialis* among dinosaurs varies within authors. Cooper [30], Langer *et al*. [14] and Maidment and Barrett [33] placed the origin of this muscle on the humerus more distally than in crocodiles. The former two authors actually place the origin site on the flexor fossa of the humerus, a hypothesis followed in this contribution because among sauropodomorphs (e.g. *Plateosaurus engelhardti* MB.R 4430.163; *Adeopapposaurus* PVSJ 610) this area presents conspicuous scars. A double insertion site for *M*. *brachialis* among dinosaurs is widely accepted [14, 18, 23, 33], although only Langer *et al*. [14] reported a scar on the anterior surface of the proximal ulna that might have been the insertion of *M*. *brachialis*.

Antebrachial and autopodial muscles have been scarcely reconstructed among dinosaurs, most previous contributions focusing mainly on shoulder and proximal forelimb inferences [14, 21, 22, 29, 30, 33]. Conversely, Borsuk-Bialynicka (Fig. 7C in [15]) reconstructed some muscles of the lower forelimb for the titanosaur *Opisthocoelicaudia*, reporting a pattern of crests and rugosities on the lateral condyle as the correlate of *M*. *extensor digitorum longus* and *extensor carpi radialis*. Nonetheless, the same author placed the insertion site for *M*. *extensor digitorum longus* on a tubercle observed on the proximolateral side of metacarpals III to V. This seems unlikely because phylogenetic inference excludes those digits.

The loss of proximal carpal elements in basal sauropodomorphs entails proposing alternative hypotheses regarding the insertion of muscles such as *Mm*. *extensor carpi radialis* and *abductor pollicis longus*, which inserts on the radiale in crocodiles and on the carpometacarpus in birds. Previous reconstructions of *M*. *extensor carpi radialis* among saurischian dinosaurs optimized insertion on the radiale—as seen in *Herrerasaurus* [43] and *Tawa* [47]—as the primitive lepidosaurian/crocodilian pattern. Santa Luca [88] reconstructed the insertion of *M*. *extensor carpi radialis* of *Heterodontosaurus tucki* on the distal and anterior end of the radius (‘radial tubercle’), an interpretation also followed by Langer *et al*. [14]. However, *Heterodontosaurus* does have a radiale bone and hence an insertion onto the distal radius is more speculative. Moreover, such an insertion would have switched the wrist extensor action to an elbow flexor. *M*. *abductor pollicis longus*, on the other hand, is reconstructed on the medial side of metacarpal I in *Eoraptor* and *Herrerasaurus* [18], as in sauropodomorphs discussed in this contribution.

*Flexor carpi ulnaris* and *extensor carpi ulnaris* have been reconstructed in both sauropodomorphs [14, 15] and theropods [18, 23]. However, in the former group, the insertion site of these muscles differs from the pattern reported for living archosaurs. In the case of *M*. *flexor carpi ulnaris*, Borsuk-Bialynicka [15] placed the attachment on the medial side of the shaft of the ulna despite the fact that the osteological correlate for the insertion of this muscle in living archosaurs are the pisiform or the ulnare. In the case of *M*. *extensor carpi ulnaris* the same author placed its insertion on metacarpal V, although in living birds metacarpal II is the insertion site of this muscle.

Inferences for the extensor and flexor musculature of the digits remains controversial among dinosaurs, and discussions dealing with this topic are scarce because of the lack of reconstructions of this muscle group (but see Remes [18]; Burch [23]). In addition, their avian homologues cannot confidently been assessed because of the drastic forelimb modification in that group. A particular challenge arises when inferring digit musculature in groups with reduced or lost manual phalanges, such as sauropods. For example, *M*. *flexor digitorum longus*, which inserts on terminal phalanges in living reptiles, would have been drastically reduced in basal macronarians, such as *Camarasaurus*, since unguals are notably reduced, and the same muscle has no osteological correlates in titanosaurs since manual phalanges are completely lost [100]. One hypothesis to explain this would be the lack of *M*. *flexor digitorum longus* in sauropods, arguing a progressive reduction of this muscle together with the reduction of manual phalanges, as one of the consequences of graviportal quadrupedalism. Despite the fact that phylogenetic inference indicates that not reconstructing this muscle in sauropods would imply a Level III inference, the loss of *M*. *flexor digitorum longus* could correspond to a sauropod specialization. An alternative hypothesis for the insertion of this muscle among sauropods would be a shifting of its osteological correlate. If we consider the trend among living archosaurs of an insertion of *M*. *flexor digitorum longus* on the most developed (and hence, more active or functional) digits (I-III in crocodiles and II in birds), the same trend could be applied for sauropods. In this sense, as phalanges are reduced in neosauropods, with extreme condition in titanosaurs, metacarpal bones become longer and add new surfaces for muscles that have no longer space among phalanges. Borsuk-Bialynicka [15] pictured this and proposed an insertion on prominences on the proximopalmar surfaces of metacarpals. Conspicuous longitudinal and scarred prominences are present on the posterior surface of metacarpal II and III in *Camarasaurus* sp. AMNH 965, *Janenschia* MB.R. 2093.5.1. and *Giraffatitan brancai* MB.R. 2249.

### Distribution of osteological correlates among Archosauria

In this section I review several osteological characters present among dinosaurs, indicating their distribution throughout Archosauria, highlighting their importance as phylogenetic characters.

#### Posterior humeral ridge

**Fig 19:**
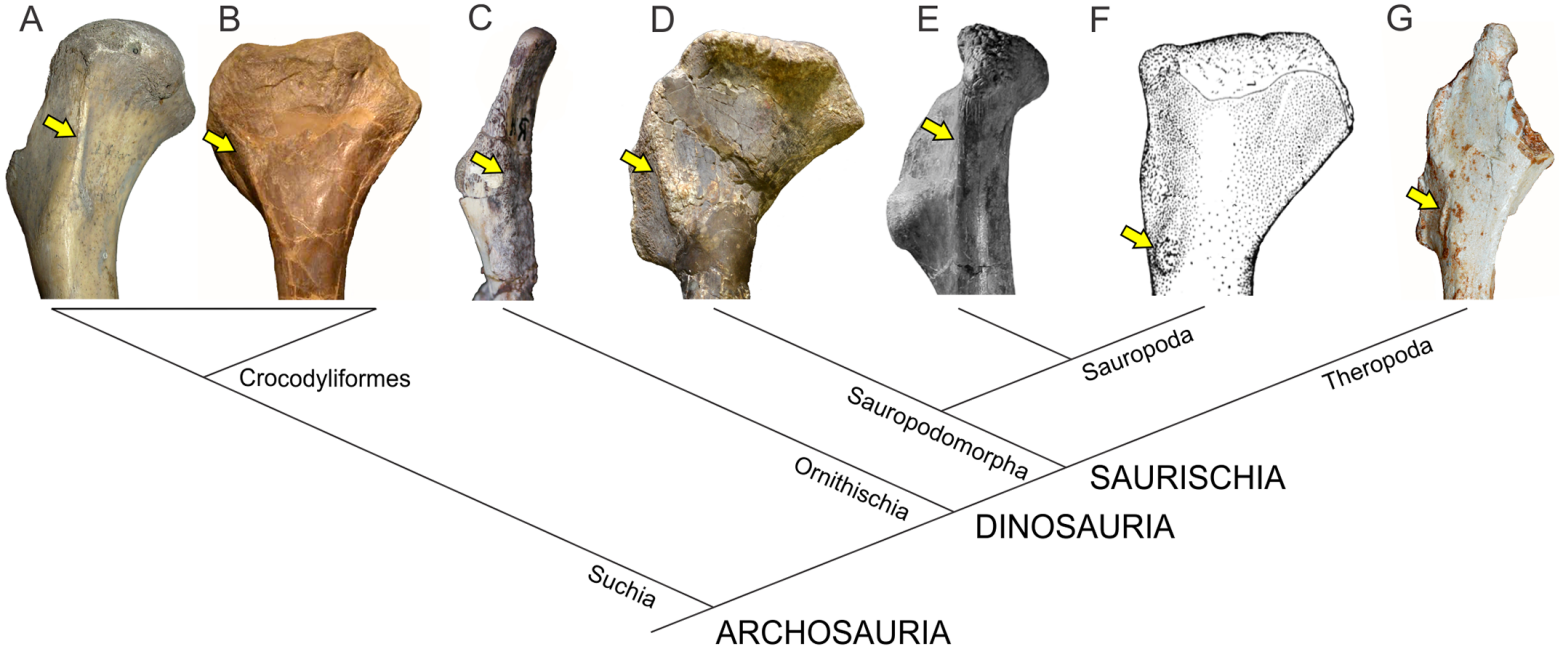
The posterior humeral ridge among Archosauria. Proximal portion of left humerus in posterior view of *Crocodylus niloticus* (A); *Batrachotomus kupferzellensis* (SMNS 80275)(B); *Scutellosaurus lawleri* (MNA Pl. 175)(C); *Plateosaurus engelhardti* (MB.R. 4404.44, skelett 25)(D); *Suuwassea emilieae* (ANS 21122)(E); *Opisthocoelicaudia skarzynski* (Reprinted from Borsuk-Bialynicka, Fig 7D in [15] under a CC BY license, with permission from Instytut Paleobiologii PAN, original copyright 1977)(F); *Segisaurus halli* (UCMP 32101)(G). The posterior humeral ridge is denoted with the arrow. (A), (C) and (F) reversed from right. Not to scale.

The proximodistally elongated ridge placed on the posterior surface of the humerus, on the proximal half of the bone and medial to the deltopectoral crest, is the most distributed humeral feature among dinosaurs discussed in this contribution. It also is primitively present within Archosauria [101]. The posterior humeral ridge is regarded as the osteological correlate for the insertion site of *M*. *latissimus dorsi* and *teres major* [18, 19, 22, 23], but adopting different morphologies and extension, depending on the group. Maidment *et al*. ([102], char. 43; see also Sereno [103], character 38.) regarded this feature as a phylogenetic character in ornithischian dinosaurs as follows: “Humerus: triceps tubercle and descending ridge posterolateral to the deltopectoral crest absent (0); present (1)”. Such descending ridge is interpreted here as the humeral posterior ridge, for which the most primitive configuration is an elongated ridge running parallel to the deltopectoral crest. That ridge can be thin and low, as observed in living crocodiles and fossil crocodyliforms such as in *Yacarerani boliviensis* (Fig 9B in [94]), or may also adopt the aspect of a rather tall ridge, like that present in the pseudosuchian *Batrachotomus kupferzellensis* (Fig 31D in [101]). Gower and Schoch [104] depicted a well-developed tubercle as the origin of the humeral head of *Mm*. *triceps brachii* in *Batrachotomus kupferzellensis*. This tubercle continues distally as the ‘supinator ridge’ (Fig 4A in [104]), interpreted here as the osteological correlate of *M*. *latissimus dorsi*. Among dinosaurs, the humeral ridge is well developed in basal sauropodomorphs (e.g. *Saturnalia* [14]; *Efraasia* SMNS 12354; *Plateosaurus engelhardti* MB.R 4404–44; *Adeopapposaurus*, PVSJ 610; *Massospondylus carinatus* SAM-K5135; *Yunnanosaurus huangi* NGMJ 004546), but it is also reported in basal ornithischians (*Scutellosaurus*, Fig 6D in [33]) and theropods (*Segisaurus halli*, Fig 6A in [105]). Among sauropods, the humeral ridge is reported in the turiasaur *Zby* [61], although it is mostly absent or reduced to a rugose pit placed more distally on the humeral shaft at the level of the distal end of the deltopectoral crest (e.g. *Opisthocoelicaudia*, Fig 7D in [15]; *Rapetosaurus* FMNH-PR 2209, *Neuquensaurus* MLP-CS 1099). A similar muscle scar is also present in the theropod *Megalosaurus bucklandii* (Fig 12C-F in [106]) and ankylosaur ornithischians (Fig 6A in [32]). The avian *margo caudalis* (Fig 4.12A in [26]) is the osteological correlate for *M*. *latissimus dorsi* in Neornithes [27, 28], maintaining the primitive morphology as a long ridge running parallel to the deltopectoral crest (Fig 19).

#### Ventromedial ridge of the scapula

**Fig 20:**
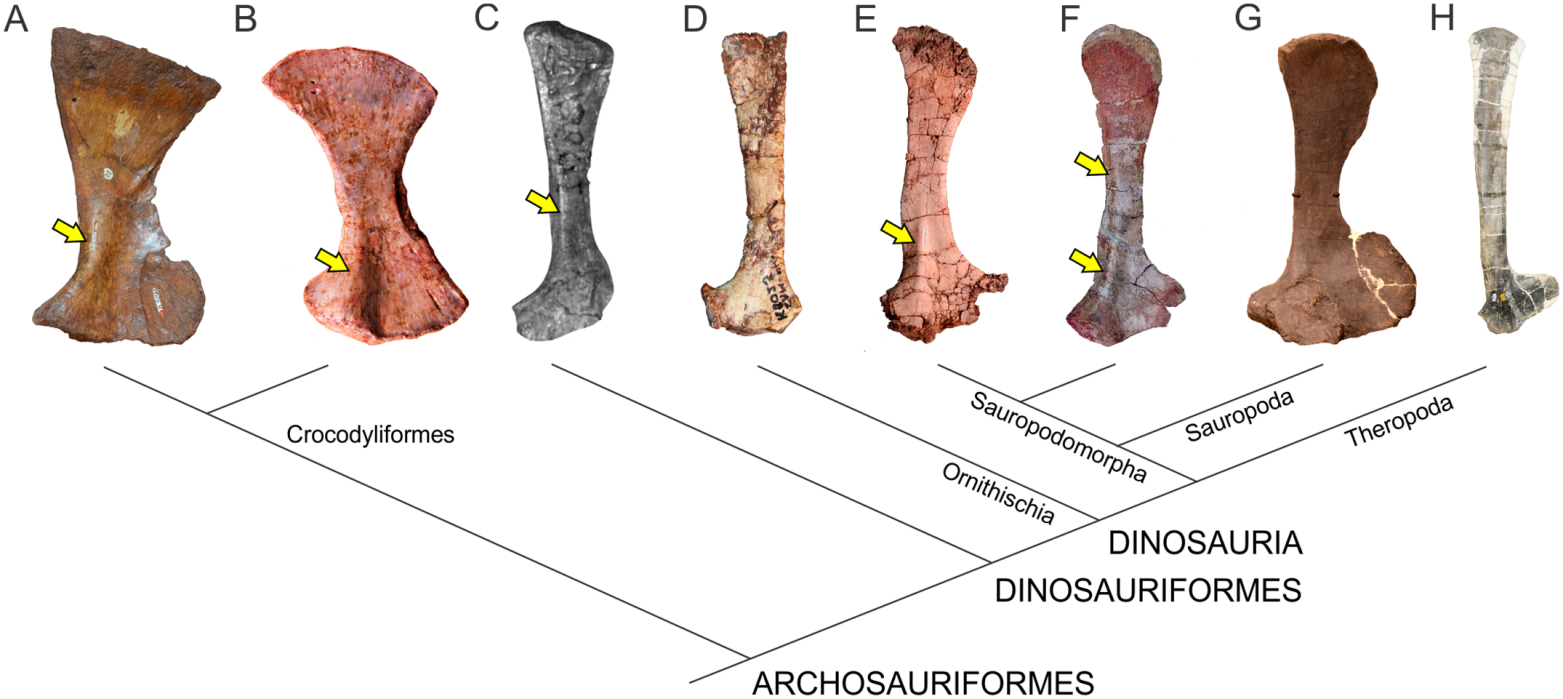
The scapular posteroventral ridge among Archosauriformes. Medial side of the scapula in *Garjania prima* (taken from Ezcurra, Fig 37A in [47]) (A); *Vancleavea campi* (PEFO 2427)(B); *Lewisuchus admixtus* (Reprinted from Bittencourt *et al*., Fig 10C in [108] under a CC BY license, with permission from Taylor and Francis, original copyright 2014)(C); *Eocursor parvus* (SAM-K8025)(D); *Sarahsaurus aurifontanalis* (TMM 33646–3.399)(E); *Mussaurus patagonicus* (MLP 68-II-27-1)(F), *Camarasaurus* sp. (FMNH 255122)(G); *Allosaurus fragilis* (UMNH-VP 10126)(H). The ventromedial ridge is denoted with the arrow. (A), (C), (D), (F) and (H) reversed from right. Not to scale.

The scapula of several archosauromorph groups presents a medial ridge or crest rising from the glenoid lip, extending distally and becoming thinner along the scapular blade, with variable extension, depending on the group. This ridge is absent in crocodile specimens analyzed here, but Meers (Fig 3 in [19]) mentioned a ‘glenoid rim’; it actually does not correspond to the ventromedial ridge here described, but only to the edge of the glenoid and does not extend to the scapular blade (*contra* Burch [23]). Although apparently absent in living crocodiles, such ridge is primitively reported in basal archosauromorphs (*Garjainia prima*, Fig 37A in [47]) and basal archosauriforms (*Vancleavea campi*, Fig 12B in [107]), extending on the first third of the scapular blade. Among basal dinosauriforms, it is absent in the basal *Silesaurus opolensis* (ZPAL Ab III-404/8), but reported in *Lewisuchus admixtus* (‘medial ridge’, Fig 10C in [108]). Among basal dinosaurs, it is reduced in *Herrerasaurus* (Fig 1B in [109]), *Tawa* [23], and absent in *Eocursor parvus* (Fig 10B in [110]). Definitely, basal sauropodomorphs show the most developed configuration of the ventromedial ridge, which presents different degrees of development among taxa, generally reaching one to two thirds of the scapular blade (e.g. *Saturnalia*, Fig 4C in [14]; *Sefapanosaurus* BP/1/7433; *Euskelosaurus* SAM-K386; *Leonerasaurus* MPEF-PV 1663). In *Mussaurus*, however, it shows the most developed configuration, extending until the distal third of the blade in MLP 68-II-27-1. In sauropods and neotheropods the ventromedial ridge is not present, and the medial surface of the scapular blade is rather flat (Fig 20). As pointed out above, the ventromedial ridge would have served as the structure separating *M*. *subscapularis* dorsally, and *M*. *scapulohumeralis caudalis* ventrally (see also Burch [23]).

#### Acromial ridge and scapular fossa

The acromial ridge and the scapular fossa are two features present in the lateral surface of the proximal expansion of the scapula. The acromial ridge arises from the distalmost tip of the acromion process and extends posteriorly along the proximal portion of the scapula with different degrees of development, whereas the scapular fossa is a depression placed just posterior to the acromial ridge, occupying most of the lateral surface of the proximal expansion of the scapula. Both structures are related to each other in the sense that the acromial ridge actually frames the fossa, turning deeper as the ridge becomes taller. However, the presence of the acromial ridge does not necessarily mean that the fossa is present too.

The acromial ridge and the scapular depression seem to be absent or poorly developed in basal archosauromorphs (Fig 36 in [47]; Fig 30 in [101]; Fig 3A in [104]; Fig 12B in [107]), and are definitively absent in the basal archosauriform *Euparkeria capensis* (SAM-K13666). Both structures are present in living crocodiles, the ridge running mostly along the dorsal margin of the acromion, not extending on the proximal expansion of the scapula; hence, the depression occupies almost the entire scapular expansion. Among dinosauromorphs the acromial ridge is thin and not excessively raised from the scapular surface but still noticeable, whereas the lateral depression is almost non-existent, as reported in the silesaurids *Silesaurus* (ZPAL AbIII-404/8) and *Sacisaurus agudoensis* [111].

**Fig 21:**
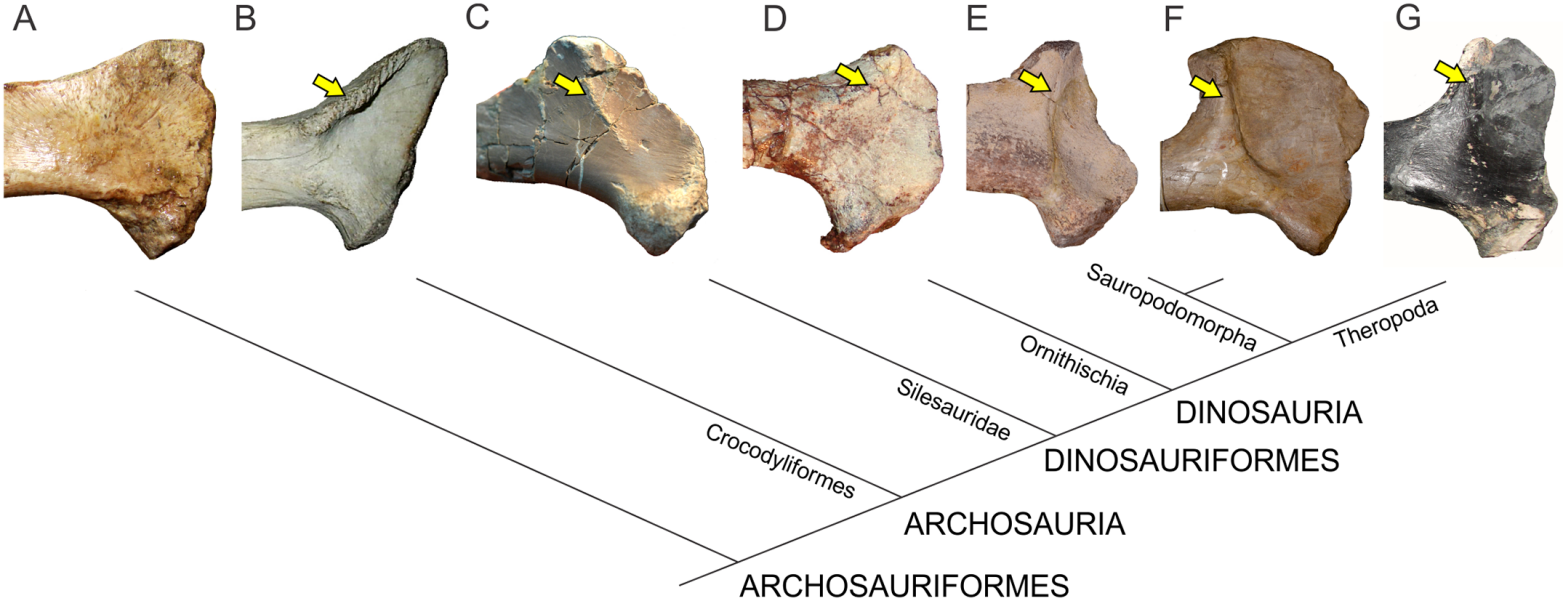
The scapular acromial ridge among Archosauriformes. Right lateral side of the scapula in *Euparkeria capensis* (SAM-K13666)(A); *Crocodylus niloticus* (B), *Silesaurus opolensis* (ZAPL Ab11/1-404/8)(C); *Eocursor parvus* (SAM-K8025) (D); *Melanorosaurus readi* (NMQR 1551)(E); *Giraffatitan brancai* (HMSII mounted skeleton)(F); *Allosaurus fragilis* (UMNH-VP 10127)(G). The acromial ridge is denoted with the arrow. (F) and (G) reversed from left. Not to scale.

Within Dinosauria, the acromial ridge becomes a raised structure widely spread among the clade and the depression becomes deep. In basal forms the ridge is rather short, not extending far from the acromion length (*Saturnalia*, Fig 4A in [14]; *Tawa* [23]; *Herrerasaurus*, Fig 1A in [109]; *Eocursor*, Fig 10A in [110]), whereas in the rest of dinosaurian groups it is a well-developed raised ridge, mostly extending far from the acromion process and framing a well-developed fossa (e.g. Fig 16.8A in [1]; Fig 80C in [87]; Fig 3A in [99]; Fig 20A in [112]). In ankylosaurs, however, the ridge becomes a stout prominence restricted to the acromial tip (Fig 3B in [32]). In sauropods the acromial ridge is extremely developed, becoming notably thick and extending half way across the proximal scapular expansion, whereas the associated fossa is clearly framed by it and reaching the greatest depth among dinosaurs (e.g. *Antetonitrus*, BP/1/4952; *Camarasaurus* sp. FMNH 25122, Fig 75 in [71]; *Rapetosaurus* FMNH-PR 2209; *Elaltitan* PVL 4628, Fig 6A, B in [74]) (Fig 21).

This acromial ridge would have served as a dorsal boundary of *M*. *supracoracoideus*, whereas the scapular fossa is actually regarded as the osteological correlate for this muscle ([18, 19, 23] *contra* [21]). It is interesting to note that theropod dinosaurs exhibit a notable dorsoventral reduction of the proximal expansion of the scapula, particularly of the surface hosting the scapular depression. Such reduction is extreme in *Majungasaurus crenatissimus*, which displays atrophied forelimbs [99]. In the opposite way, sauropod dinosaurs present a great development of the proximal expansion of the scapula, coupled with a deep lateral fossa. These contrasting patterns of osteological correlates between theropods and sauropods can be the result of different muscular morphology between those saurischian groups. In this sense, the atrophied forelimbs of *Majungasaurus* would have been related to a weak development of part of its forelimb muscles, such as *M*. *supracoracoideus*, whereas the graviportal sauropods would have required a more massive forelimb musculature, reflected in more expanded and deep scapular surfaces.

#### Biceps ridge of the coracoid

The osteological correlate for *M*. *biceps brachii* in basal archosauriforms is regarded as a tubercle placed on the posterolateral surface of the coracoid, below the glenoid (‘biceps tubercle’, Fig 36 in [47]; Fig 30 in [101]). In this sense, the presence of such swollen tubercle was previously used as a character state widely present among Archosauromorpha ([47] character 401; [101] character 225). However, as previously mentioned in this contribution, the tubercle present ventrally to the glenoid would actually correspond to an attachment portion of *M*. *coracobrachialis*, according to the phylogenetic inference provided by extant archosaurs [18, 19]. Thus, the tendinous origin of *M*. *biceps brachii* in living archosaurs corresponds to a longitudinal scar running parallel to the shaft of the coracoid, opposite to the glenoid [19, 22], whereas a topologically similar tubercle is described for some birds [28]. Therefore, a topologically similar area would be expected to be the origin site of *M*. *biceps brachii* in extinct archosaurs as well. The biceps tubercle of the coracoid seems to be absent in basal archosauromorphs, basal archosauriforms and basal dinosauromorphs [47, 101]. In theropod dinosaurs the coracoid biceps origin has been regarded as a raised tubercle consistently placed between the glenoid and the coracoid foramen (e.g. Fig 83 in [87]; Fig 3A in [99]; Pl. 41A in [113]). Among sauropodomorphs, the osteological correlate of *M*. *biceps* proposed in this contribution is consistently placed on the anteroventral edge of the coracoid, perpendicular to its margin. Such osteological correlate takes the form of an elongated scar in most taxa (e.g. *Sefapanosaurus* BP/1/7424, *Antetonitrus* BP/1/4956, *Suuwassea*, Fig 1.2 in [83]; *Giraffatitan* HMN SII mounted skeleton; *Rapetosaurus* FMNH-PR 2209 mounted skeleton), although in derived titanosaurs the biceps scar actually becomes a thick ridge (e.g. *Opisthocoelicaudia* ZPAL MgD-I/25c; *Saltasaurus* PVL 4017–101; *Neuquensaurus* MLP-CS 1096)(Fig 11). The pattern present in sauropodomorphs contrasts with the origin site previously inferred for this muscle in theropods. At this point it is difficult to choose the most plausible osteological correlate for *M*. *biceps* between theropods and sauropodomorphs, especially considering the morphological disparity between the crocodilian and dinosaurian coracoid, which precludes a precise correspondence of osteological correlates. Considering that *M*. *biceps brachii* is the only muscle with tendinous attachment (leaving a scar) on the lateral surface of the coracoid in both living archosaurs, it is plausible that the scar present in the lateral coracoid of both saurischian groups correspond to the origin site of *M*. *biceps brachii*, reflecting different character states.

#### Radial intermuscular lines and ulnar ridge

**Fig 22:**
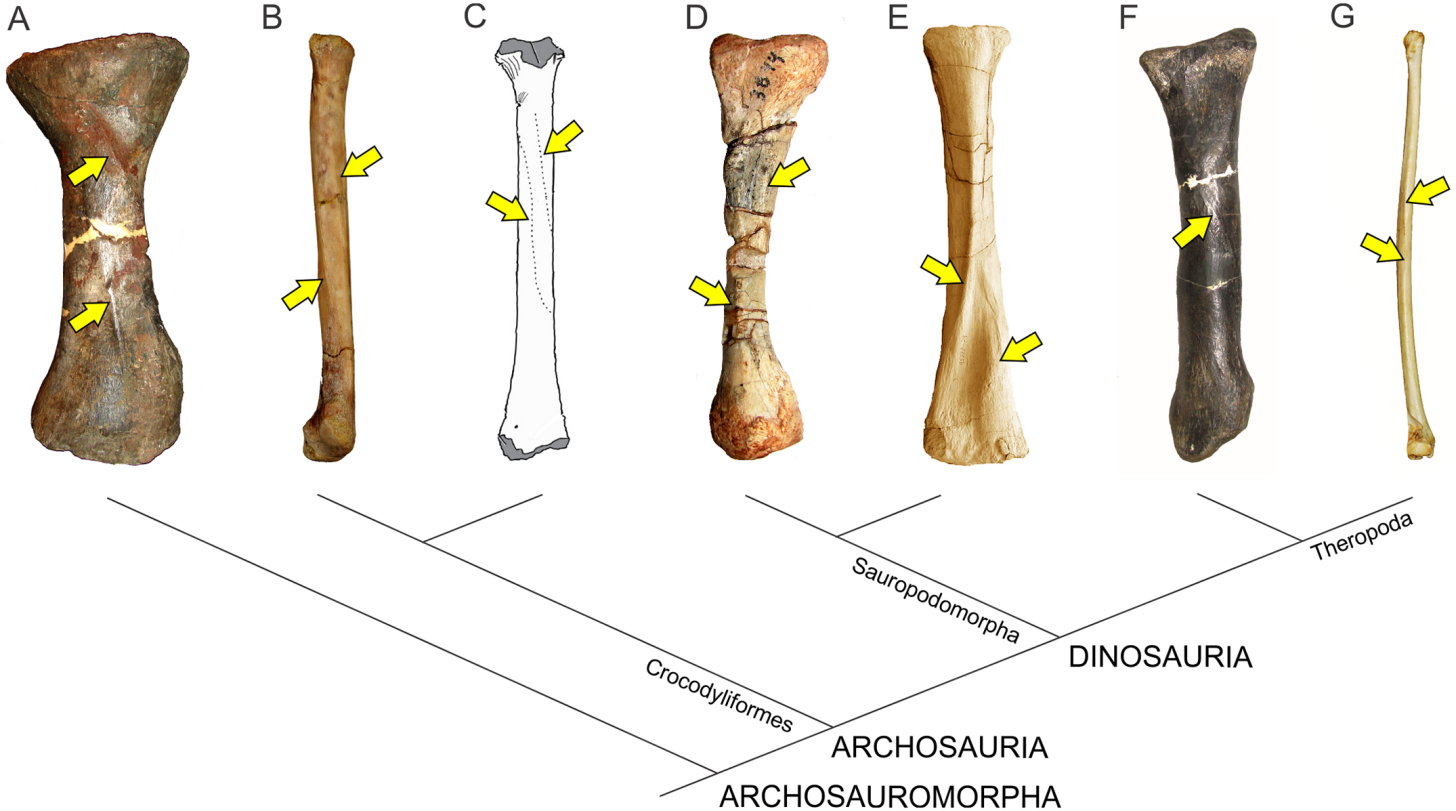
The radial intermuscular lineae among Archosauromorpha. Ulnar side of the radius in *Erythrosuchus africanus* (SAM-K905)(A); *Simosuchus clarki* (UA 8679)(B); *Pissarrachampsa sera* (taken from Godoy *et al*., Fig 4F in [116])(C); *Saturnalia tupiniquim* (MCP 3844-PV)(D); *Rapetosaurus krausei* (FMNH-PR 2209)(E); *Allosaurus fragilis* (UMNH-VP CR-5)(F); *Sarcoramphus papa* (MLP-O 14362)(G). The *intermuscular lineae* are denoted with the arrow. Not to scale.

The radius of some groups of archosaurs presents thin longitudinal ridges (intermuscular lines) running parallel to the radial shaft and placed on the ulnar face. The placement of such *lineae* is congruent with the insertion site of *Mm*. *pronator teres* and *quadratus* (‘arista longitudinal’, [92]; ‘interosseous ridge’, [15, 67]). The *lineae intermuscularis* on the radius are probably absent in living crocodiles, but primitively present in basal archosauromorphs (e.g. *Trilophosaurus buettneri*, [114]), basal archosauriforms (*Erythrosuchus*, [47]) and they were reported in the extinct crocodyliforms *Notosuchus terrestris* (Fig 13 in [115]), *Pissarrachampsa sera* (Fig 4F in [116]) and *Simosuchus clarki* (Fig 11D in [95]). Among basal dinosaurs, *Herrerasaurus* (PVSJ 373, Figs 7B, 8 in [109]) and *Eoraptor* (Fig 65B in[93]) present a faint single ridge crossing the biceps tubercle, which might be considered an intermuscular line. Radial intermuscular lines are also reported in neotheropods [87, 99]. In the case of *Tyrannosaurus rex* (Fig 86B, E in [87]), such ridges are regarded as the origin site for digit flexors and extensors, although phylogenetic inference indicates that such area is actually for the insertion of *M*. *pronator teres*. Radial intermuscular lines are retained by living birds [26] (Fig 22).

Among basal sauropodomorphs, radial intermuscular lines are not widely distributed, although *Saturnalia* shows three intermuscular lines, but two are present on the ulnar surface (Fig 9 in [14]); a similar ridge is present in *Plateosaurus engelhardti* (MB.R. 4404.46). Among sauropods, on the other hand, radial intermuscular lines are more widely reported as two relatively thick and parallel ridges (Fig 8C in [15]; Fig 5A–C in [67]; Fig 12E in [77]), although *Rapetosaurus* shows only one (Fig 36C, D in [62]).

The ulnar ridge, a longitudinal crest running parallel to the ulnar shaft, is interpreted in this contribution as the osteological correlate of *M*. *pronator quadratus*. Unlike the radial intermuscular lines, the ulnar ridge is restricted among archosaurs, being primitively present in *Heterodontosaurus* [88], *Sanjuansaurus* (Fig 6E in [89]) and *Herrerasaurus* (PVSJ 373, Fig 7B, 8 in [109]) as a thin ridge arising from the ulnar midshaft. It is also present in some theropods taking the form of a thick ridge (Fig 86B in [87], E; Figs 6, 7 in [99], ‘interosseous ridge’). It seems to be absent in basal sauropodomorphs. It reappears among sauropods, where it becomes a notably thick ridge present in several camarasauromorphs (*Camarasaurus*, *Rapetosaurus*, *Giraffatitan*, *Bonitasaura*, *Opisthocoelicaudia* and *Neuquensaurus australis*).

#### Biceps tubercle of the radius

As the name implies, the biceps tubercle, present on the proximomedial aspect of the radius, is regarded as the insertion site for the homonymous muscle and also the *M*. *humeroradialis*. The osteological correlate for *M*. *biceps brachii* is primitively present in fossil crocodyliforms as low bump or even just rugosities (Fig 10E in [94]; Fig 11A in [95]; Fig 4H in [116]), whereas in dinosaurs it become more developed. Despite the fact that this feature is widely present among dinosaurs, it only recently has been used as a phylogenetic character in the context of sauropodomorph phylogeny ([48] character 368). The biceps tubercle is primitively present in *Herrerasaurus* (Fig 7A in [109]) and *Eoraptor* (Fig 65B in [93]), is reported in theropods [87, 117] and ornithischians [32] and is widely present among sauropodomorphs, being present in *Plateosaurus engelhardti* (MB.R. 4404 skelett 25), *Rueheleia* (MB.R. 4718), *Sefapanosaurus* (BP/1/7435), *Aardonyx* (BP/1/5379), *Antetonitrus* (BP/1/4952), *Giraffatitan* (HMN SII mounted skeleton), *Elaltitan* (PVL 4628), *Neuquensaurus* (MLP-CS 1169) and *Opisthocoelicaudia* (Fig 8B in [15]).

### Muscle morphology and neck elongation in Sauropodomorpha

One of the most interesting differences observed between basal sauropodomorphs and sauropods is the development of the neck. Although neck elongation is a feature that distinguishes Sauropodomorpha from other dinosaurian groups, the way in which sauropods achieved extreme neck elongation is unique among dinosaurs and also one of the sauropodomorph evolution novelties that remain poorly understood.

Neck elongation in sauropods involved a rearrangement of the bony structure in order to gain strength with the minimum weight increase. Thus, the complex system of vertebral laminae was proposed as a solution to mitigate the body mass requirements, acting as structural elements which reduce weight on the one hand [118–120], but also as the osteological correlates associated with a complex system of air sacs [121, 122–125]. However, how muscle morphology accommodates to that structural change has received little attention [16].

Sauropod dinosaurs display stout cervical vertebrae with a well-developed diapophysis-parapophysis complex (‘*ansa costo-transversaria*’ *sensu* Wedel and Sanders [126]) along the whole neck extension. Conversely, basal sauropodomorphs display slender cervical vertebrae with weak diapophyses and almost non-existent parapophyses until the fifth or sixth element, in which diapophyses become longer and thicker (Fig 8). *Mm*. *levator scapulae* and *serratus profundus*, both attaching to the diapophysis-parapophysis complex most probably would have been well developed in sauropods, extending well anteriorly on the sauropod neck (see also Schwarz *et al*., Fig 7 in [16]). In basal sauropodomorphs, however, it is expected that those muscles would have been less developed, extending until cervical vertebrae five or six, where the diapophysis-parapophysis complex becomes stouter and with a larger surface to host the attachment of such musculature. In the same way, *M*. *trapezius*, which plesiomorphically extends anteriorly over the vertebral column involving cervical vertebrae and the scapular girdle, would have been notably enlarged in sauropods, considering the presence of up to 17 cervical vertebrae [56]. Hence, the fan-shaped morphology described for *M*. *trapezius* in living crocodiles is expected to be modified in Sauropodomorpha, most probably consisting of an elongated cervical and sheet-like scapular portion.

In summary, neck elongation along sauropodomorph evolution would have involved not only the development of a complex system of laminae and fossae, but also the rearranging of the neck musculature attached to the pectoral girdle in order to support extreme neck elongation, as previously pictured out by Schwarz *et al*. [16].

## Conclusions

Osteological correlates constitute (ideally) the keystone for muscle reconstruction using the Extant Phylogenetic Bracket approach [36, 41]. Most previous contributions on dinosaur myology focused on the reconstruction of the muscle arrangement of the animal, identifying areas of origin and insertion of a specific muscle with its corresponding Level of Inference, with the ultimate goal of elucidating which muscles would have been present in a particular fossil taxon. In this sense, a correct identification of an osteological correlate of a specific muscle is the first (but not only) step towards an accurate paleobiological study focused on muscle arrangement and morphology.

This contribution presented the forelimb musculature of sauropodomorph dinosaurs, including the complete muscular arrangement for basal sauropodomorphs. Although forelimb muscular inferences were previously given for dinosaurs (e.g. [15, 18, 23, 33], this study presents a comprehensive reconstruction of most muscles of the forelimb, including those originating on cervical vertebrae, but also distal muscles attached to the autopodium, usually ignored in previous contributions. This contribution demonstrates that some osteological correlates present in the forelimb of sauropodomorph dinosaurs actually characterizes more inclusive groups (e.g. posterior humeral ridge, ventromedial scapular ridge, radial intermuscular lines), whereas others are only reported within dinosaurs (e.g., biceps ridge of the coracoid and radius). Hence, this contribution provides a complete guide for osteological correlate recognition, making it possible to track them along sauropodomorph evolution, and also permitting comparisons with other dinosaurs, basal dinosauromorphs and basal archosauromorphs. Knowing how such correlates change among and within groups will ultimately allow the researcher to elucidate the location, morphology and path of muscles associated with that osteological feature, adding invaluable rigour to future studies based on myological reconstructions such as analysis of moment arms or character optimization in the context of phylogenetic analyses.

## References

[1] Galton PM, Upchurch P. Prosauropoda In: Weishampel DB, Dodson P, Osmólska H. The Dinosauria. Berkeley: University of California Press; 2004 pp. 232–258.

[2] Smith ND, Pol D. Anatomy of a basal sauropodomorph dinosaur from the Early Jurassic Hanson Formation of Antarctica. Acta Palaeontologica Polonica. 2007; 52: 657–674.

[3] Cerda IA, Carabajal AP, Salgado L, Coria RA, Reguero MA, Tambussi CP, et al The first record of a sauropod dinosaur from Antarctica. Naturwissenschaften. 2012a; 99: 83–87. 22173579 10.1007/s00114-011-0869-x

[4] Mannion PD, Upchurch P, Carrano MT, Barrett PM. Testing the effect of the rock record on diversity: a multidisciplinary approach to elucidating the generic richness of sauropodomorph dinosaurs through time. Biological Reviews. 2010; 86: 157–181. 10.1111/j.1469-185X.2010.00139.x 20412186

[5] Bonnan MF. The evolution of manus shape in sauropod dinosaurs: implications for fuctional morphology, forelimb orientation, and phylogeny. Journal of Vertebrate Paleontology. 2003; 23: 595–613.

[6] Carrano MT. The evolution of Sauropod locomotion: Morphological diversity of a secondarily quadrupedal radiation In: Curry Rogers KA, Wilson JA, editors. The Sauropods: Evolution and Paleobiology. Berkeley: University of California Press; 2005 pp. 229–249.

[7] Bonnan MF, Yates AM. A new description of the forelimb of the basal sauropodomorph *Melanorosaurus*: implications for the evolution of pronation, manus shape, and quadrupedalism in sauropod dinosaurs. In: Barrett P.M. and Batten D. J. (Eds.), Evolution and Paleobiology of Early Sauropodomorph Dinosaurs. Special Papers in Palaeontology. 2007; 77: 157–168.

[8] Yates AM, Bonnan MF, Neveling J, Chinsamy A, Blackbeard MG. A new transitional sauropodomorph dinosaur from the Early Jurassic of South Africa and the evolution of sauropod feeding and quadrupedalism. Proceedings of the Royal Society B. 2010; 277: 787–794. doi: 10.1098/rspb.2009.1440 19906674 10.1098/rspb.2009.1440 PMC2842739

[9] Otero A, Allen V, Pol D, Hutchinson JR. Forelimb muscle and joint actions in Archosauria: insights from *Crocodylus johnstoni* (Pseudosuchia) and *Mussaurus patagonicus* (Sauropodomorpha). PeerJ. 2017; 5:e3976; doi: 10.7717/peerj.3976 29188140 10.7717/peerj.3976 PMC5703147

[10] Bonaparte JF, Coria RA. Un nuevo y gigantesco saurópodo titanosaurio de la Formación Rio Limay (Albiano-Cenomaniano) de la Provincia del Neuquén, Argentina. Ameghiniana. 1993; 30: 271–282.

[11] Bates KT, Mannion PD, Falkingham P, Brusatte SL, Hutchinson JR, Otero, et al Temporal and phylogenetic evolution of the sauropod dinosaur body plan. Royal Society Open Science. 2016; 3: 150636 doi: 10.1098/rsos.150636 27069652 10.1098/rsos.150636 PMC4821263

[12] González Riga BJ, Lamanna MC, Ortiz David LD, Calvo JO, Coria JP. A gigantic new dinosaur from Argentina and the evolution of the sauropod hind foot. Scientific Reports. 2016; 6: 19165 doi: 10.1038/srep19165 26777391 10.1038/srep19165 PMC4725985

[13] Carballido JL, Pol D, Otero A, Cerda IA, Salgado L, Garrido AC, et al A new giant titanosaur sheds light on body mass evolution among sauropod dinosaurs. Proceedings of The Royal Society B. 2017; 284: 20171219. 10.1098/rspb.2017.1219 PMC5563814 28794222

[14] Langer MC, Franca MAG, Gabriel S. The pectoral girdle and forelimb anatomy of the stem sauropodomorph *Saturnalia tupiniquim* (Late Triassic, Brazil). Special Papers in Palaeontology. 2007; 77: 113–137.

[15] Borsuk-Bialynicka M. A new camarasaurid sauropod *Opisthocoelicaudia skarzynskii*, gen. n., sp. n. from the Upper Cretaceous of Mongolia. Palaeontologia Polonica. 1977; 37: 1–64.

[16] Schwarz D, Frey E, Meyer CA. Novel Reconstruction of the Orientation of the Pectoral Girdle in Sauropods. The Anatomical Record. 2007; 290: 32–47. doi: 10.1002/ar.20405 17441196 10.1002/ar.20405

[17] Zaaf A, Herrel A, Aerts P, De Vree F. Morphology and morphometrics of the appendicular musculature in geckoes with different locomotor habits (Lepidosauria). Zoomorphology. 1999: 119: 9–22.

[18] Remes K. Evolution of the pectoral girdle and forelimb in Sauropodomorpha (Dinosauria, Saurischia): Osteology, myology and function. Unpublished D. Phil. Thesis, Universität München. 2008.

[19] Meers MB. Crocodylian forelimb musculature and its relevance to Archosauria. The Anatomical Record Part A. 2003; 274: 892–916. 10.1002/ar.a.10097 12973714

[20] Reese AM. The Alligator and Its Allies. New York: G.P. Putnam 1915.

[21] Wilhite R. Biomechanical reconstruction of the appendicular skeleton in three North American Jurassic Sauropods. Ph. D. dissertation. Louisiana State University, Baton Rouge. 2003. pp. 185.

[22] Jasinoski SC, Russell AP, Currie PJ. An integrative phylogenetic and extrapolatory approach to the reconstruction of dromaeosaur (Theropoda: Eumaniraptora) shoulder musculature. Zoological Journal of The Linnean Society. 2006; 146: 301–344.

[23] Burch SH. Complete forelimb myology of the basal theropod dinosaur *Tawa hallae* based on a novel robust muscle reconstruction method. Journal of Anatomy. 2014; 225: 271–297. doi: 10.1111/joa.12216 25040486 10.1111/joa.12216 PMC4166969

[24] Suzuki D, Hayashi S. Myology of crocodiles II: Pectoral girdle and forelimb. The Palaeontological Society of Japan. 2010; 87: 83–102.

[25] Klinkhamer AJ, Wilhite DR, White MA, Wroe S. Digital dissection and three- dimensional interactive models of limb musculature in the Australian estuarine crocodile (*Crocodylus porosus*). PLoS ONE. 2017; 12(4): e0175079 doi: 10.1371/journal.pone.0175079 28384201 10.1371/journal.pone.0175079 PMC5383063

[26] Vanden Berge JC, Zweers GA. Myologia In: Baumel JJ, King AS, Breazile JE, Evans HE, Vanden Verge JC, editors. Handbook of Avian Anatomy: Nomina Anatomica Avium. Massachusetts: Publications of the Nutall Ornithological Club 23 1993 pp. 189–250.

[27] Mckitrick MC. Forelimb mycology of loons (Gaviiformes), with comments on the relationship of loons and tubenoses (Procellariiformes). Zoological Journal of the Linnean Society. 1991; 102: 115–152.

[28] Meyers RA. Morphology of the shoulder musculature of the American kestrel, *Falco sparverius* (Aves), with implications for gliding flight. Zoomorphology. 1992; 112: 91–103.

[29] Cooper MR. The prosauropod Dinosaur *Massospondylus carinatus* Owen from Zimbabwe: Its biology, mode of life and phylogenetic significance. Occasional Papers of the Natural Museum of Rhodesia, B, Natural Sciences. 1981; 6: 689–840.

[30] Nicholls EL, Russell AP. Structure and function of the pectoral girdle and forelimb of *Struthiomimus altus* (Theropoda: Ornithomimidae). Palaeontology. 1985; 28: 643–677.

[31] Burch SH. Myology of the forelimb of *Majungasaurus crenatissimus* (Theropoda, Abelisauridae) and the morphological consequences of extreme limb reduction. Journal of Anatomy. 2017; doi: 10.1111/joa.12660 28762500 10.1111/joa.12660 PMC5603782

[32] Coombs WP Jr. Forelimb muscles of the Ankylosauria (Reptilia, Ornithischia). Journal of Paleontology. 1978; 52: 642–657.

[33] Maidment SCR, Barrett PM. The locomotor musculature of basal ornithischian dinosaurs. Journal of Vertebrate Paleontology. 2011; 31: 1265–1291.

[34] Romer AS. Crocodilian pelvic muscles and their avian and reptilian homologues. Bulletin of the American Museum of Natural History. 1923; 48: 533–552.

[35] Bryant HN, Russell AP. The role of phylogenetic analysis in the inference of unpreserved attributes of extinct taxa. Philosophical Transactions of the Royal Society of London B. 1992; 337: 405–418.

[36] Witmer LM. The extant phylogenetic bracket and the importance of reconstructing soft tissues in fossils In: Thomason JJ, editor. Functional morphology in Vertebrate Paleontology. Cambridge: Cambridge University Press; 1995 pp. 19–33.

[37] Ostrom JH. The pectoral girdle and forelimb function of *Deinonychus* (Reptilia: Saurischia): a correction. Postilla. 1974; 165: 10–14.

[38] Gatesy SM. Hind limb movements of the American alligator (*Alligator mississippiensis*) and postural grades. Journal of Zoology. 1991; 224: 577–588.

[39] Hutchinson JR, Gatesy SM. Adductors, abductors, and the evolution of archosaur locomotion. Paleobiology. 2000; 26: 734–751.

[40] Bates KT, Schachner ER. Disparity and convergence in bipedal archosaur locomotion. Journal of the Royal Society Interface. 2012; 9: 1339–1353. 10.1098/rsif.2011.0687 PMC3350733 22112652

[41] Witmer LM. The evolution of the antorbital cavity in archosaurs: a study in soft-tissue reconstruction in the fossil record with an analysis of the function of pneumaticity. Society of Vertebrate Paleontology Memoir. 1997; 3: 1–73.

[42] Carrano MT, Hutchinson JR. Pelvic and Hindlimb musculature of *Tyrannosaurus rex* (Dinosauria: Theropoda). Journal of Morphology. 2002; 253: 207–228. doi: 10.1002/jmor.10018 12125061 10.1002/jmor.10018

[43] Carrano MT. Locomotion in non-avian dinosaurs: integrating data from hindlimb kinematics, in vivo strains, and bone morphology. Paleobiology. 1998; 24, 450–469.

[44] Carrano MT. Homoplasy and the evolution of dinosaur locomotion. Paleobiology. 2000; 26: 489–512.

[45] Chiappe LM. The first 85 million years of avian evolution. Nature. 1995; 378: 349–355.

[46] Benton MJ. Origin and relationships of Dinosauria In: Weishampel DB, Dodson P, Osmólska H. The Dinosauria. Berkeley: University of California Press; 2004 pp. 7–19.

[47] Ezcurra MD. The phylogenetic relationships of basal archosauromorphs, with an emphasis on the systematics of proterosuchian archosauriforms. PeerJ. 2016; 4: e1778 doi: 10.7717/peerj.1778 27162705 10.7717/peerj.1778 PMC4860341

[48] Otero A, Krupandan E, Pol D, Chinsamy A, Choiniere J. A New Basal Sauropodiform from South Africa and the phylogenetic relationships of basal sauropodomorphs. Zoological Journal of the Linnean Society. 2015; 174: 589–634.

[49] Carballido JL, Pol D, Cerda I, Salgado L. The osteology of *Chubutisaurus insignis del Corro*, 1975 (Dinosauria: Neosauropoda) from the ‘middle’ Cretaceous of central Patagonia, Argentina. Journal of Vertebrate Paleontology. 2011; 31: 1: 93–110.

[50] Harris JD. Confusing dinosaurs with mammals: tetrapod phylogenetics and anatomical terminology in the world of homology. The Anatomical Record. 2004; 281A: 1240–1246. 10.1002/ar.a.20078 15384041

[51] Wilson JA. Anatomical nomenclature of fossil vertebrates: standardized terms or ‘Lingua franca’? Journal of Vertebrate Paleontology. 2006; 26: 511–518.

[52] Parrish JM. The origin of crocodilian locomotion. Paleobiology. 1987; 13: 396–414.

[53] Blob RW, Biewener AA. In vivo locomotor strain in the hindlimb bones of *Alligator mssissippiensi*s and *Iguana iguana*: implications for the evolution of limb bone safety factor and non-sprawling limb posture. The Journal of Experimental Biology. 1999; 202: 1023–1146. 10101104 10.1242/jeb.202.9.1023

[54] Blob RW. Evolution of hindlimb posture in nonmammalian therapsids: biomechanical tests of paleontological hypoteses. Paleobiology. 2001; 27: 14–38.

[55] Reilly SM, Elias JA. Locomotion in *Alligator mississippiensis*: kinematic effects of speed and posture and their relevance to the sprawling-to-erect paradigm. The Journal of Experimental Biology. 1998; 201: 2559–2574. 9716509 10.1242/jeb.201.18.2559

[56] Baier DB, Gatesy SM. Three-dimensional skeletal kinematics of the shoulder girdle and forelimb in walking *Alligator*. Journal of Anatomy. 2013; 223: 462–473. doi: 10.1111/joa.12102 24102540 10.1111/joa.12102 PMC4399352

[57] Allen V, Molnar J, Parker W, Pollard A, Nolan G, Hutchinson JR. Comparative architectural properties of limb muscles in Crocodylidae and Alligatoridae and their relevance to divergent use of asymmetrical gaits in extant Crocodylia. Journal of Anatomy. 2014; 225: 569–582. doi: 10.1111/joa.12245 25418112 10.1111/joa.12245 PMC4262343

[58] Hutchinson JR, Anderson FC, Blemker SS, Delp SL. Analysis of hindlimb muscle moment arms in Tyrannosaurus rex using a three dimensional musculoskeletal computer model: implications for stance, gait and speed. Paleobiology. 2005; 31: 676–701.

[59] Wilson JA, Upchurch P. Redescription and reassessment of the phylogenetic affinities of *Euhelopus zdanskyi* (Dinosauria: Sauropoda) from the Early Cretaceous of China. Journal of Systematic Palaeontology. 2009; 7: 199–239.

[60] Gallina PA, Apesteguía S. Postcranial anatomy of *Bonitasaura salgadoi* (Sauropoda, Titanosauria) from the Late Cretaceous of Patagonia. Journal of Vertebrate Paleontology. 2015; e924957.

[61] Mateus O, Mannion PD, Upchurch P. *Zby atlanticus*, a new turiasaurian sauropod (Dinosauria, Eusauropoda) from the Late Jurassic of Portugal. Journal of Vertebrate Paleontology. 2014; 34: 618–634.

[62] Cong L, Hou L-H, Wu X. The gross anatomy of *Alligator sinensis* Fauvel. CIP, Beijing 1998; 388 pp.

[63] Bonaparte JF. 1999. Evolución de las vértebras presacras en Sauropodomorpha. Ameghiniana. 1999; 36: 115–189.

[64] Curry Rogers K. The postcranial osteology of *Rapetosaurus krausei* (Sauropoda: Titanosauria) from the late Cretaceous of Madagascar. Journal of Vertebrate Paleontology. 2009; 29: 1046–1086.

[65] Upchurch P, Barrett P, Dodson P. 2004 Sauropoda In: Weishampel DB, Dodson P, Osmólska H. The Dinosauria. Berkeley: University of California Press; 2004. pp. 259–322.

[66] Martínez RN. *Adeopapposaurus mognai*, gen. et sp. nov. (Dinosauria: Sauropodomorpha), with comments on adaptations of basal Sauropodomorpha. Journal of Vertebrate Paleontology. 2009; 29: 142–164.

[67] Otero A. The appendicular skeleton of *Neuquensaurus*, a Late Cretaceous saltasaurine sauropod from Patagonia, Argentina. Acta Palaeontologica Polonica. 2010; 55: 399–426.

[68] Sertich JJW, Loewen MA. A New Basal Sauropodomorph Dinosaur from the Lower Jurassic Navajo Sandstone of Southern Utah. PLoS ONE. 2010; 5: e9789 doi: 10.1371/journal.pone.0009789 20352090 10.1371/journal.pone.0009789 PMC2844413

[69] Marsh OC. Principal Characters of American Jurassic Dinosaurs, Part VI: Restoration of *Brontosaurus*. American Journal of Science. 183; 152: 81–85.

[70] von Huene F. Vollständige Osteologie eines Plateosauriden aus dem schwäbischen Keuper. *Geol*. *Palaeontol*. *Abhandl*. N. F. 1926; 15: 139–179.

[71] Yates AY, Vasconcelos CC. Furcula-like in the prosauropod dinosaur *Massospondylus*. Journal of Vertebrate Paleontology. 2005; 25: 466–468.

[72] Russell AP, Bauer AM. The appendicular locomotor apparatus of Sphenodon and normal-limbed squamates In: Gans C, Gaunt AS, Adler K, editors. Biology of the Reptilia 24, Morphology 1, Ithaca: Society for the Study of Amphibians and Reptiles 2008 pp. 1–466.

[73] Osborn HF, Mook CC. *Camarasaurus*, *Amphicoelias* and other sauropods of Cope. Memoirs of the American Museum of Natural History. 1921; 3: 247–387.

[74] Mannion PD, Otero A. A reappraisal of the Late Cretaceous Argentinean sauropod dinosaur *Argyrosaurus superbus*, with a description of a new titanosaur genus. Journal of Vertebrate Paleontology. 2012; 32: 614–638.

[75] Otero A, Pol D. Postcranial anatomy and phylogenetic relationships of *Mussaurus patagonicus* (Dinosauria, Sauropodomorpha). Journal of Vertebrate Paleontology. 2013; 33: 1138–1168.

[76] Taylor MP. A re-evaluation of *Brachiosaurus altithorax* Riggs 1903 (Dinosauria, Sauropoda) and its generic separation from *Giraffatitan brancai* (janensch 1914). Journal of Vertebrate Paleontology. 2009; 29: 787–806.

[77] Poropat SF, Upchurch P, Mannion PD, Hocknull SA, Kear BP, Sloan T, Sinapuis GHK, Elliott D. A. Revision of the sauropod dinosaur *Diamantinasaurus matildae* Hocknull et al. 2009 from the mid-Cretaceous of Australia: Implications for Gondwanan titanosauriform dispersal. Gondwana Research. 2015; 27: 995–1033.

[78] Mateus O, Jacobs LL, Schulp AS, Polcyn MJ, Tavares TS, Neto AB, et al *Angolatitan adamastor*, a new sauropod dinosaur and the first record from Angola. Anais da Academia Brasileira de Ciencias. 2011; 83: 221–233. 21437383 10.1590/s0001-37652011000100012

[79] You H-L, Li D-Q, Zhou L-Q, JI Q. *Daxiatitan binglingi*: a giant sauropod dinosaur from the Early Cretaceous of China. Gansu Geology. 2008; 17: 1–10.

[80] Bonaparte JF, González Riga BJ, Apesteguía S. *Ligabuesaurus leanzai* gen. et sp. nov. (Dinosauria, Sauropoda), a new titanosaur from the Lohan Cura Formation (Aptian, Lower Cretaceous) of Neuquén, Patagonia, Argentina. Cretaceous Research. 2006; 27: 364–376.

[81] Martin V, Suteethorn V, Buffetaut E. Description of the type and referred material of *Phuwiangosaurus sirindhornae* Martin, Buffetaut and Suteethorn, 1994, a sauropod from the Lower Cretaceous of Thailand. Oryctos. 1999; 2: 39–91.

[82] Harris JD. The appendicular skeleton of *Suuwassea emilieae* (Sauropoda: Flagellicaudata) from the Upper Jurassic Morrison Formation of Montana (USA). Geobios. 2007; 40: 501–522.

[83] Apaldetti CG, Pol D, Yates AM. The postcranial anatomy of *Coloradisaurus brevis* (Dinosauria: Sauropodomorpha) from the Late Triassic of Argentina and its phylogenetic implications. Palaeontology. 2012; 56: 277–301.

[84] VanBuren CS, Bonnan M. Forearm Posture and Mobility in Quadrupedal Dinosaurs. PLoS ONE. 2013; 8(9): e74842 doi: 10.1371/journal.pone.0074842 24058633 10.1371/journal.pone.0074842 PMC3776758

[85] Diogo R, Abdala V. Muscles of Vertebrates: Comparative Anatomy, Evolution, Homologies and Development. New Hampshire: Science Publishers; 2010.

[86] Gilmore CW. Osteology of *Apatosaurus* with special reference to specimens in the Carnegie Museum. Memoirs of the Carnegie Museum. 1936; 11: 175–300.

[87] Brochu CR. Osteology of *Tyrannosaurus rex*: insights from a nearly complete skeleton and high-resolution computed tomographic analysis of the skull. Society of Vertebrate Paleontology Memoir. 2002; 7: 1–138.

[88] Santa Luca AP. The postcranial skeleton of *Heterodontosaurus tucki* (Reptilia, Ornithischia) from the Stormberg of South Africa. Annals of the South African Museum. 1980; 79: 15–211.

[89] Alcober OA, Martinez RN. A new herrerasaurid (Dinosauria, Saurischia) from the Upper Triassic Ischigualasto Formation of northwestern Argentina. ZooKeys. 2010; 63: 55–81. 10.3897/zookeys.63.550 PMC3088398 21594020

[90] Tschopp E, Wings O, Frauenfelder T, Brinkmann W. Articulated bone sets of manus and pedes of *Camarasaurus* (Sauropoda, Dinosauria). Palaeontologia Electronica. 2015b; 18.2.44A: 1–65.

[91] Filippi LS, Canudo JI, Salgado JL, Garrido A, García R, Cerda I, et al A new sauropod titanosaur from the Plottier Formation (Upper Cretaceous) of Patagonia (Argentina). Geologica Acta. 2011; 9: 1–12.

[92] von Huene F. Los Saurisquios y Ornitisquios del Cretáceo Argentino. Museo de la Plata, Anales. 1929; Vol. 3: 194 pp.

[93] Sereno PC, Martínez RN, Alcober O. Osteology of *Eoraptor lunensis* (Dinosauria, Sauropodomorpha). Society of Vertebrate Paleontology Memoir. 2012; 12: 83–179.

[94] Leardi JM, Pol D, Novas FE, Suarez Riglos M. The postcranial anatomy of *Yacarerani boliviensis* and the phylogenetic significance of the notosuchian postcranial skeleton. Journal of Vertebrate Paleontology. 2015; 35: e995187.

[95] Sertich JJ, Groenke JR. Appendicular skeleton of *Simosuchus clarki* (Crocodyliformes: Notosuchia) from the Late Cretaceous of Madagascar. In: Krause DW, Kley NJ, editors. *Simosuchus clarki* (Crocodyliformes: Notosuchia) from the Late Cretaceous of Madagascar. Society of Vertebrate Paleontology Memoir. 2010; 10: 122–153.

[96] Holtz TR. Tyrannosauroidea In: Weishampel DB, Dodson P, Osmólska H. The Dinosauria. Berkeley: University of California Press; 2004 pp. 111–136.

[97] Horner JR, Weishampel DB, Forster CA. 2004 Hadrosauridae In: Weishampel DB, Dodson P, Osmólska H. The Dinosauria. Berkeley: University of California Press; 2004. pp. 438–463.

[98] Osmólska H, Currie PJ, Barsbold R. Oviraptorosauria In: Weishampel DB, Dodson P, Osmólska H. The Dinosauria. Berkeley: University of California Press; 2004 pp. 165–183.

[99] Burch SH, Carrano MT. An articulated pectoral girdle and forelimb of the abelisaurid theropod *Majungasaurus crenatissimus* from the Late Cretaceous of Madagascar. Journal of Vertebrate Paleontology. 2012; 32: 1–16.

[100] Wilson JA, Sereno PC. Early evolution and higher-level phylogeny of sauropod dinosaurs. Society of Vertebrate Paleontology Memoir. 1998; 5: 1–68.

[101] Nesbitt SJ. The early evolution of archosaurs: relationships and the origin of major clades. Bulletin of the American Museum of Natural History Number. 2011; 352: pp. 292.

[102] Maidment SCR, Norman DB, Barrett PM, Upchurch P. Systematics and phylogeny of Stegosauria (Dinosauria: Ornithischia). Journal of Systematic Palaeontology. 2008; 6; 367–407.

[103] Sereno PC. The evolution of dinosaurs. Science. 1999; 284: 2137–2147. 10381873 10.1126/science.284.5423.2137

[104] Gower DJ, Schoch RR. Postcranial anatomy of the rauisuchian archosaur *Batrachotomus kupferzellensis*. Journal of Vertebrate Paleontology. 2009; 29: 103–122.

[105] Carrano MT, Hutchinson JR, Sampson SD. New information on *Segisaurus halli*, a small theropod dinosaur from the early Jurassic of Arizona. Journal of Vertebrate Paleontology. 2005 25: 835–849.

[106] Benson RBJ. A description of *Megalosaurus bucklandii* (Dinosauria: Theropoda) from the Bathonian of the UK and the relationships of Middle Jurassic theropods. Zoological Journal of the Linnean Society. 2010; 158: 882–935.

[107] Nesbitt SJ, Stocker MR, Small BJ, Downs A. The osteology and relationships of *Vancleavea campi* (Reptilia: Archosauriformes). Zoological Journal of the Linnean Society 2009; 157: 814–864.

[108] Bittencourt JS, Arcucci AB, Marsicano CA, Langer MC. Osteology of the Middle Triassic archosaur *Lewisuchus admixtus* Romer (Chañares Formation, Argentina), its inclusivity, and relationships amongst early dinosauromorphs. Journal of Systematic Palaeontology. 2014; 13: 189–219.

[109] Sereno PC. The pectoral girdle and forelimb of the basal theropod *Herrerasaurus ischigualastensis*. Journal of Vertebrate Paleontology. 1993; 13: 425–450.

[110] Butler RJ. The anatomy of the basal ornithischian dinosaur *Eocursor parvus* from the lower Elliot Formation (Late Triassic) of South Africa. Zoological Journal of the Linnean Society. 2010; 160: 648–684.

[111] Langer MC, Ferigolo J. The Late Triassic dinosauromorph *Sacisaurus agudoensis* (Caturrita Formation; Rio Grande do Sul, Brazil): anatomy and affinities In: Nesbitt S J, Desojo JB, Irmis RB, editors. Anatomy, Phylogeny and Palaeobiology of Early Archosaurs and their Kin. London: Geological Society, Special Publications; 2013 379.

[112] Currie PJ, Zhao X-J. A new carnosaur (Dinosauria, Theropoda) from the Jurassic of Xinjiang, People’s Republic of China. Canadian Journal of Earth Sciences. 1994; 30: 2037–2081.

[113] Madsen JR Jr. *Allosaurus fragilis*: a revised osteology. Utah Geological Survey Bulletin. 1993; 109: pp. 163.

[114] Leardi JM. Evolución de la función locomotora durante la diversificación de Archosauria: Patrones de cambio antómico-funcional de la cintura escapular y el miembro anterior. Facultad de Ciencias Exactas y Naturales. Universidad de Buenos Aires. 2013. Pp. 620.

[115] Pol D. Postcranial remains of *Notosuchus terrestris* (Archosauria: Crocodyliformes) from the upper Cretaceous of Patagonia, Argentina. Ameghiniana. 2005; 42: 21–38.

[116] Godoy PL, Bronzati M, Eltink E, Marzola JCA, Cidade GM, Langer MC, et al Postcranial anatomy of *Pissarrachampsa* sera (Crocodyliformes, Baurusuchidae) from the Late Cretaceous of Brazil: insights on lifestyle and phylogenetic significance. PeerJ. 2016; 4: e2075 doi: 10.7717/peerj.2075 27257551 10.7717/peerj.2075 PMC4888301

[117] Clark JM, Marianska T, Barsbold R. Therizinosauroidea In: Weishampel DB, Dodson P, Osmólska H, editors. The Dinosauria. Berkeley: University of California Press; 2004; pp. 151–164.

[118] Osborn HF. A skeleton of *Diplodocus*. Memoirs of the American Museum of Natural History. 1899; 30: 1–165.

[119] McIntosh JS. The sauropod dinosaurs: a brief survey In: Padian K, Chure DJ, editors. The age of dinosaurs, short courses in Paleontology number 2. Knoxville: University of Tennessee; 1989; pp. 85–99.

[120] Wilson JA. A nomenclature for vertebral laminae in sauropods and other saurischian dinosaurs. Journal of Vertebrate Paleontology. 1999; 19: 639–653.

[121] Wedel MJ. What pneumaticity tells us about ‘prosauropods’, and vice versa. Special Papers in Palaeontology. 2007; 77: 207–222.

[122] Wedel MJ. Vertebral pneumaticity, air sacs, and the physiology of sauropod dinosaurs. Paleobiology. 2003; 29: 243–255.

[123] Wedel MJ. Postcranial skeletal pneumaticity in sauropods and its implications for mass estimates In: Wilson JA, Curry-Rogers K, editors. The sauropods: evolution and paleobiology. Berkeley: University of California Press 2005 pp. 201–228.

[124] Wedel MJ. Evidence for bird-like air-sacs in saurischian dinosaurs. Journal of Experimental Zoology A. 2009; 311: 611–628. 10.1002/jez.513 19204909

[125] Cerda IA, Salgado L, Powell JE. Extreme postcranial pneumaticity in sauropod dinosaurs from South America. Paläontologische Zeitschrift. 2012b; 86: 441–449.

[126] Wedel MJ, Sanders KR. Osteological correlates in Aves and Sauropoda (Dinosauria: Saurischia), with comments on the cervical ribs of *Apatosaurus*. Paleobios. 2002; 22: 1–6.

[127] Pol D, Garrido A, Cerda IA. A New Sauropodomorph Dinosaur from the Early Jurassic of Patagonia and the Origin and Evolution of the Sauropod type sacrum. PLoS ONE. 2011; 6(1): e14572 doi: 10.1371/journal.pone.0014572 21298087 10.1371/journal.pone.0014572 PMC3027623

[128] Jenkins FAJ. The evolution of the avian shoulder joint. American Journal of Science. 1993; 293-a: 253–267.

[129] Martinez RN, Alcober OA. A Basal Sauropodomorph (Dinosauria: Saurischia) from the Ischigualasto Formation (Triassic, Carnian) and the Early Evolution of Sauropodomorpha. PLoS ONE. 2009; 4(2): e4397 doi: 10.1371/journal.pone.0004397 19209223 10.1371/journal.pone.0004397 PMC2635939

[130] Apaldetti C, Martinez RN, Alcober OA, Pol D. A New Basal Sauropodomorph (Dinosauria: Saurischia) from Quebrada del Barro Formation (Marayes-El Carrizal Basin), Northwestern Argentina. PLoS ONE. 2011; 6(11): e26964 doi: 10.1371/journal.pone.0026964 22096511 10.1371/journal.pone.0026964 PMC3212523

[131] Wilson JA, D’Emic MD, Ikejiri T, Moacdieh EM, Whitlock JA. A Nomenclature for Vertebral Fossae in Sauropods and Other Saurischian Dinosaurs. 2011; PLoS ONE 6(2): e17114 doi: 10.1371/journal.pone.0017114 21386963 10.1371/journal.pone.0017114 PMC3046170

[132] Torcida Fernández-Baldor F, Canudo JI, Huerta P, Moreno-Azanza M, Montero D. *Europatitan eastwoodi*, a new sauropod from the lower Cretaceous of Iberia in the initial radiation of somphospondylans in Laurasia. PeerJ. 2017; 5:e3409; doi: 10.7717/peerj.3409 28674644 10.7717/peerj.3409 PMC5490465

[133] Mannion PD, Allain R, Moine O. The earliest known titanosauriform sauropod dinosaur and the evolution of Brachiosauridae. PeerJ. 2017; 5:e3217; doi: 10.7717/peerj.3217 28480136 10.7717/peerj.3217 PMC5417094

